# Supplementary material for: Synthesis of Novel Indole Schiff Base Compounds and Their Antifungal Activities
Source: Molecules. 2022 Oct 13;27(20):6858. doi: 10.3390/molecules27206858 (PMC9609699; doi:10.3390/molecules27206858)
Supplement: Supplementary file 1 [file molecules-27-06858-s001.zip › molecules-1951541-supplementary.pdf]

Comounication

Synthesis of Novel Indole Schiff Base Compounds and Their Antifungal Activities

[1]Caixia Wang, Liangxin Fan, Zhenliang Pan, Sufang Fan, Lijun Shi, Xu Li, Jinfang Zhao, Wu Lulu,Guoyu Yang* and Cuilian Xu*

Ⅰ.The IR, ^{1}H NMR, ^{13}C NMR and HRMS of the target compounds (2a-2t)

Figure S1-S80; page: 2-41.

Ⅱ.The physical photos of the inhibitory activity of the target compounds against test fungi, Figure S81-S85; page: 42-46.

Ⅰ. The IR, ¹H NMR, ¹³C NMR and HRMS of the target compounds (2a-2t)

Figure S1-S80:

1.The IR, ¹H NMR, ¹³C NMR and HRMS of the compound 2a

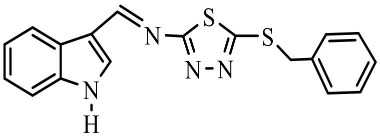

$C_{18}H_{14}N_4S_2$, orange yellow crystals, m.p.: 200.5-201.4 °C.

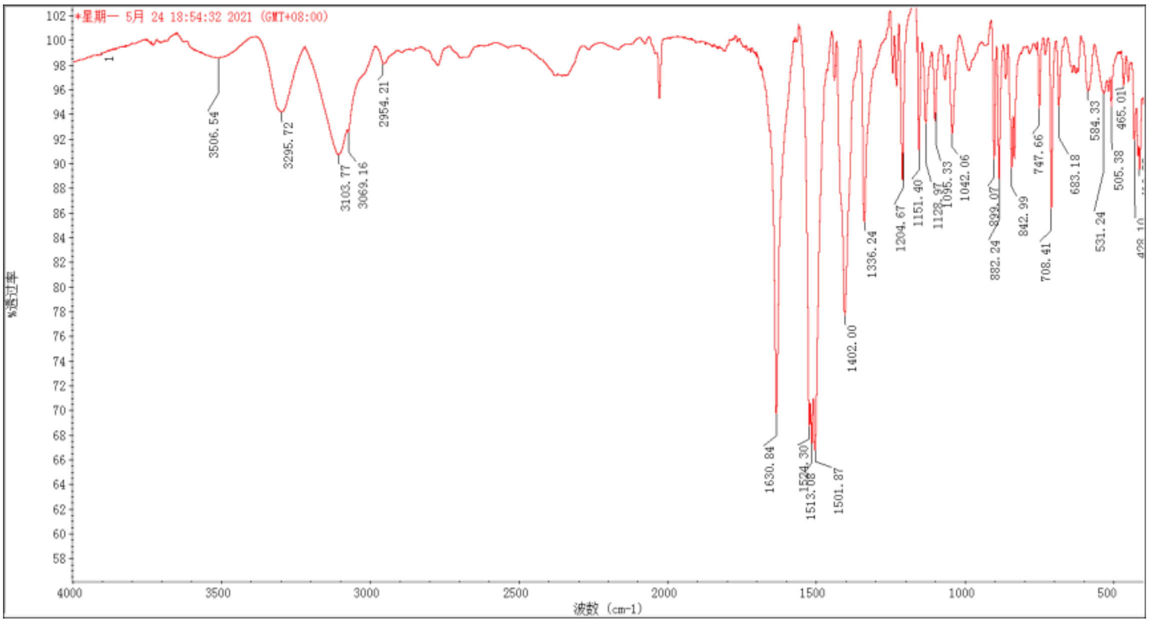

Figure S1. IR spectra of the compound **2a**

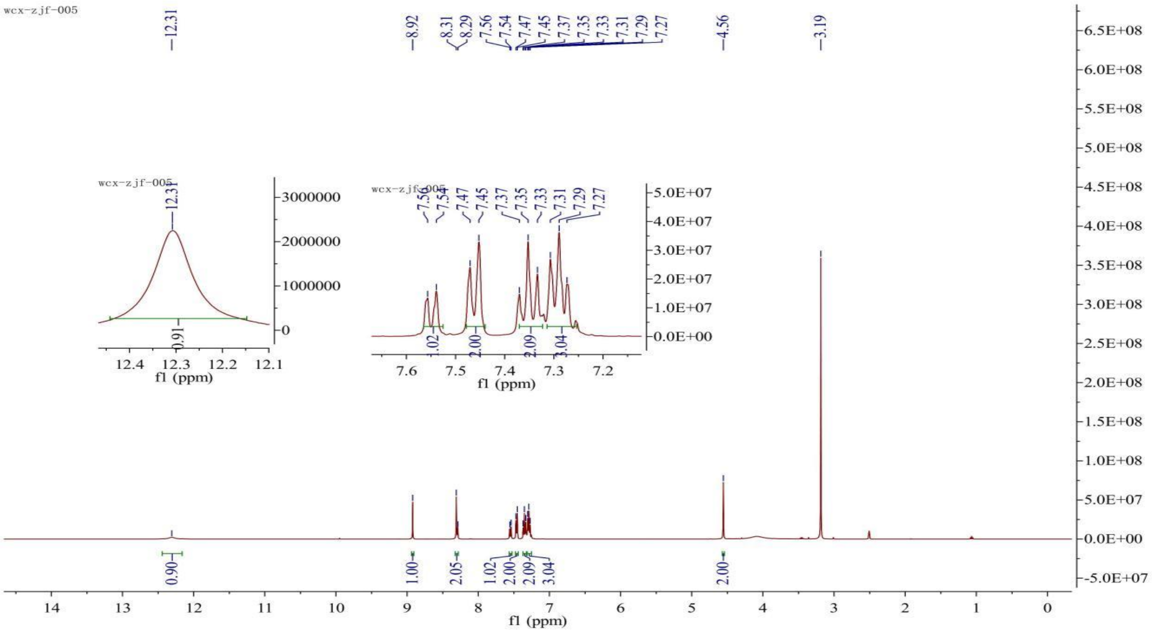

Figure S2. ¹H NMR spectra of the compound **2a** (DMSO)

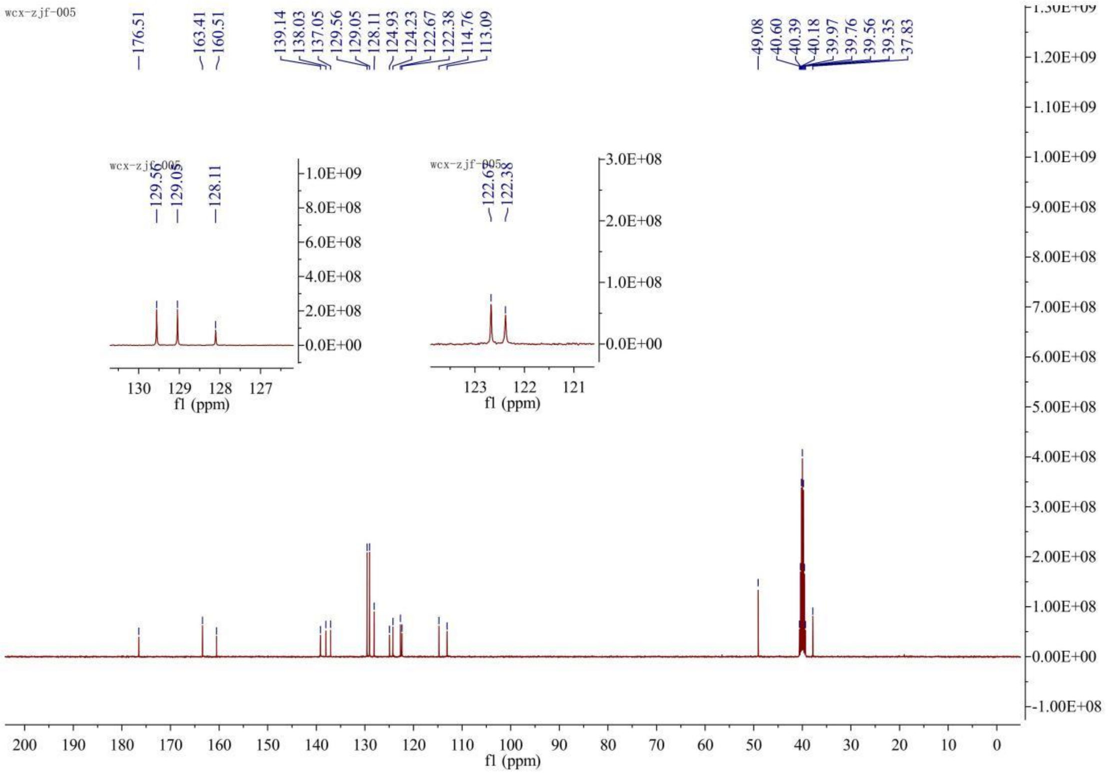

Figure S3. ^{13}C NMR spectra of the compound **2a (**DMSO**)**

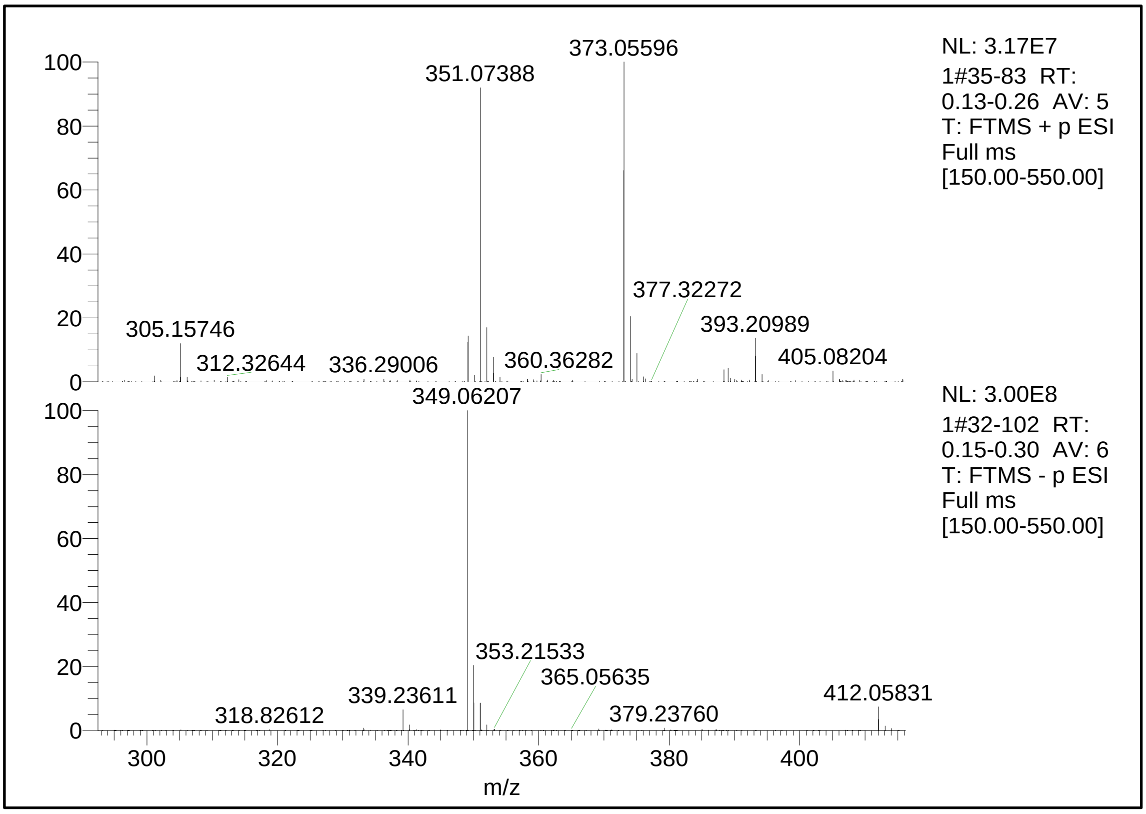

Figure S4. HR-MS of the compound **2a**

Calcd for $C_{18}H_{14}N_4S_2$ $[M + Na]^+$: 373.0558; found 373.0560.

2.The IR, ^{1}H NMR, ^{13}C NMR and HRMS of the compound 2b

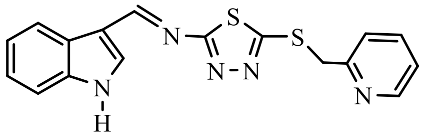

$C_{17}H_{13}N_5S_2$, yellow needle crystal; m.p.209.1-210.5 ℃.

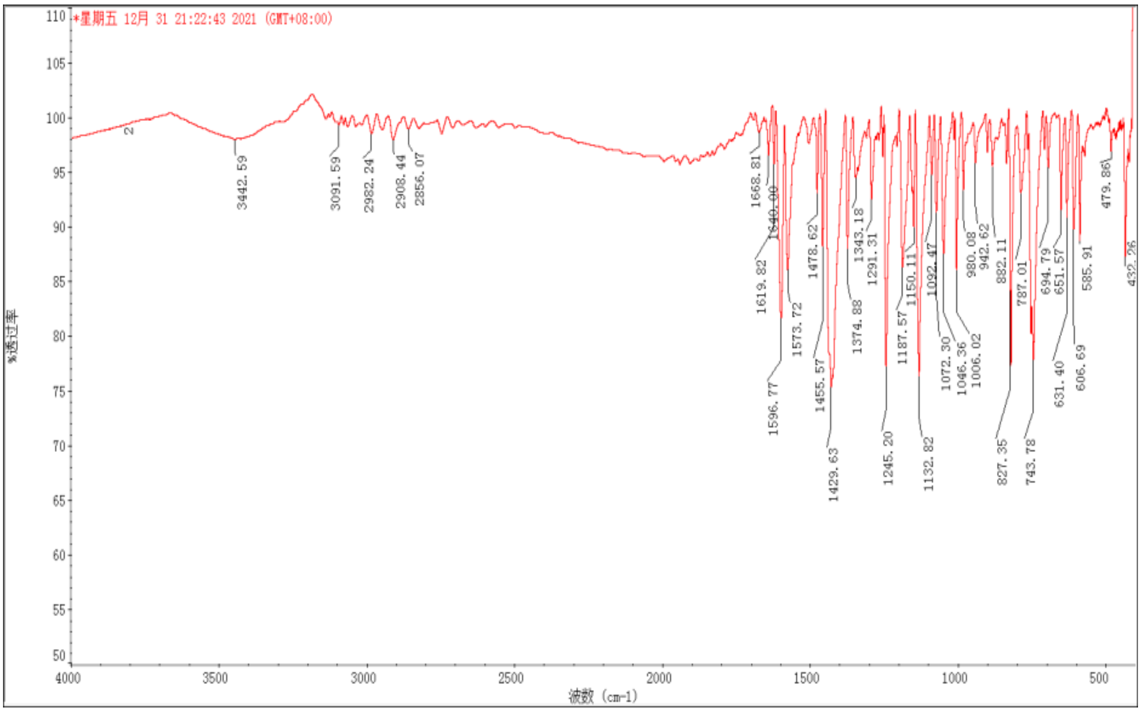

Figure S5. IR spectra of the compound **2b**

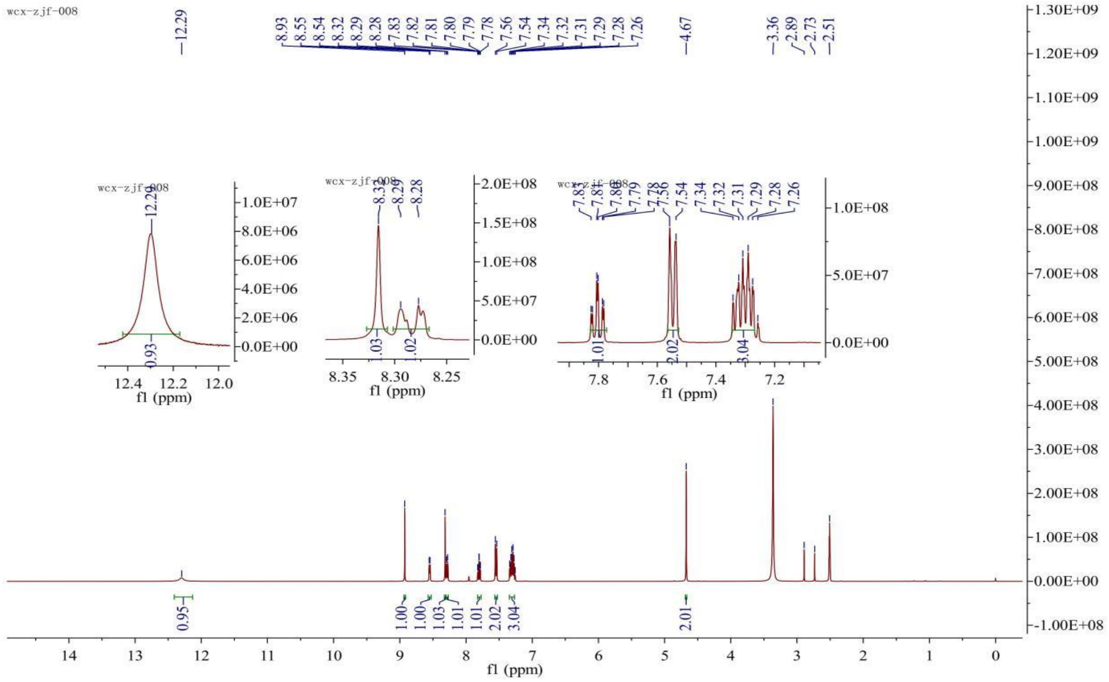

Figure S6. ^{1}H NMR spectra of the compound **2b** (DMSO)

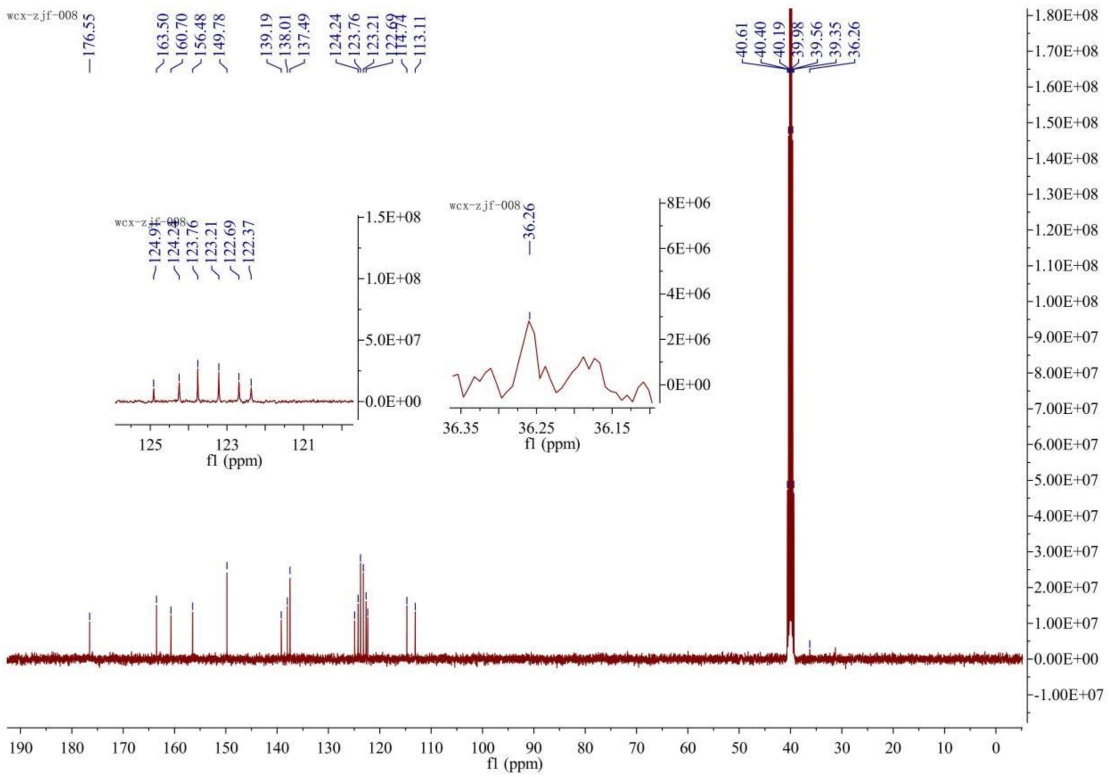

Figure S7. ^{13}C NMR spectra of the compound **2b** (DMSO)

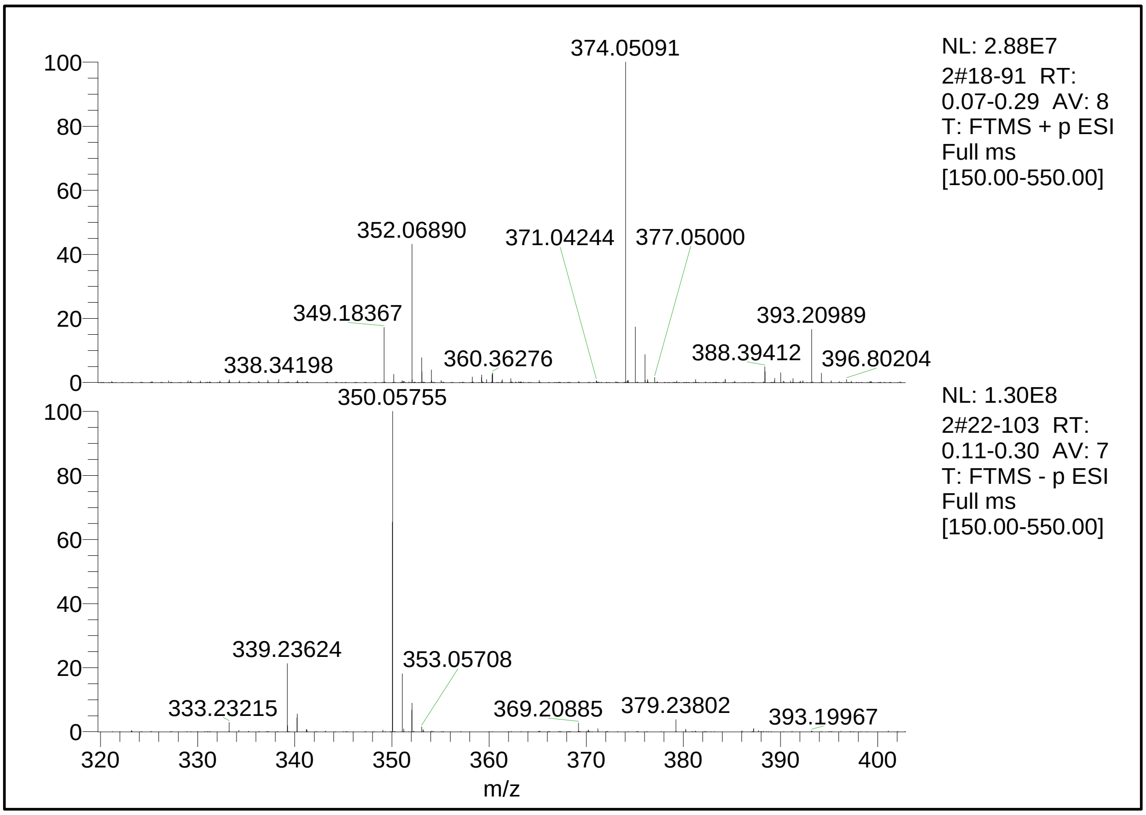

Figure S8. HR-MS of the compound **2b**

Calcd for $C_{17}H_{13}N_5S_2$ $[M + Na]^+$: 374.0510; found 374.0509.

.

3. The IR, ^{1}H NMR, ^{13}C NMR and HRMS of the compound 2c

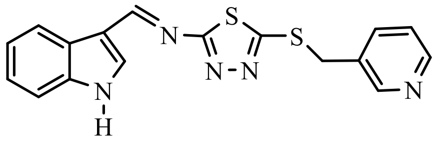

$C_{17}H_{13}N_5S_2$, yellow-green needle-shaped crystal; m.p. 207.5-208.4 ℃.

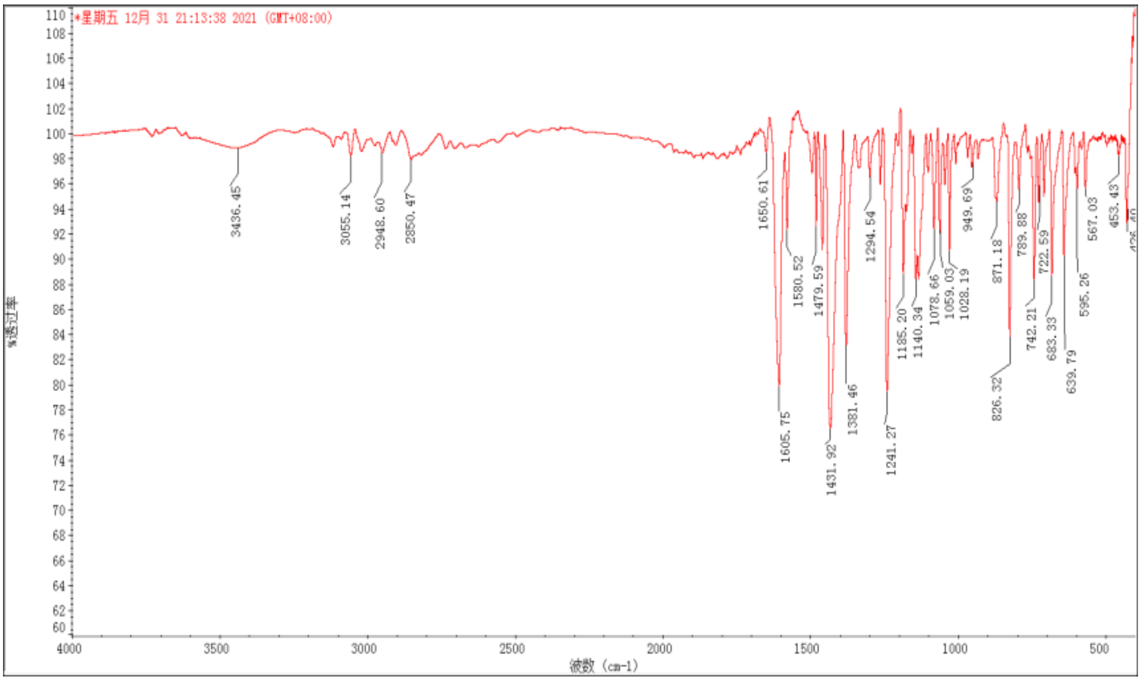

Figure S9. IR spectra of the compound **2c**

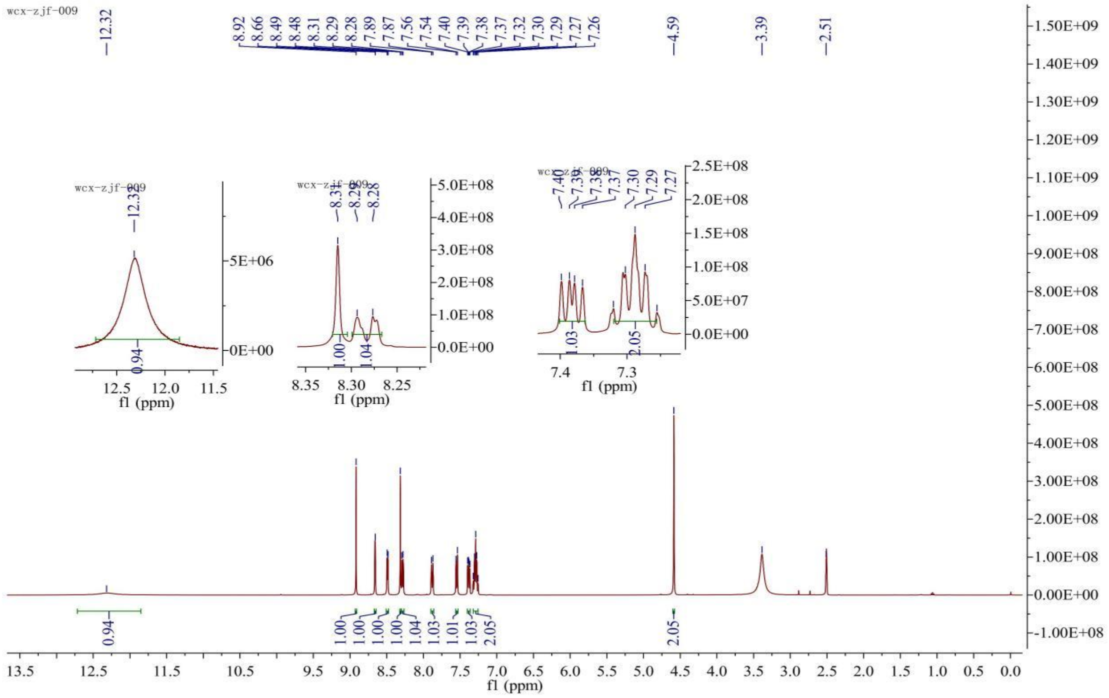

Figure S10. ^{1}H NMR spectra of the compound **2c** (DMSO)

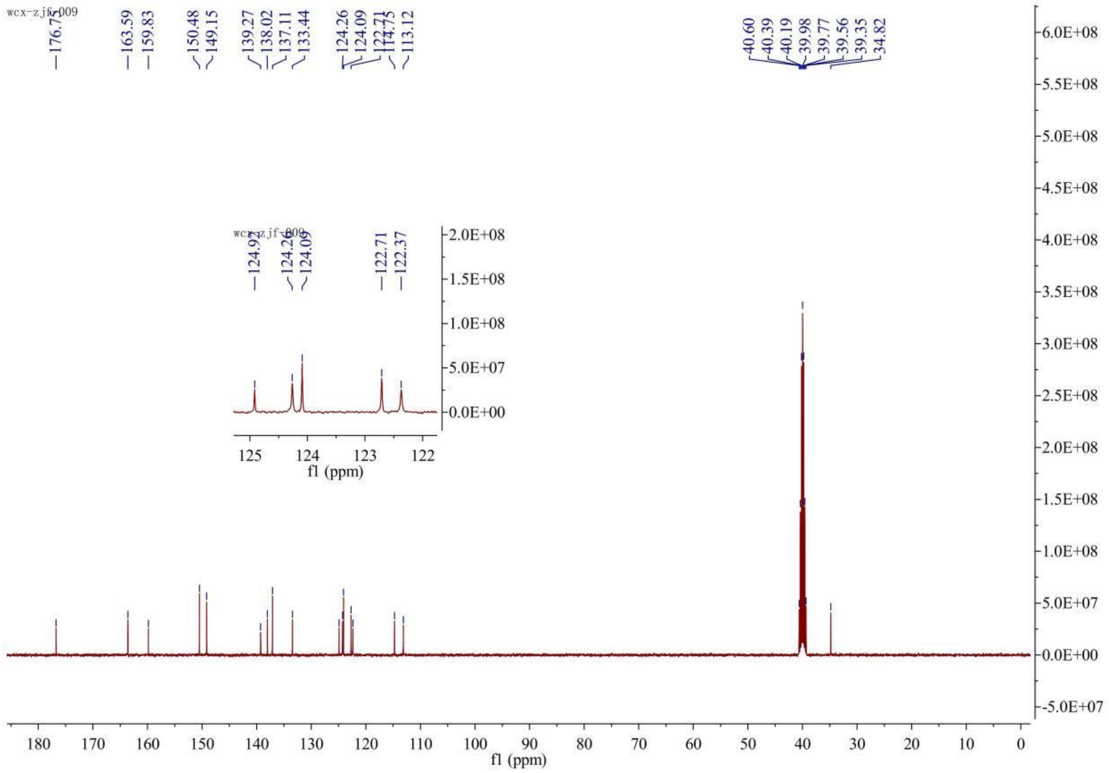

Figure S11. ^{13}C NMR spectra of the compound **2c** (DMSO)

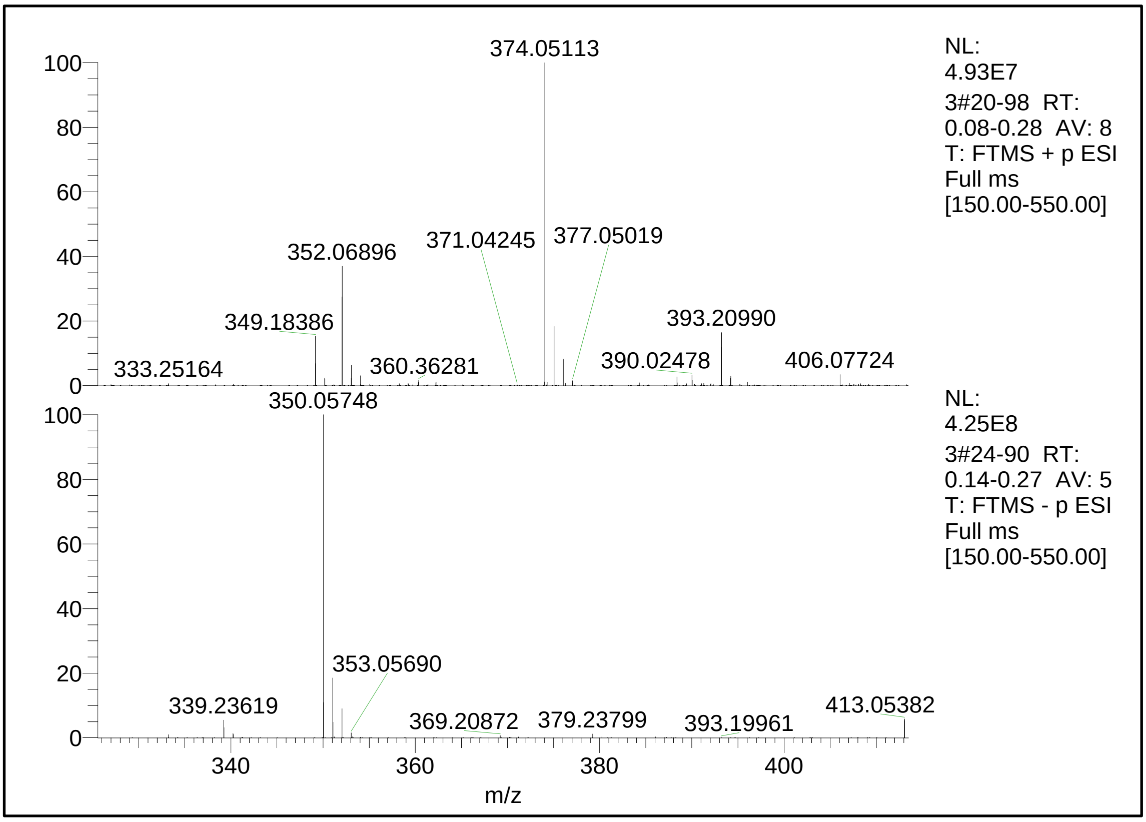

Figure S12. HR-MS of the compound **2c**

Calcd for $C_{17}H_{13}N_5S_2$ $[M + Na]^+$: 374.0510; found 374.0511.

4.The IR, ^{1}H NMR, ^{13}C NMR and HRMS of the compound 2d

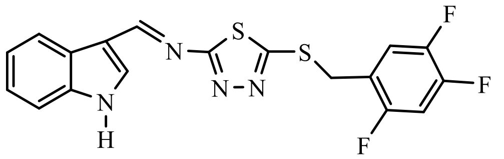

$C_{18}H_{11}F_3N_4S_2$, bright yellow needle-shaped crystal; m. p. 206.2-207.5 ℃.

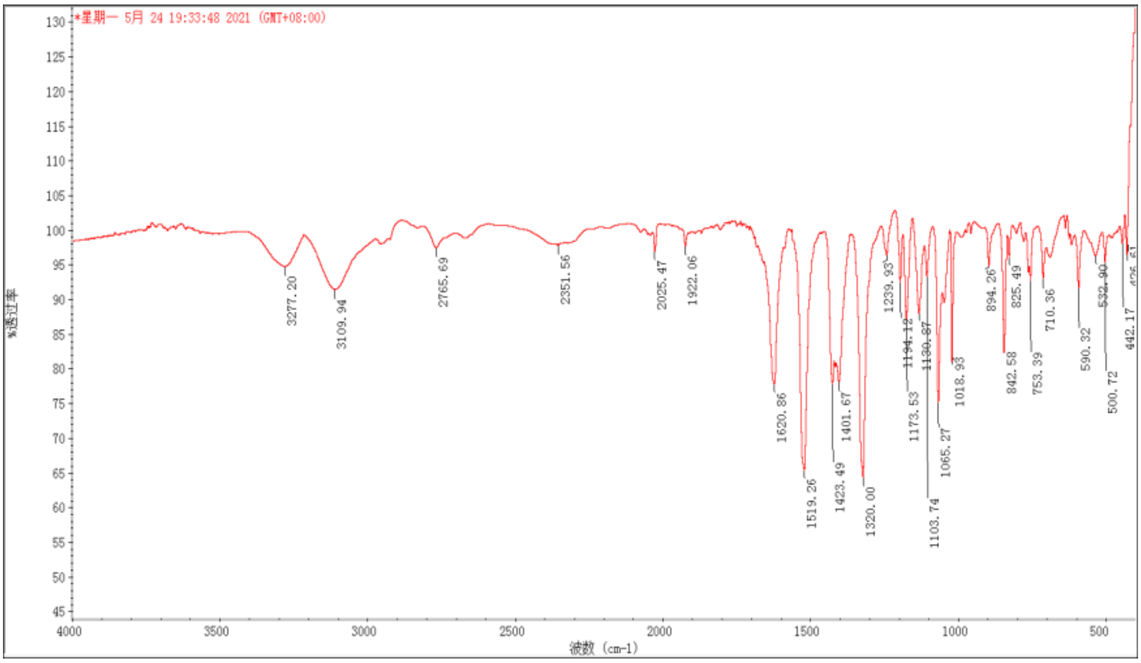

Figure S13. IR spectra of the compound **2d**

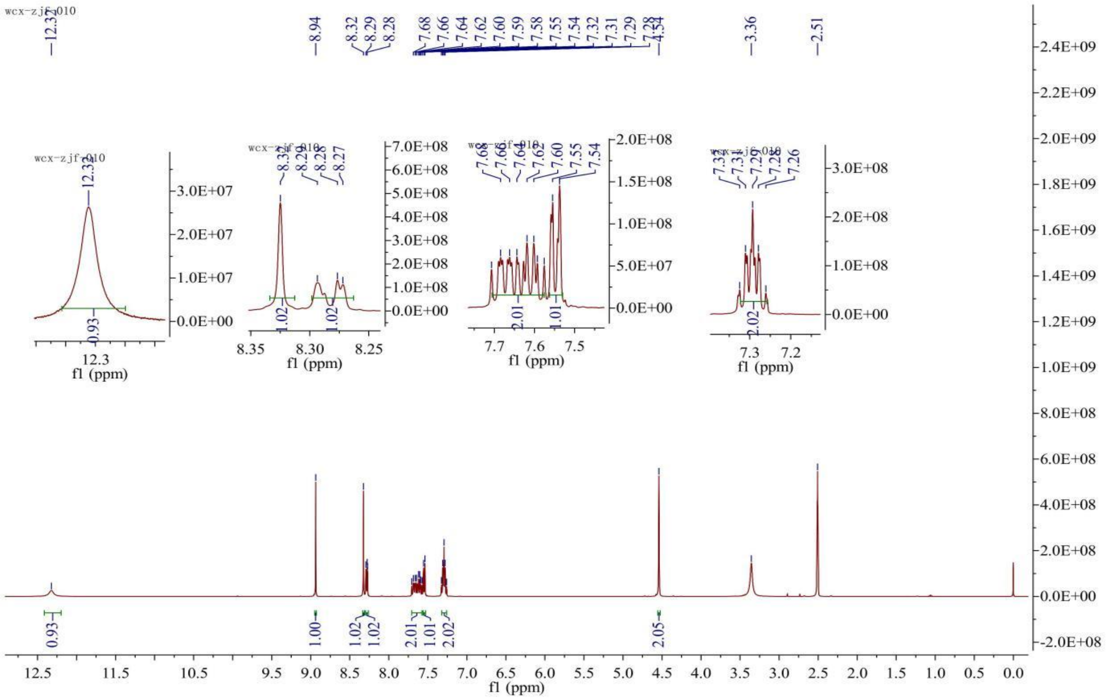

Figure S14. ^{1}H NMR spectra of the compound **2d (**DMSO**)**

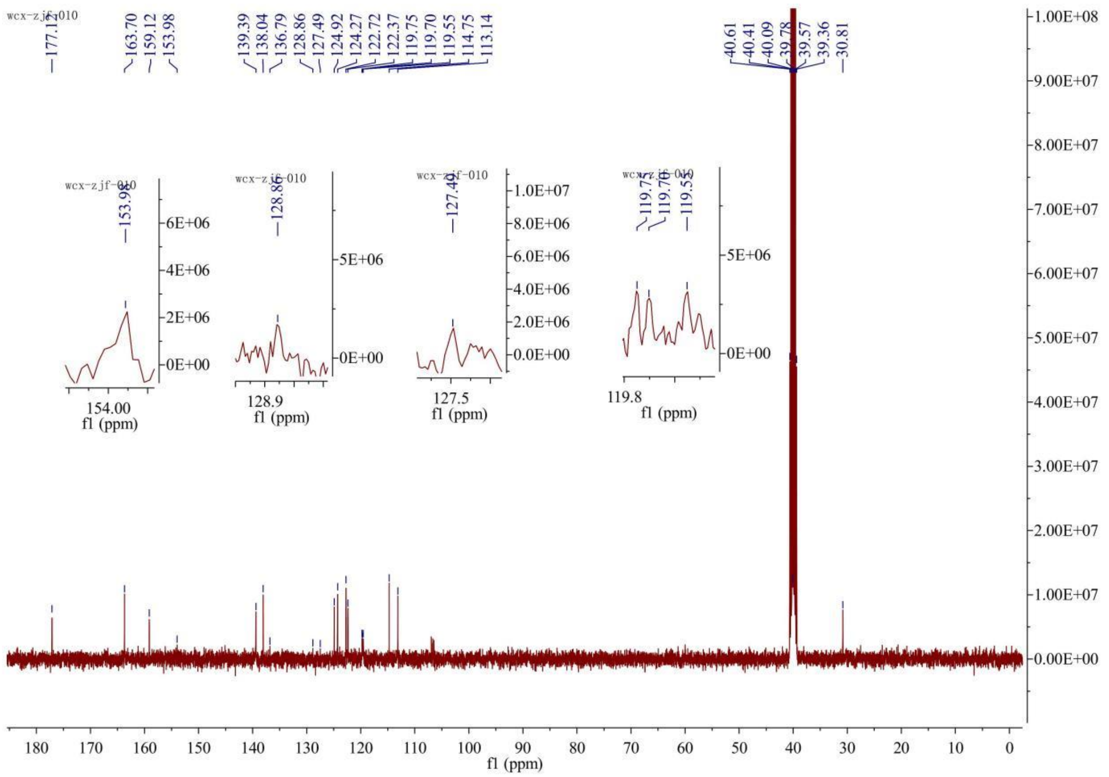

Figure S15. ^{13}C NMR spectra of the compound **2d** (DMSO)

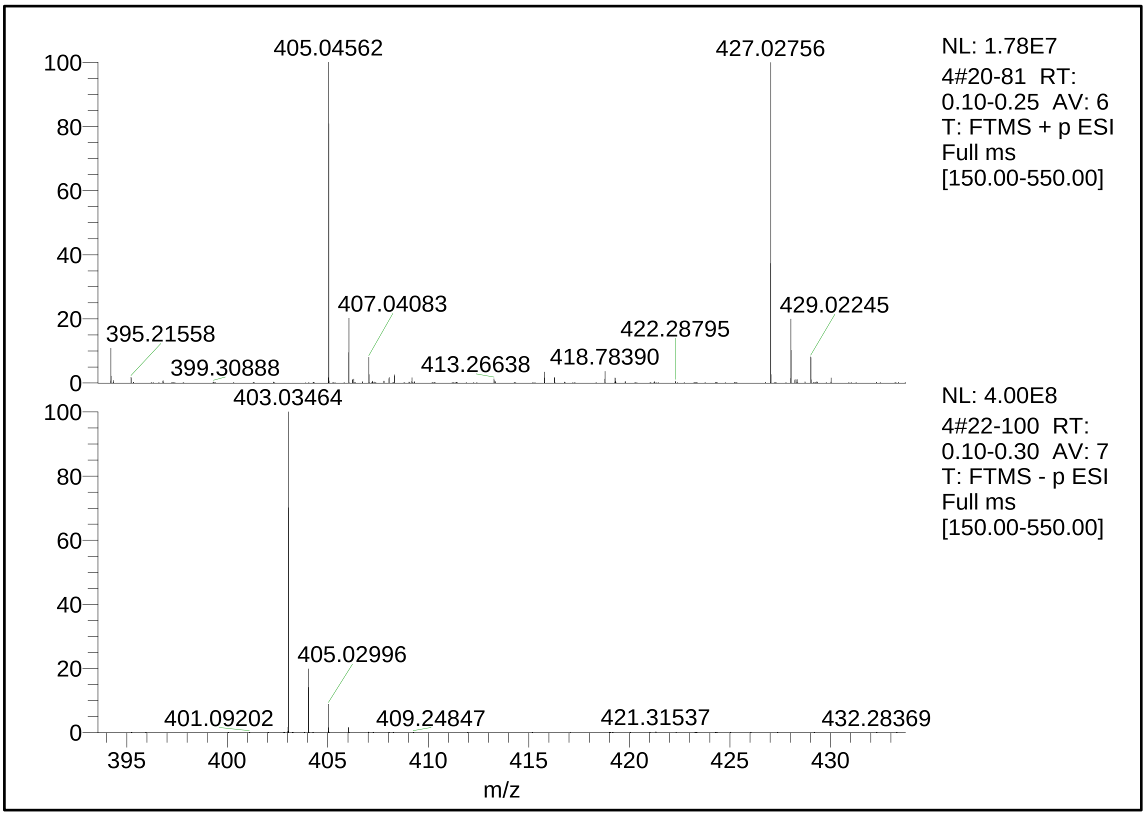

Figure S16. HR-MS of the compound **2d**

Calcd for $C_{18}H_{11}F_3N_4S_2$ [M + Na]$^+$: 427.0275; found 427.0276.

5.The IR, ^{1}H NMR, ^{13}C NMR and HRMS of the compound 2e

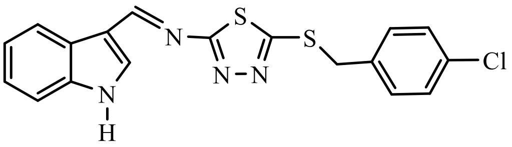

$C_{18}H_{13}ClN_4S_2$, beige needle crystal; m. p. 201.8-202.6 ℃.

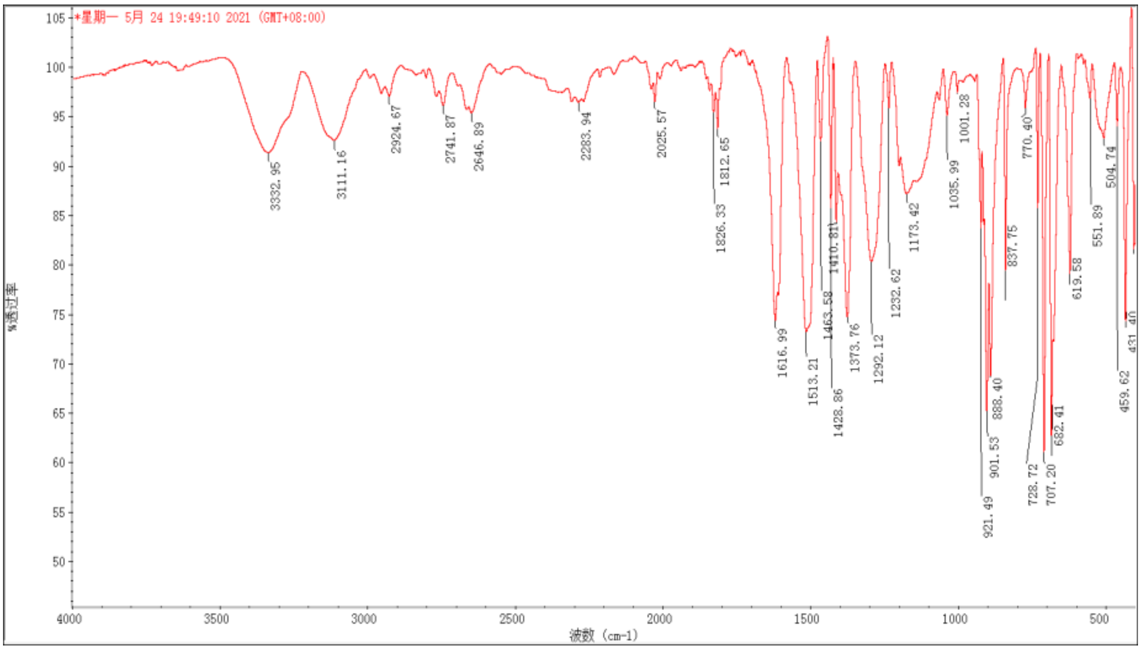

Figure S17. IR spectra of the compound **2e**

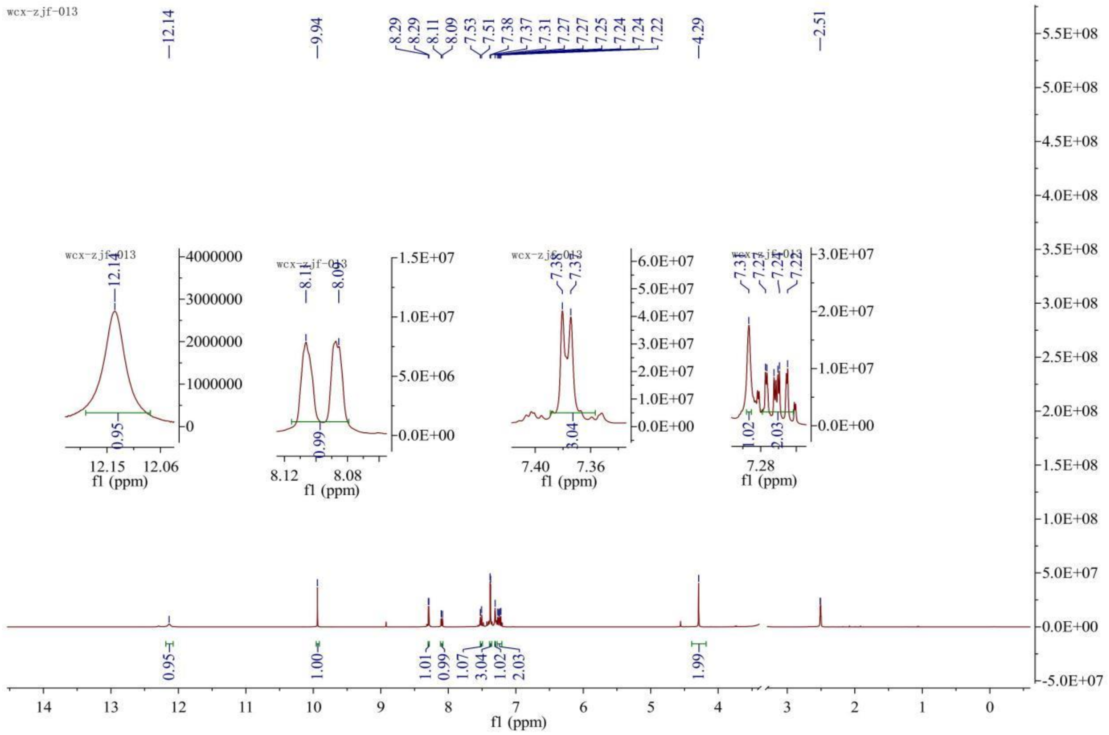

Figure S18. ^{1}H NMR spectra of the compound **2e** (DMSO)

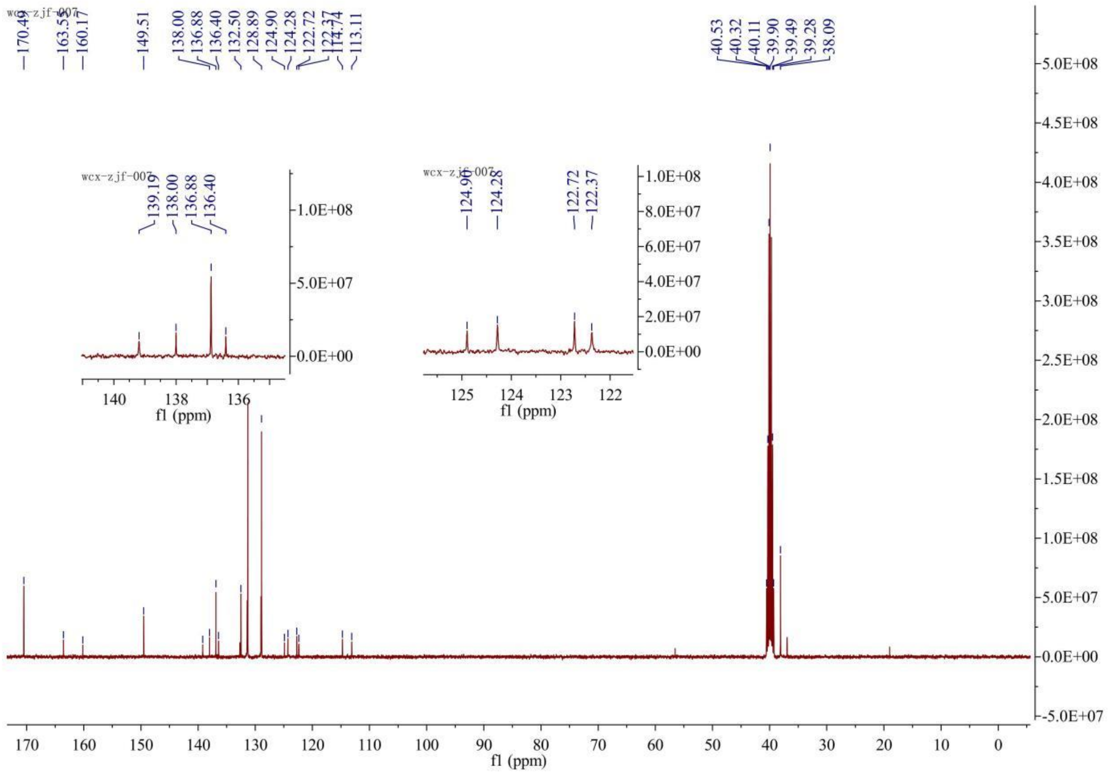

Figure S19. ^{13}C NMR spectra of the compound 2e **(**DMSO**)**

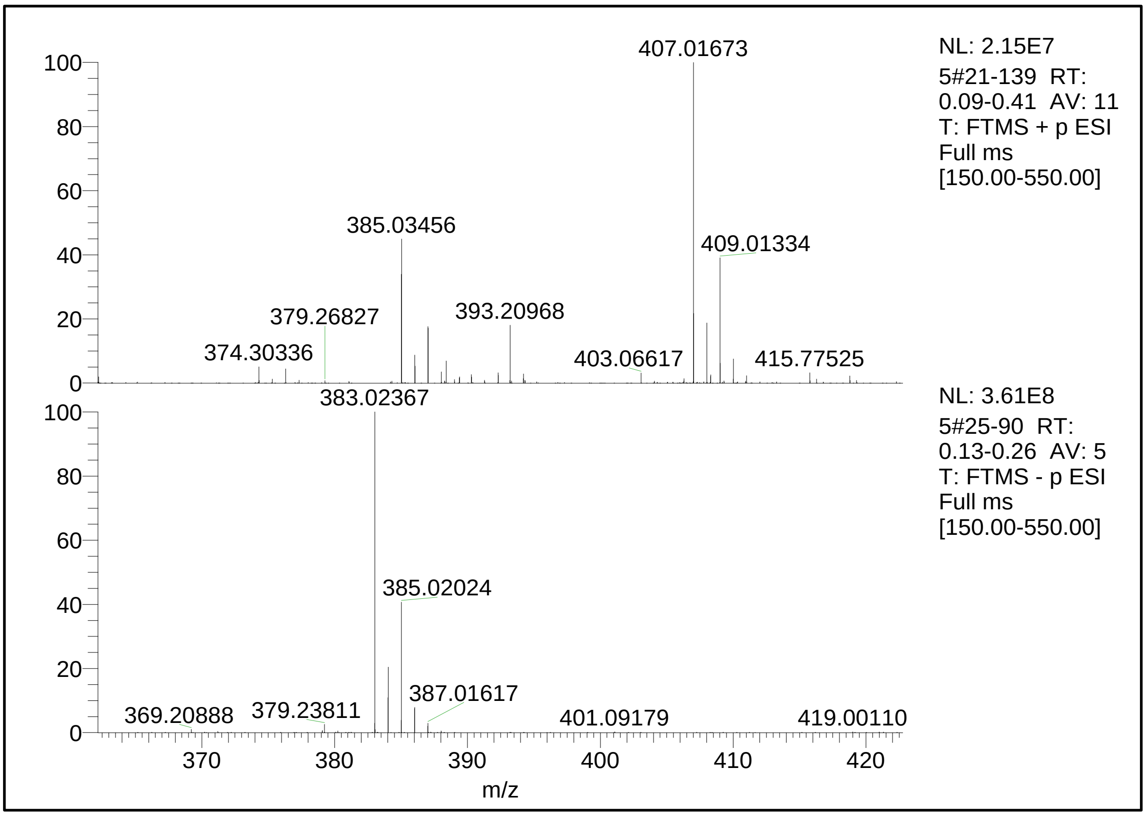

Figure S20. HR-MS of the compound **2e**

Calcd for $C_{18}H_{13}ClN_4S_2$ $[M + Na]^+$: 407.0168; found 407.0167.

6.The IR, ^{1}H NMR, ^{13}C NMR and HRMS of the compound 2f

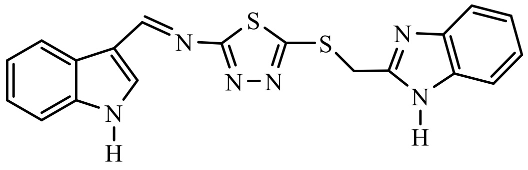

$C_{19}H_{14}N_6S_2$, brown-red needle-shaped crystals; m. p. 245.8-246.5 ℃.

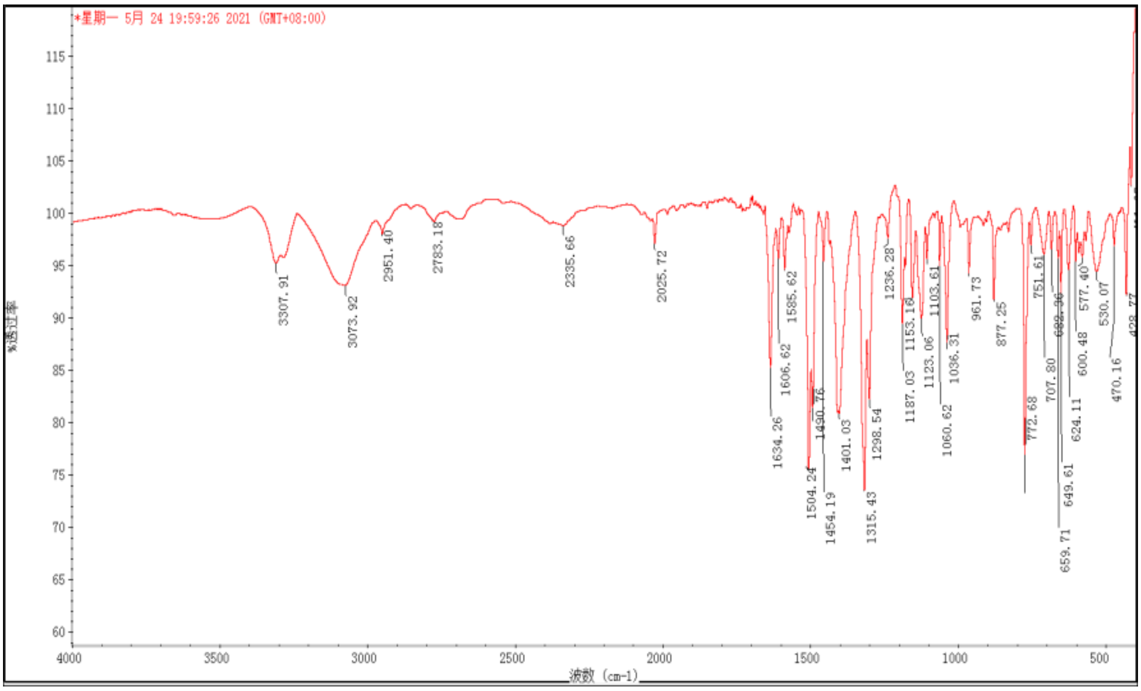

Figure S21. IR spectra of the compound **2f**

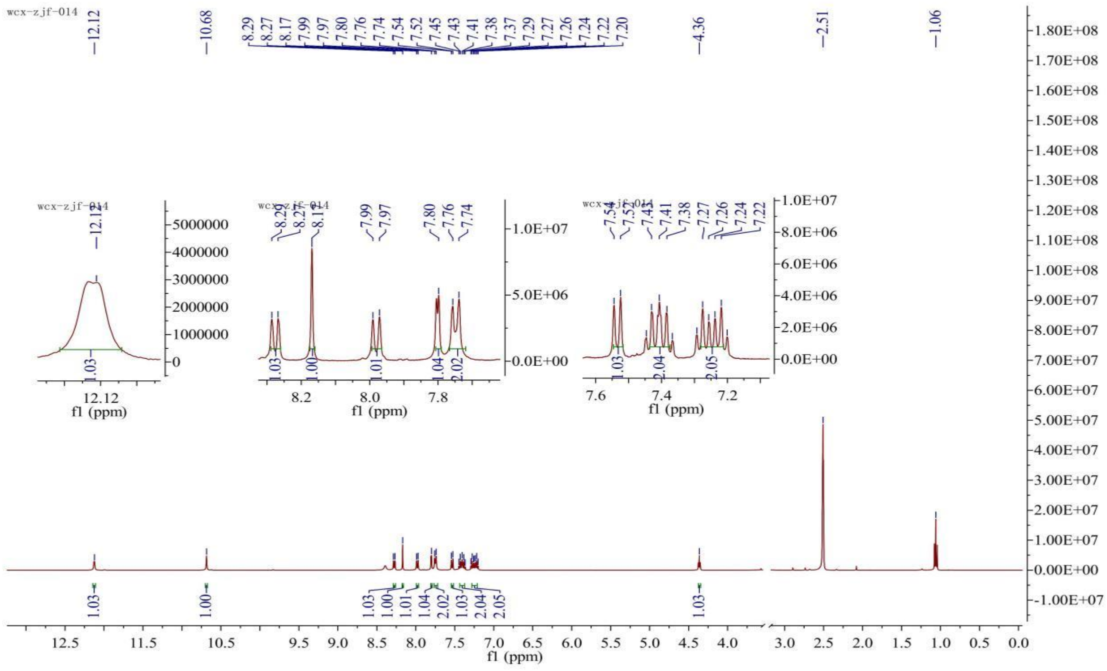

Figure S22. ^{1}H NMR spectra of the compound **2f (**DMSO**)**

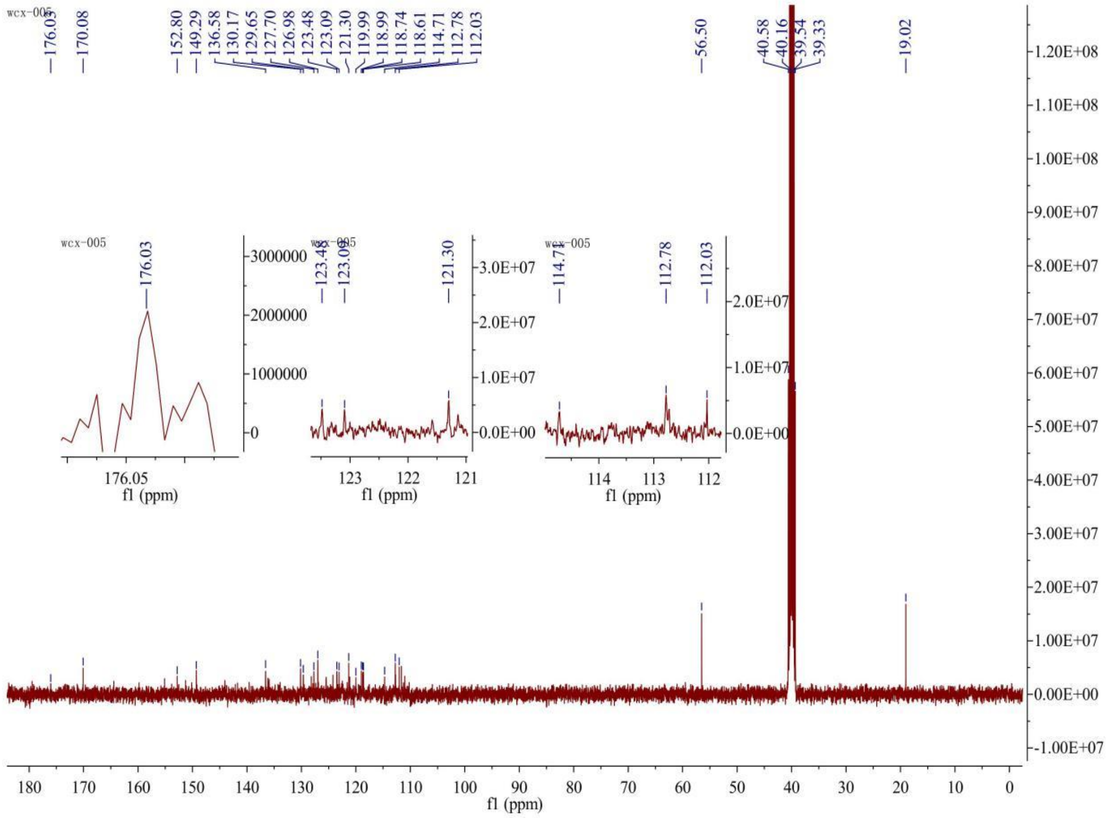

Figure S23. ^{13}C NMR spectra of the compound **2f** (DMSO)

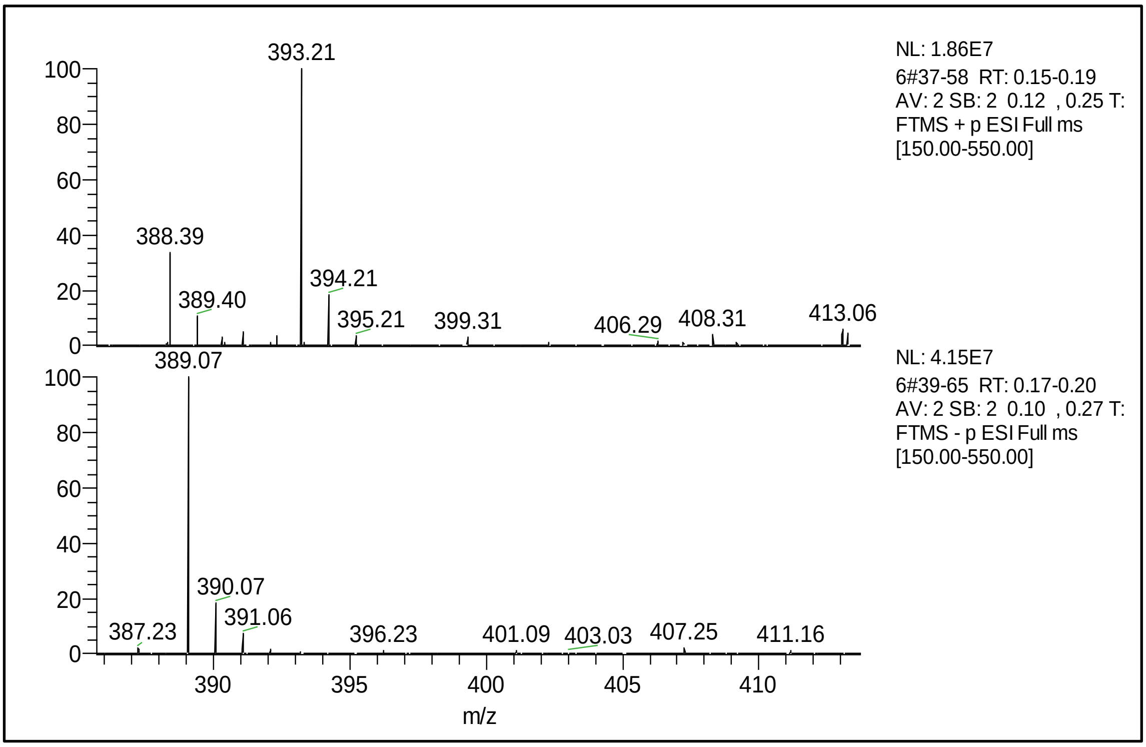

Figure S24. HR-MS of the compound **2f**

Calcd for $C_{19}H_{13}N_6S_2$ [M-H]$^{+}$: 389.0643; found 389.0700.

7.The IR, ^{1}H NMR, ^{13}C NMR and HRMS of the compound 2g

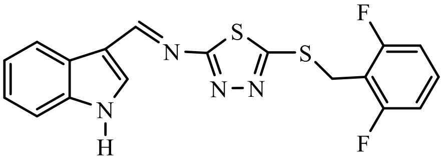

$C_{18}H_{12}F_2N_4S_2$, light yellow solid powder；m. p. 177.0-177.7 ℃.

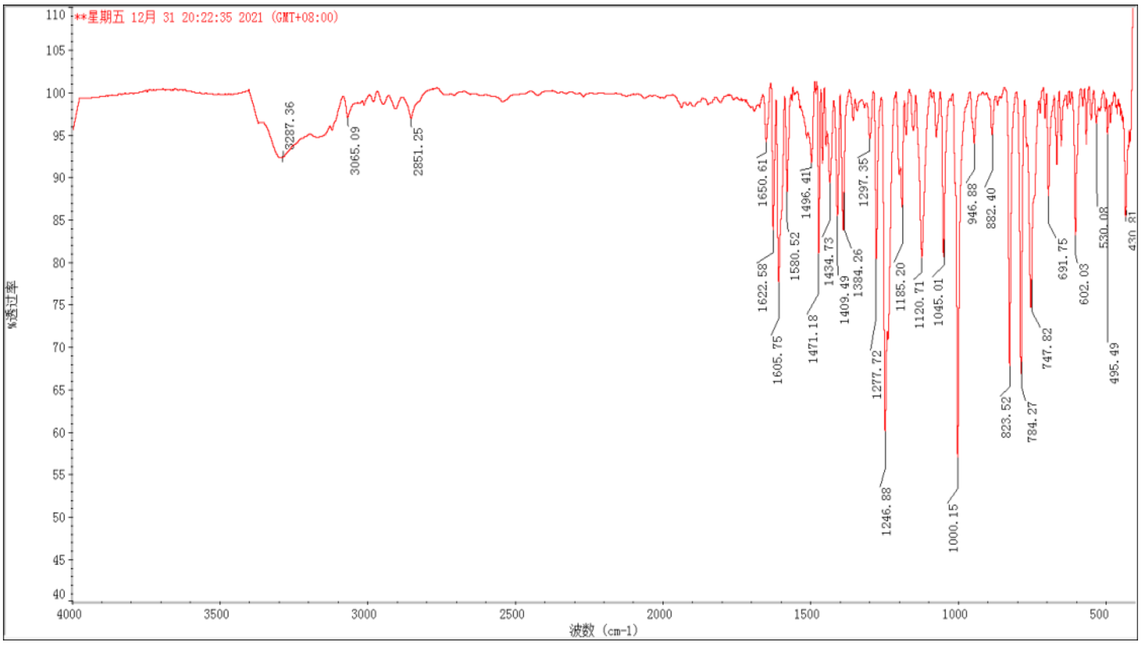

Figure S25. IR spectra of the compound **2g**

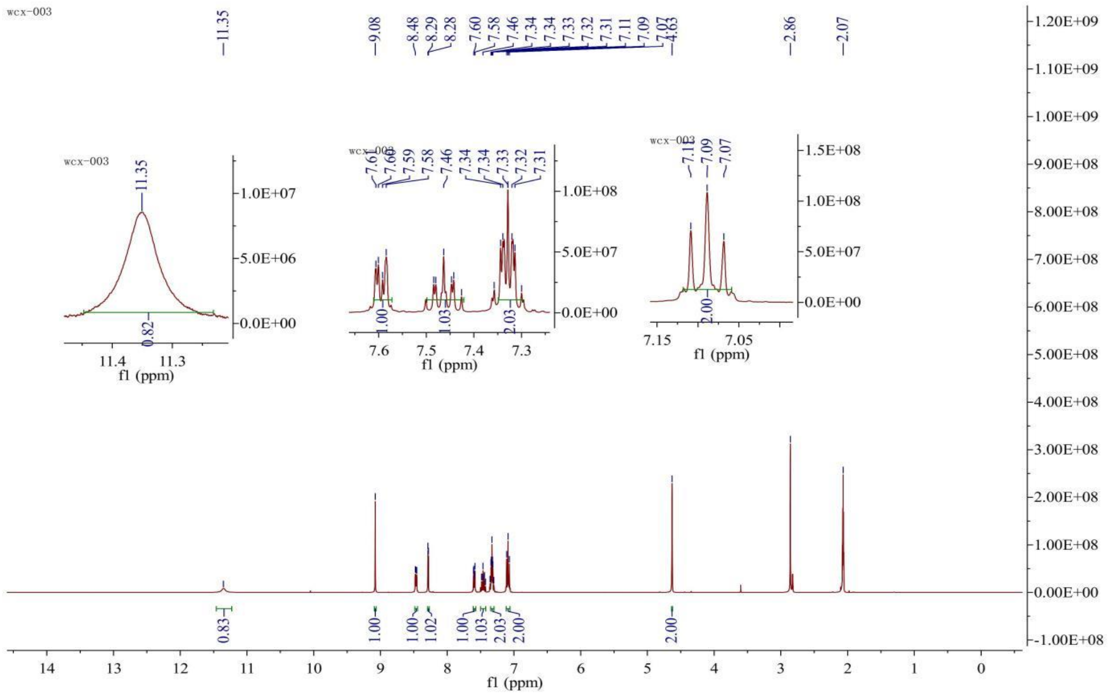

Figure S26. ^{1}H NMR spectra of the compound **2g** (DMSO)

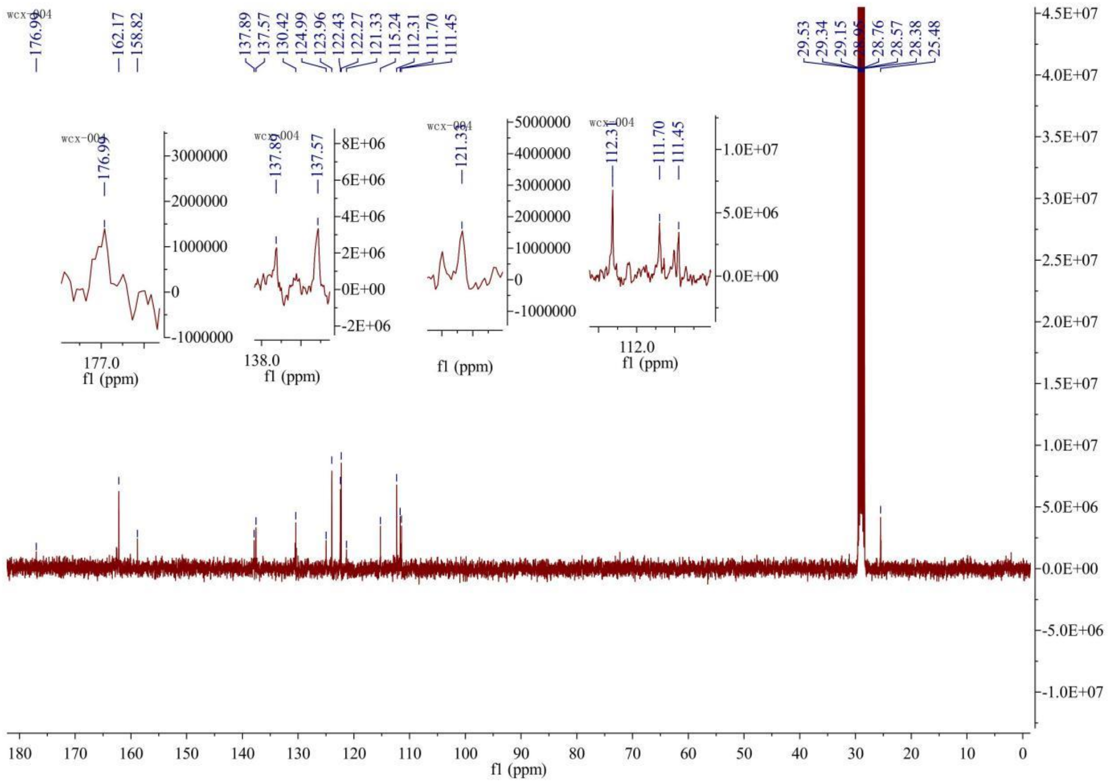

Figure S27. ^{13}C NMR spectra of the compound **2g (**DMSO**)**

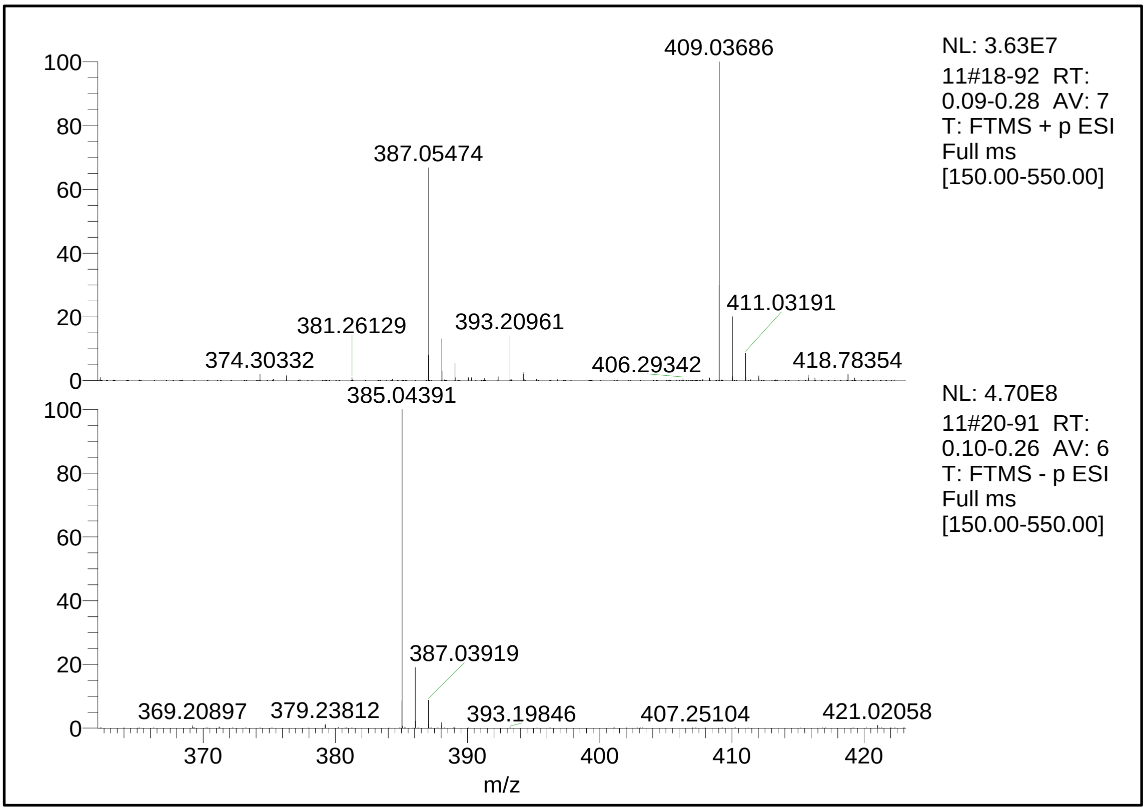

Figure S28. HR-MS of the compound **2g**

Calcd for $C_{18}H_{12}F_2N_4S_2$ $[M+Na]^+$: 409.0369; found 409.0369.

8.The IR, ^{1}H NMR, ^{13}C NMR and HRMS of the compound 2h

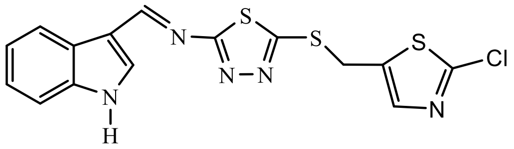

$C_{15}H_{10}ClN_5S_3$, yellow solid powder；m.p.167.6-169.2 ℃.

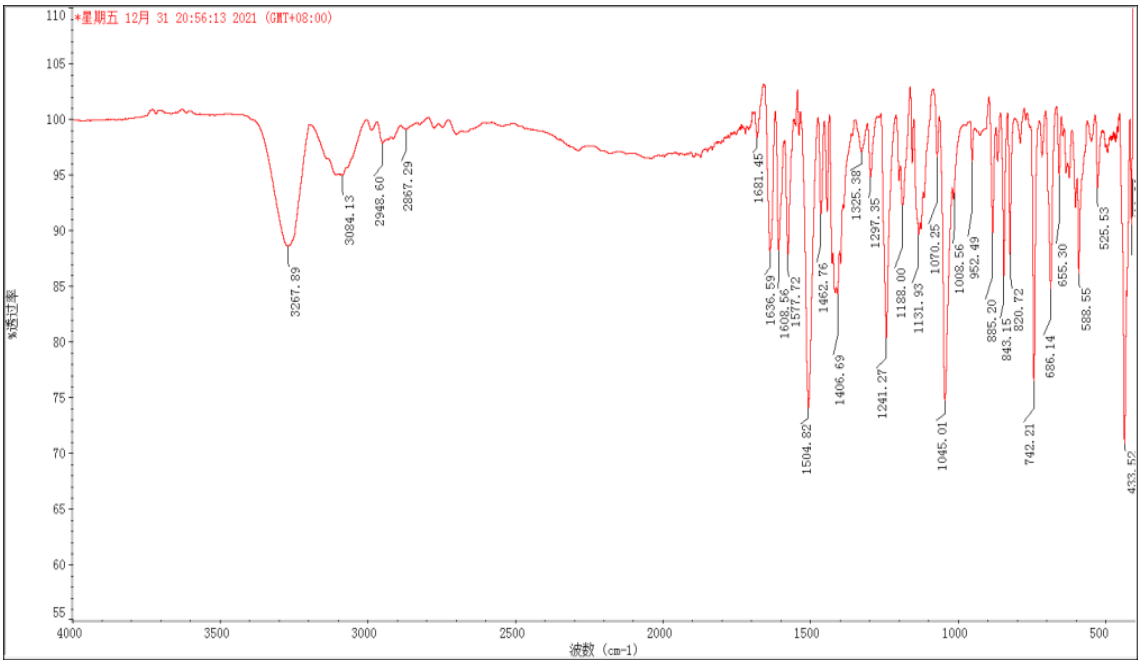

Figure S29. IR spectra of the compound **2h**

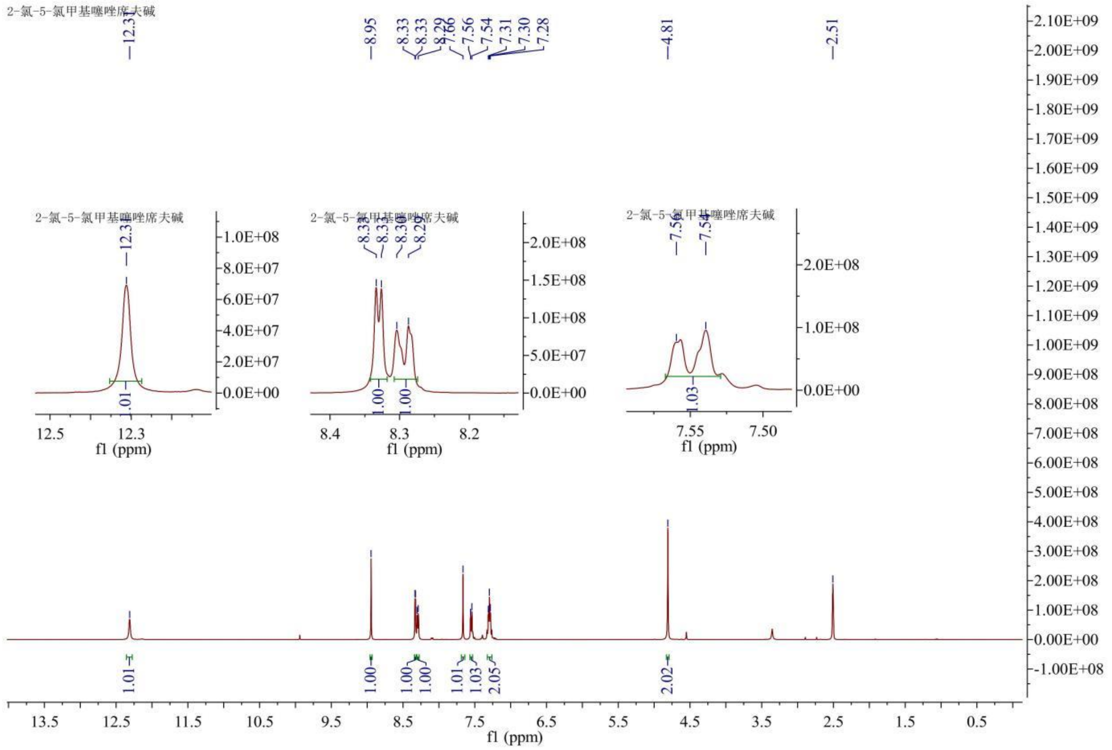

Figure S30. ^{1}H NMR spectra of the compound **2h** (DMSO)

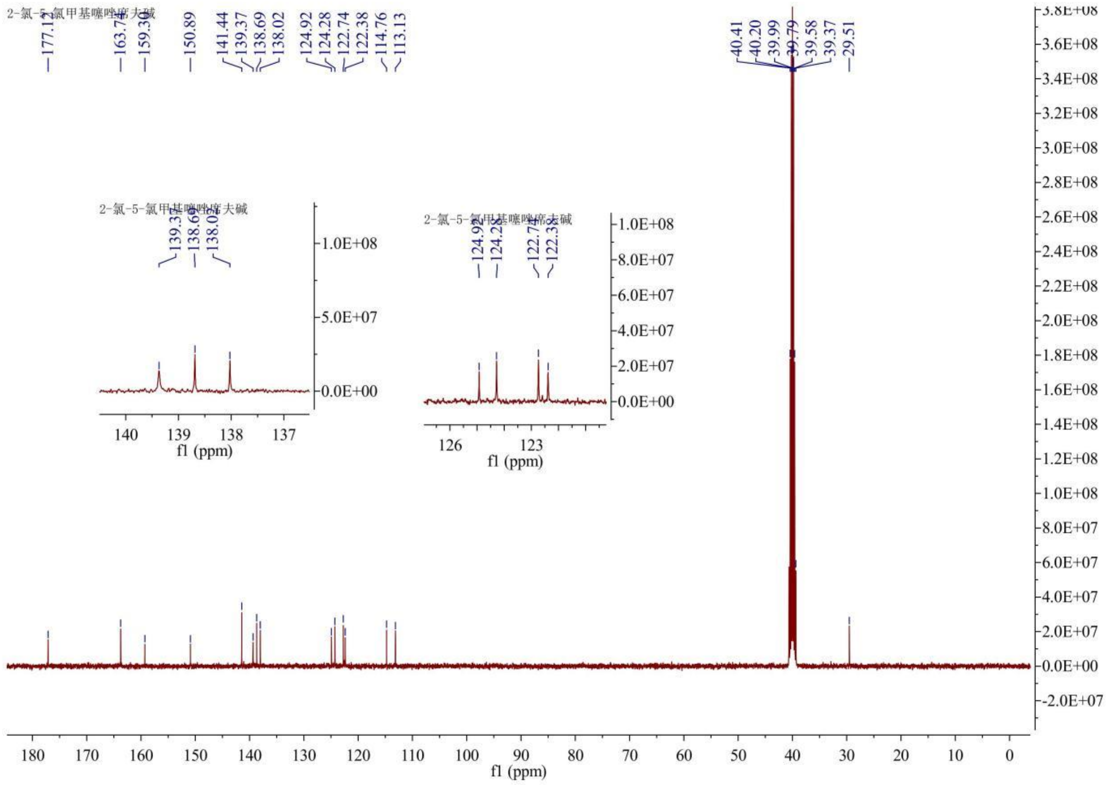

Figure S31. ^{13}C NMR spectra of the compound **2h** (DMSO)

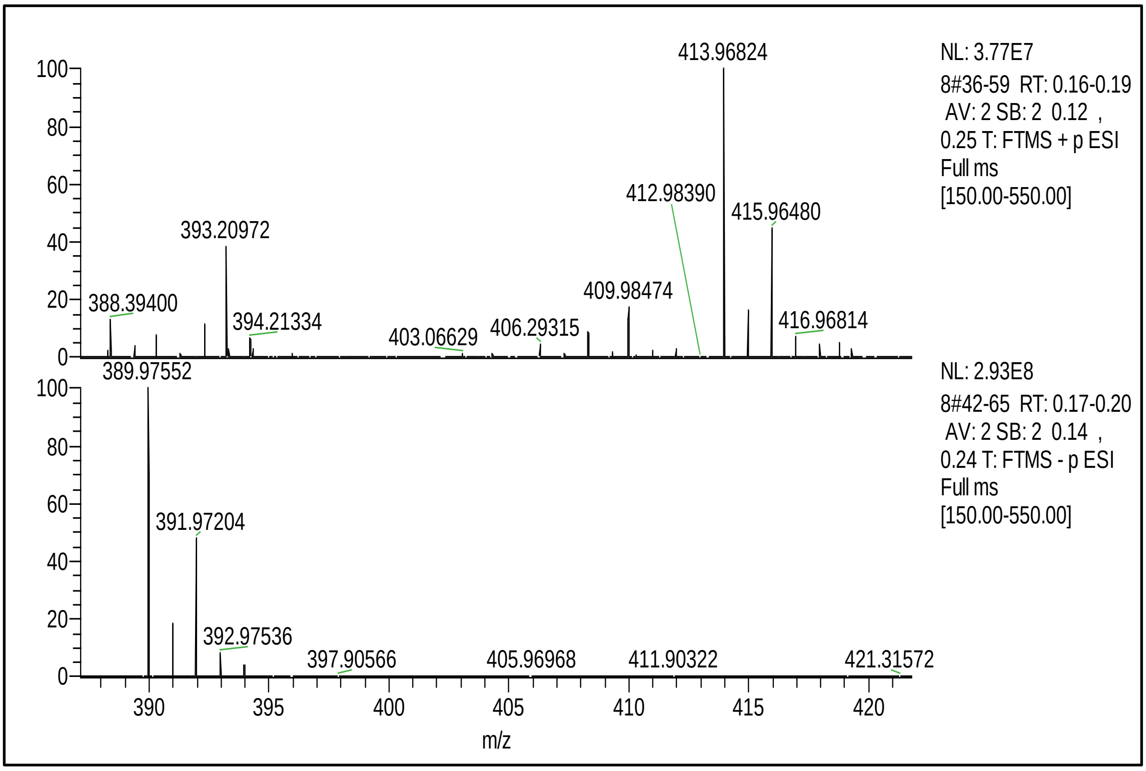

Figure S32. HR-MS of the compound **2h**

Calcd for $C_{15}H_{10}ClN_5S_3$ $[M+Na]^+$: 413.9685; found 413.9682.

9.The IR, ¹H NMR, ¹³C NMR and HRMS of the compound **2i**

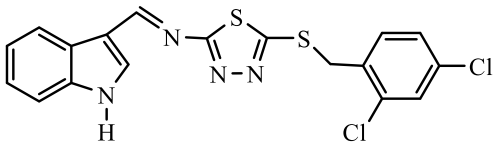

$C_{18}H_{12}Cl_2N_4S_2$, yellow solid powder； m. p. 182.9-183.8 ℃.

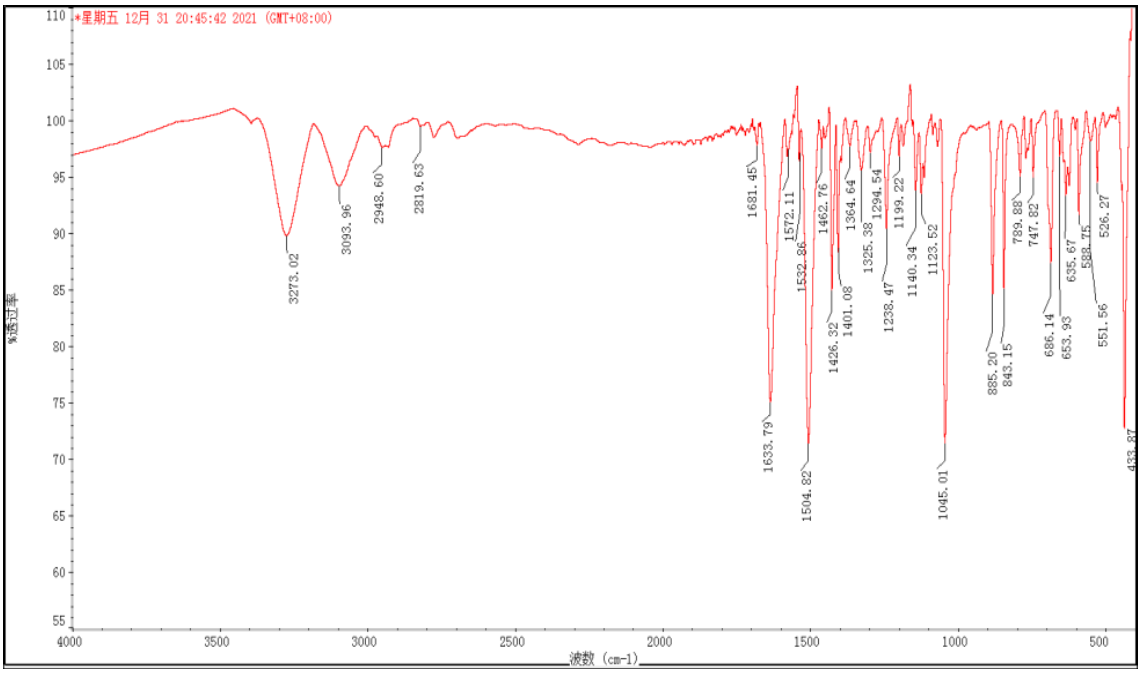

Figure S33. IR spectra of the compound **2i**

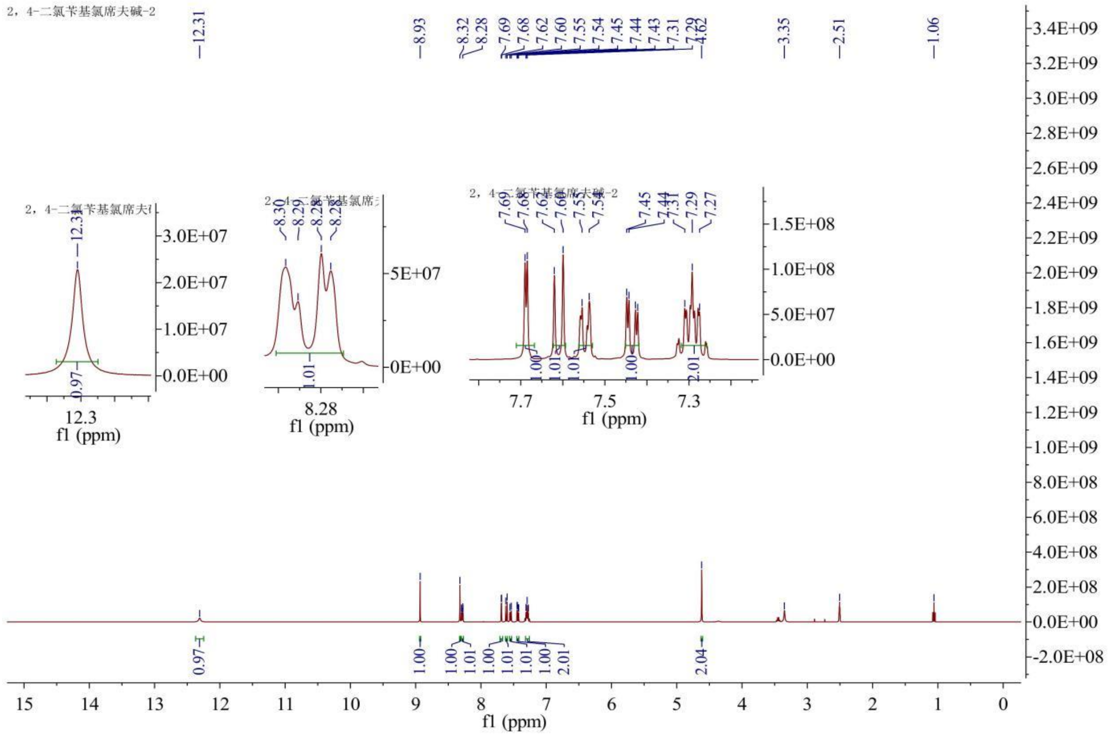

Figure S34. ¹H NMR spectra of the compound **2i** (DMSO)

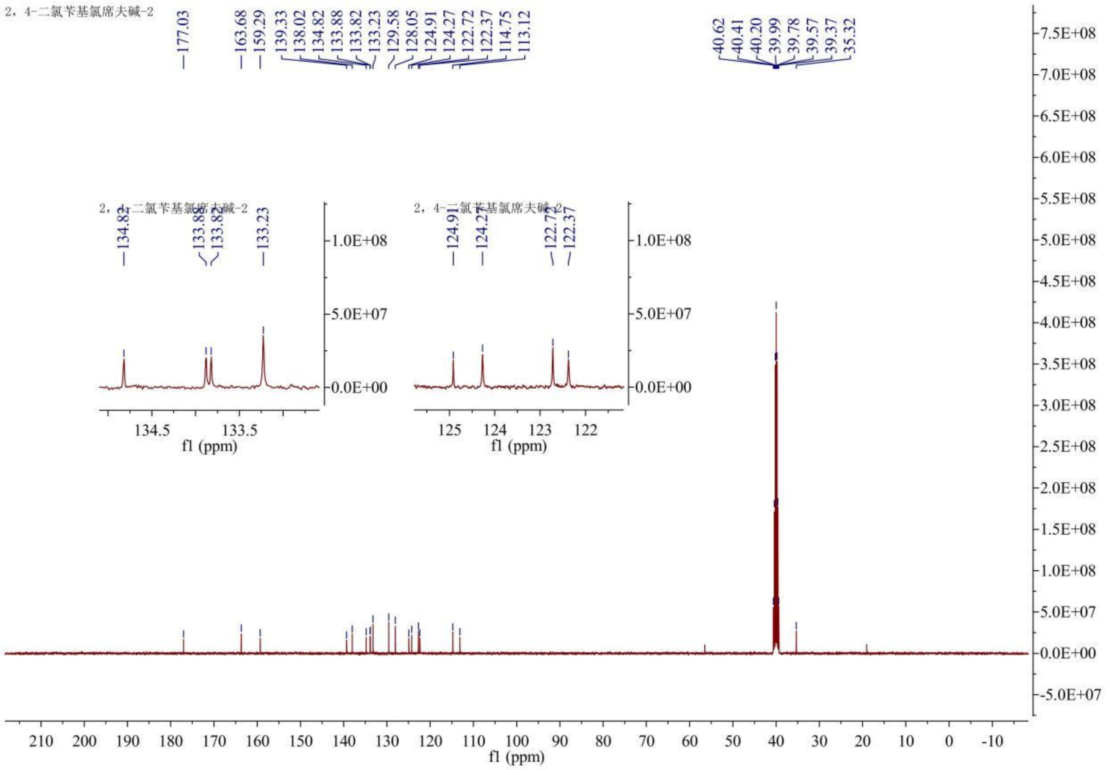

Figure S35. ^{13}C NMR spectra of the compound **2i (**DMSO**)**

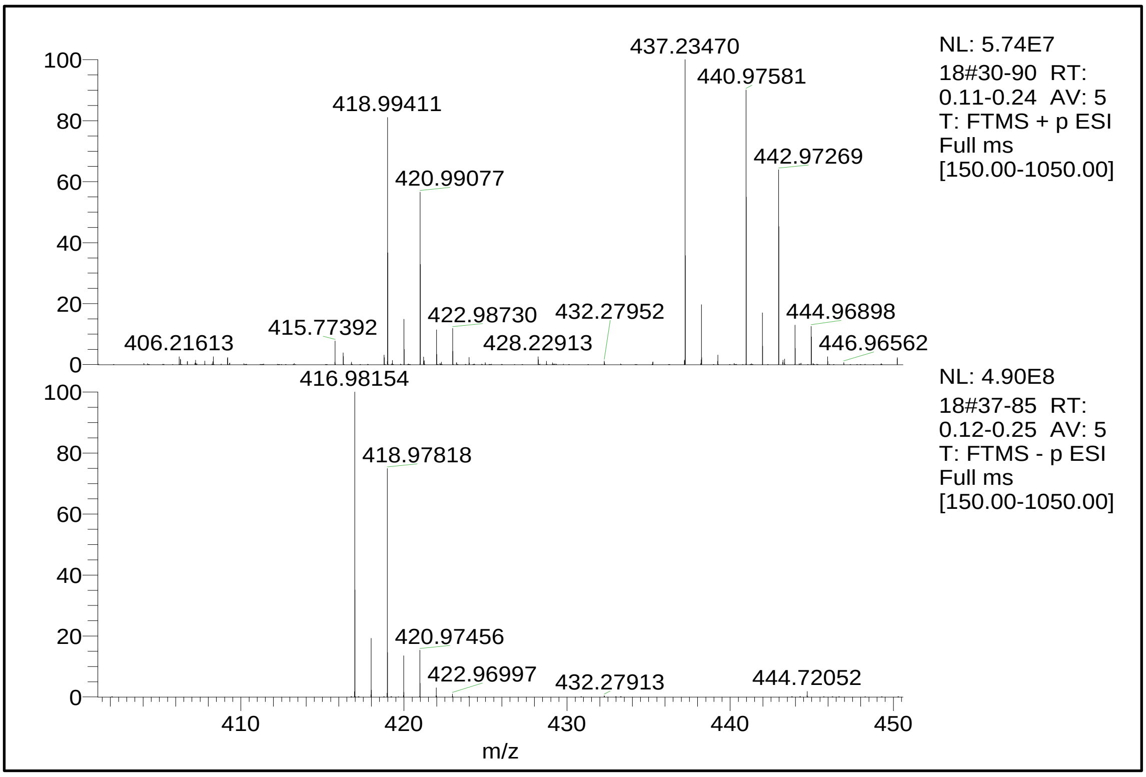

Figure S36. HRMS of the compound **2i**

Calcd for $C_{18}H_{11}Cl_2N_4S_2$ [M-H]$^+$: 416.9802; found 413.9815.

10.The IR, ^{1}H NMR, ^{13}C NMR and HRMS of the compound 2j

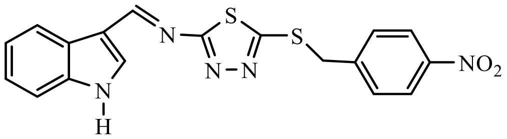

$C_{18}H_{13}N_5O_2S_2$, brown solid powder; m. p. 194.8-195.5 ℃.

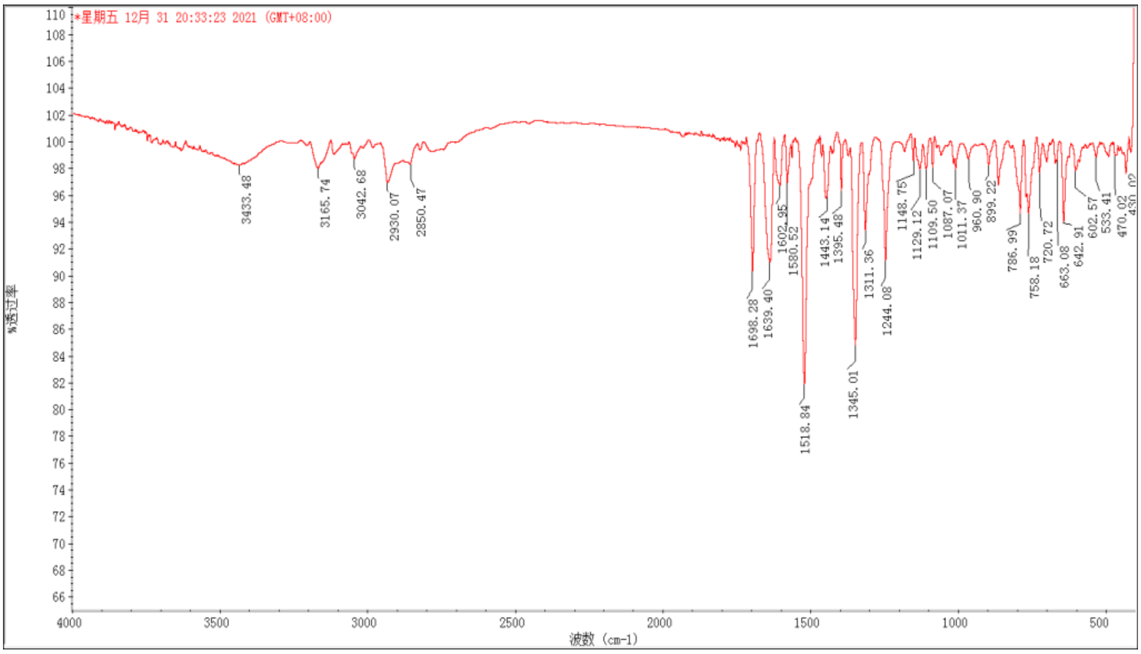

Figure S37. IR spectra of the compound **2j**

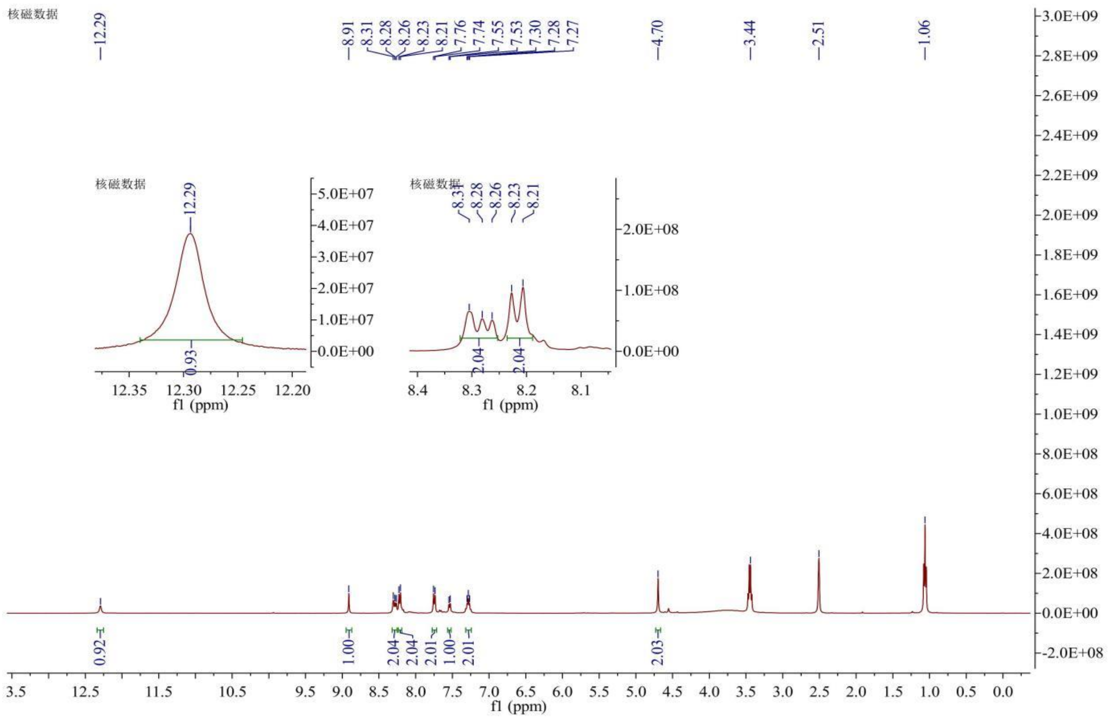

Figure S38. ^{1}H NMR spectra of the compound **2j** **(**DMSO**)**

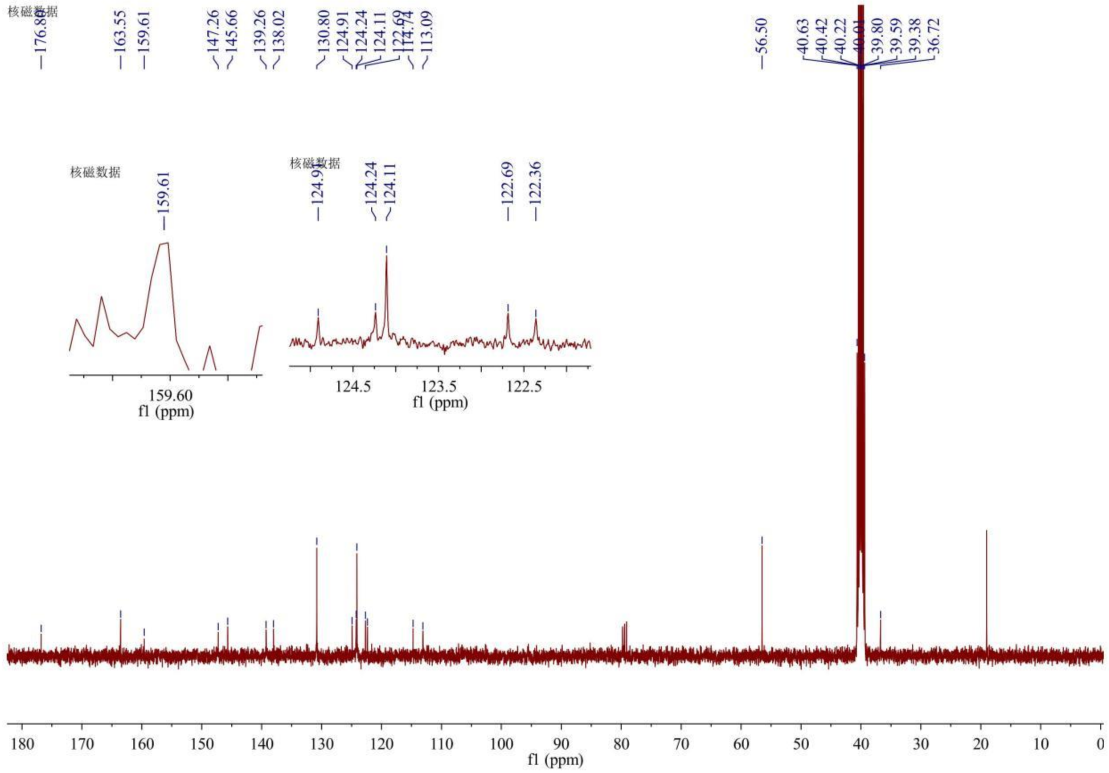

Figure S39. ^{13}C NMR spectra of the compound **2j (**DMSO**)**

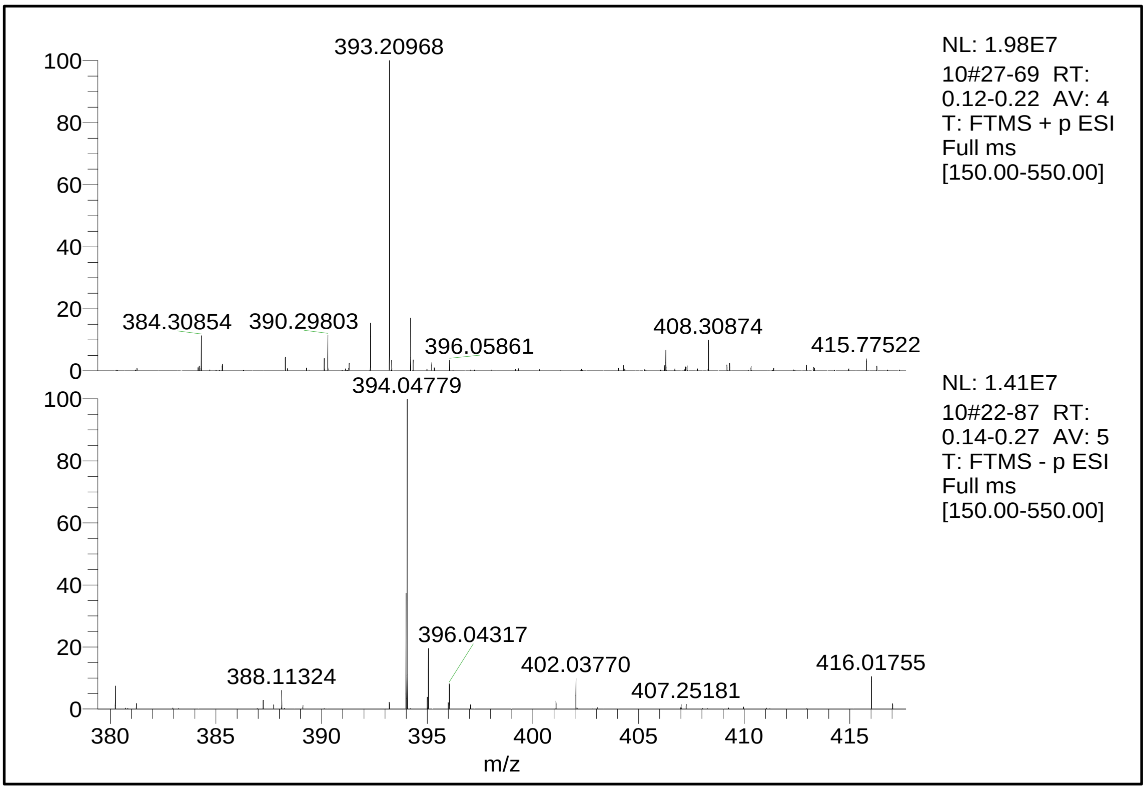

Figure S40. HR-MS of the compound **2j**

Calcd for $C_{18}H_{12}N_5O_2S_2$ [M-H]$^+$: 394.0472; found 394.0478.

11.The IR, ^{1}H NMR, ^{13}C NMR and HRMS of the compound 2k

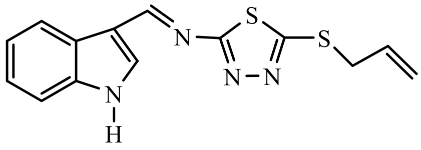

$C_{14}H_{12}N_4S_2$, reddish-brown powder; m. p. 190.3-190.7 ℃.

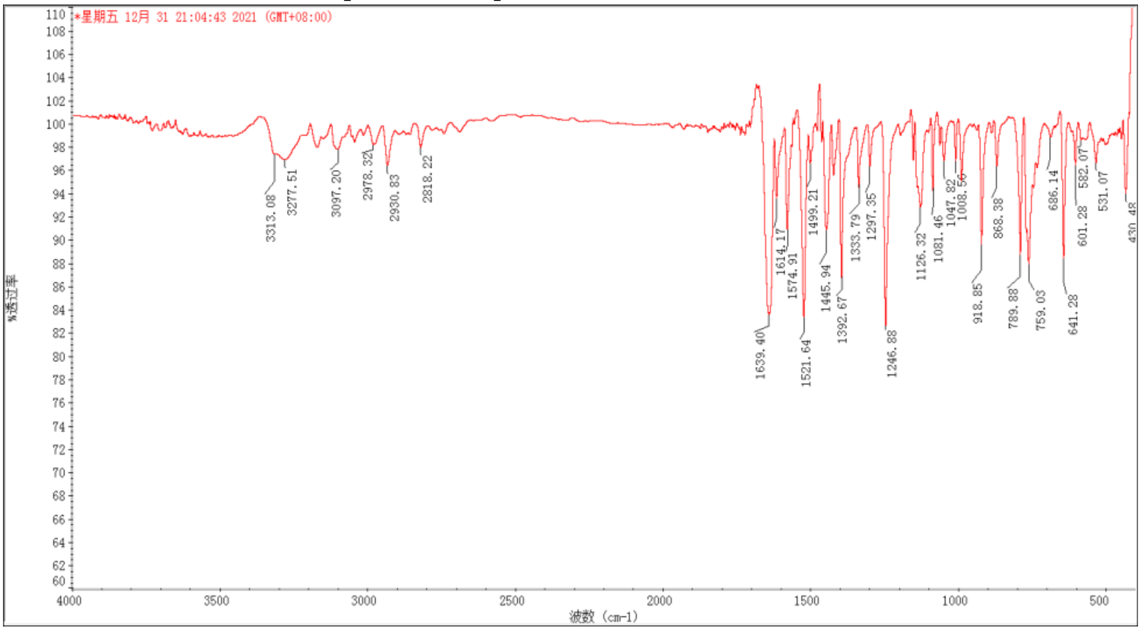

Figure S41. IR spectra of the compound **2k**

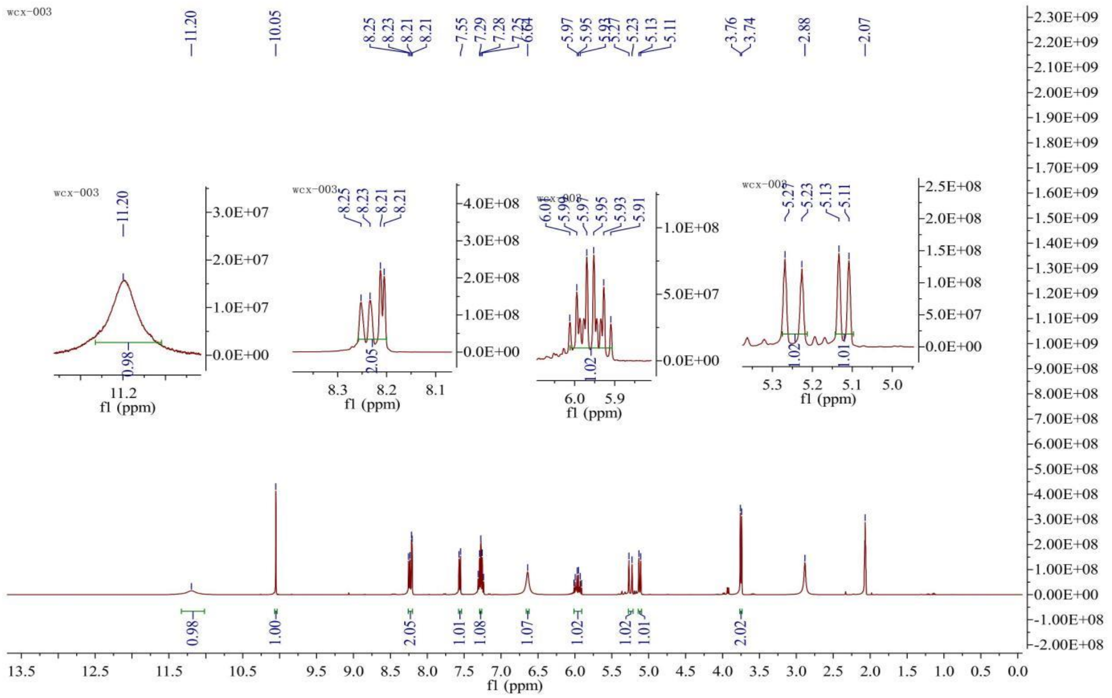

Figure S42. ^{1}H NMR spectra of the compound **2k** **(**Acetone**)**

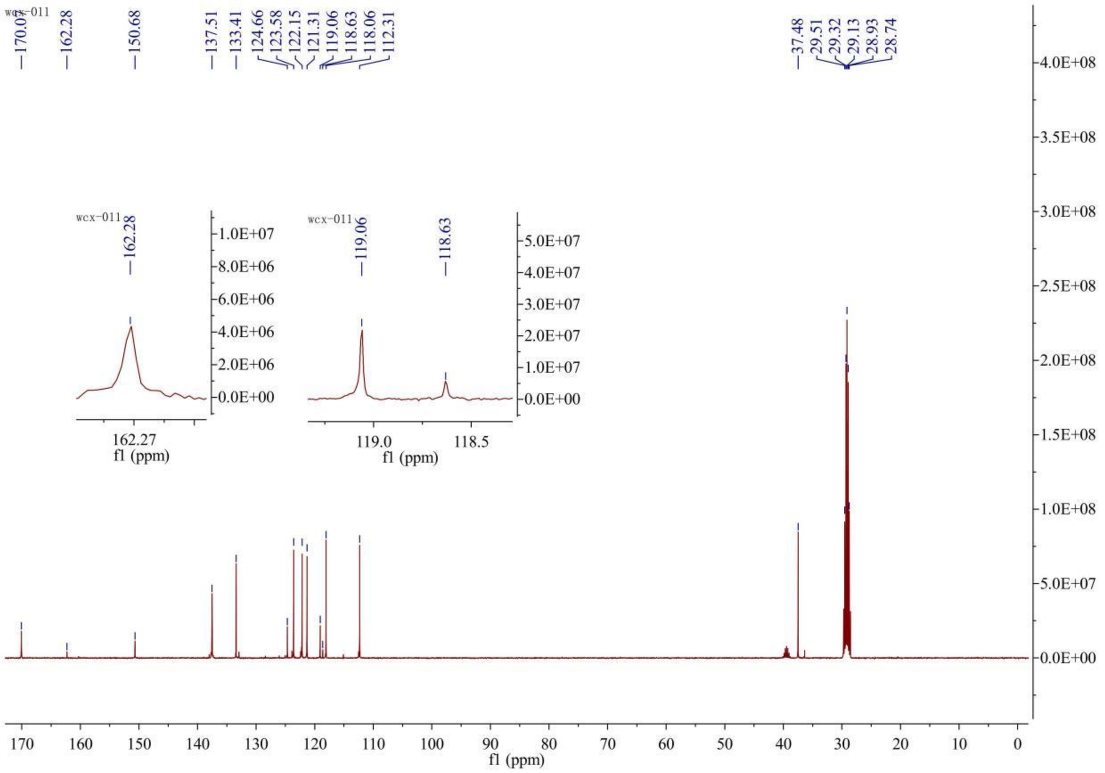

Figure S43. ^{13}C NMR spectra of the compound **2k (**Acetone**)**

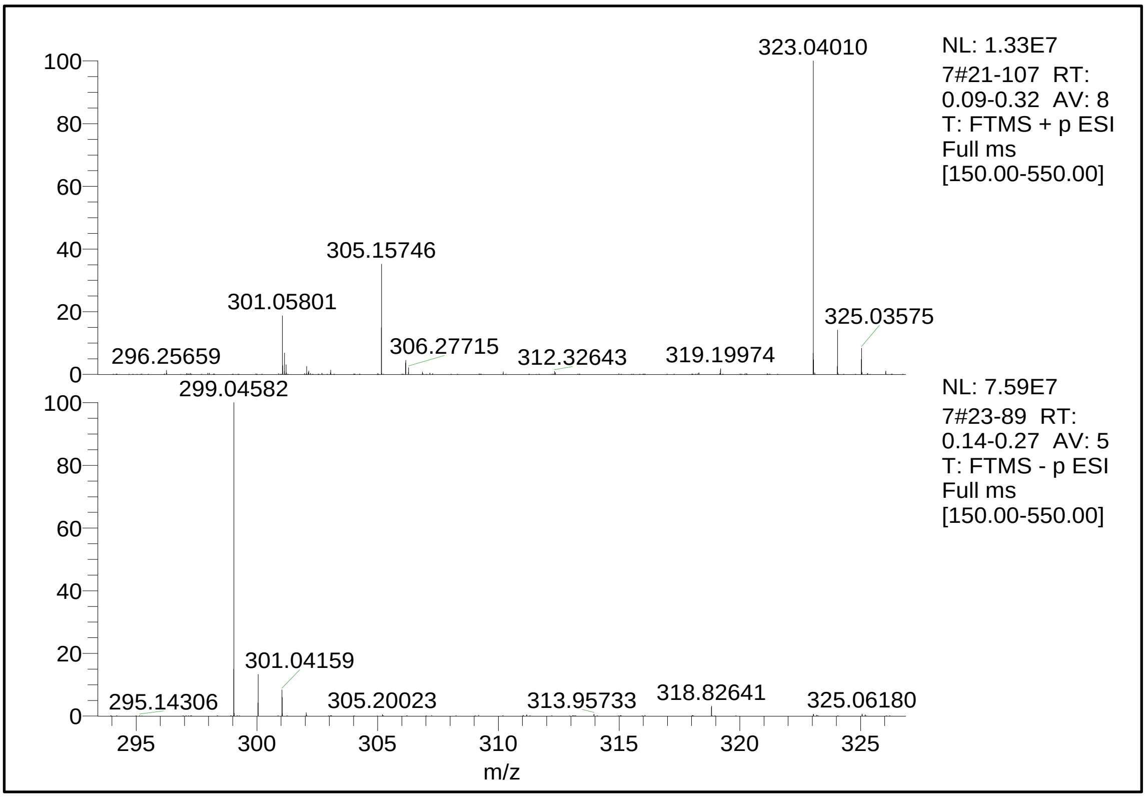

Figure S44. HR-MS of the compound **2k**

Calcd for $C_{14}H_{12}N_4S_2$ $[M+Na]^+$: 323.0401; found 323.0401.

12.The IR, ^{1}H NMR, ^{13}C NMR and HRMS of the compound 2l

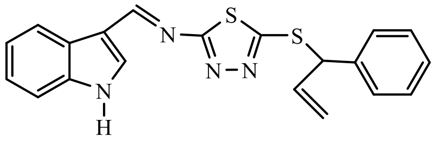

$C_{20}H_{16}N_4S_2$, yellow solid powder; m. p. 217.0-218.3 °C.

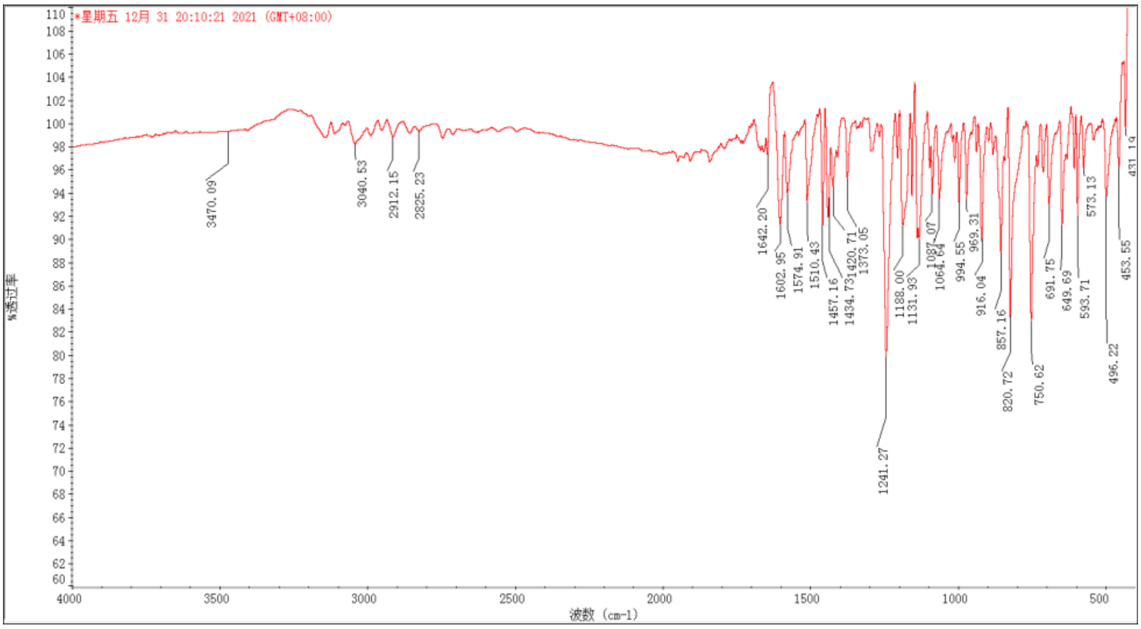

Figure S45. IR spectra of the compound **2l**

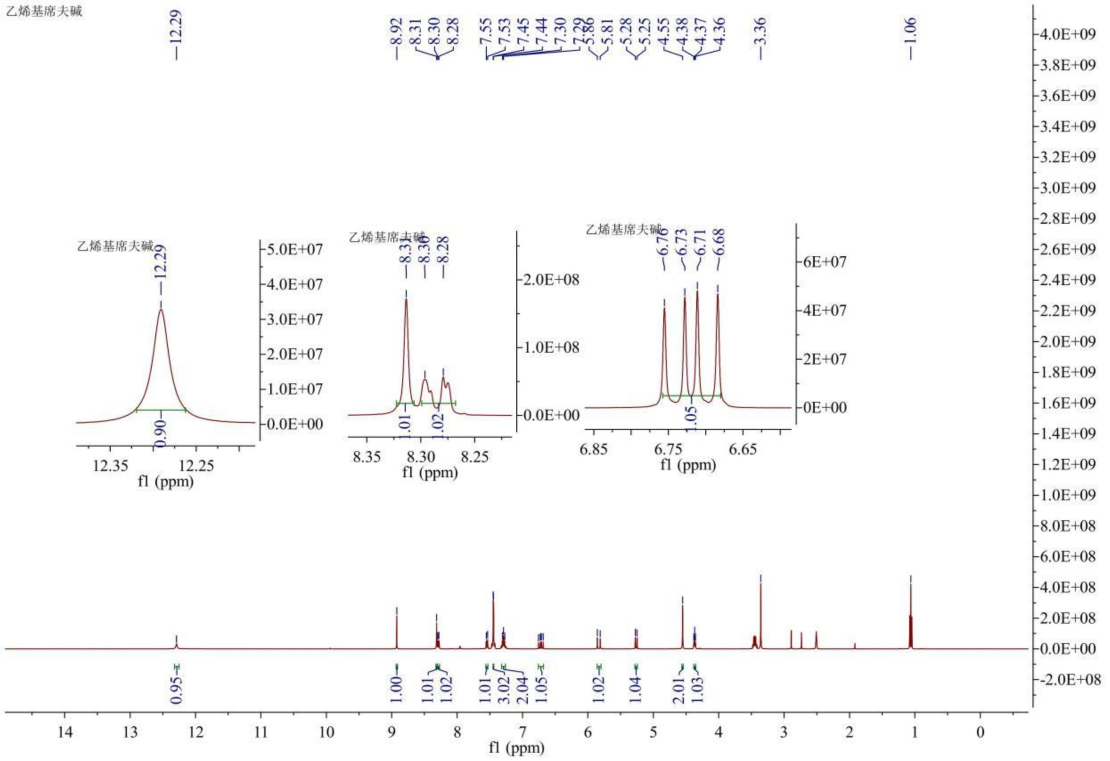

Figure S46. ^{1}H NMR spectra of the compound **2l** (DMSO)

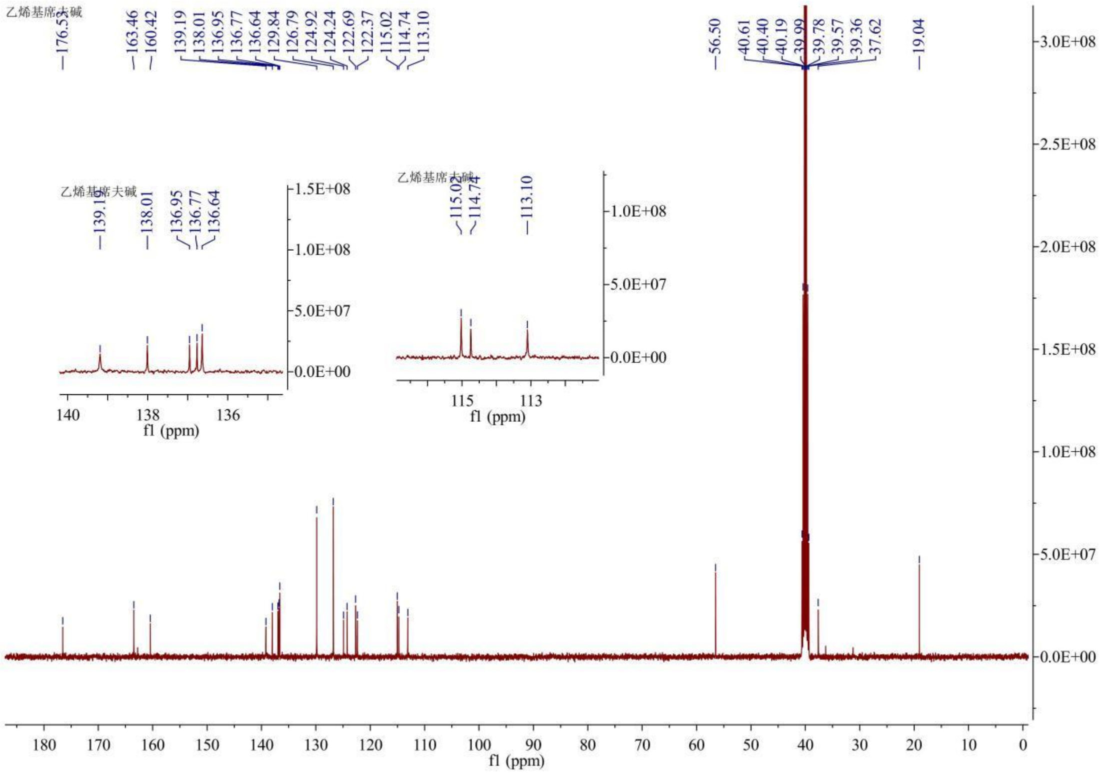

Figure S47. ${}^{13}C$ NMR spectra of the compound **2l** (DMSO)

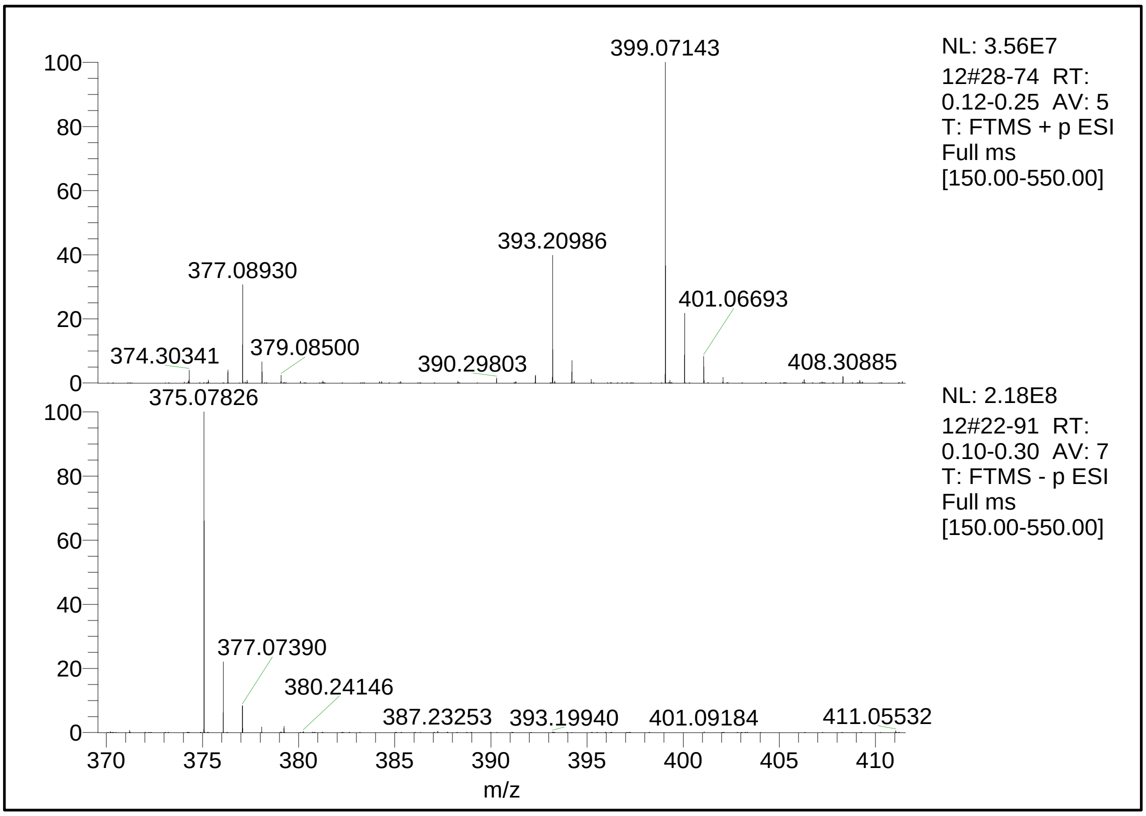

Figure S48. HR-MS of the compound **2l**

Calcd for $C_{20}H_{16}N_4S_2$ $[M+Na]^+$: 399.0714; found 399.0714.

13.The IR, ^{1}H NMR, ^{13}C NMR and HRMS of the compound 2m

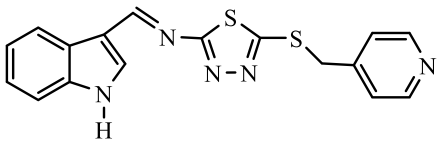

$C_{17}H_{13}N_5S_2$, orange solid powder; m.p.183.2-184.5 °C.

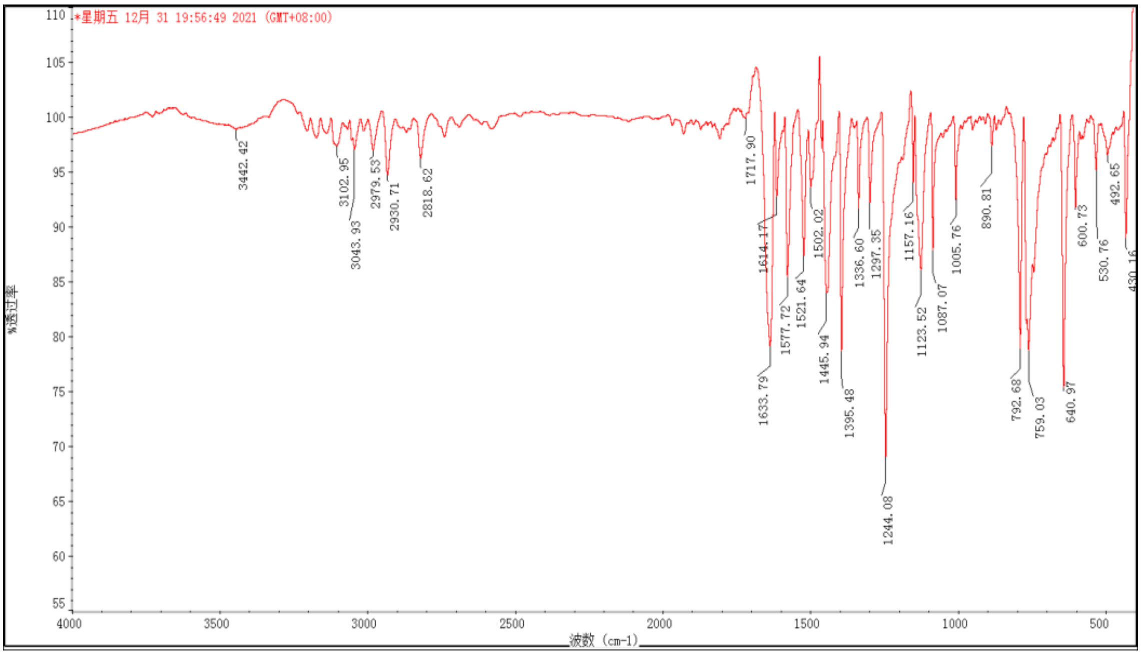

Figure S49. IR spectra of the compound **2m**

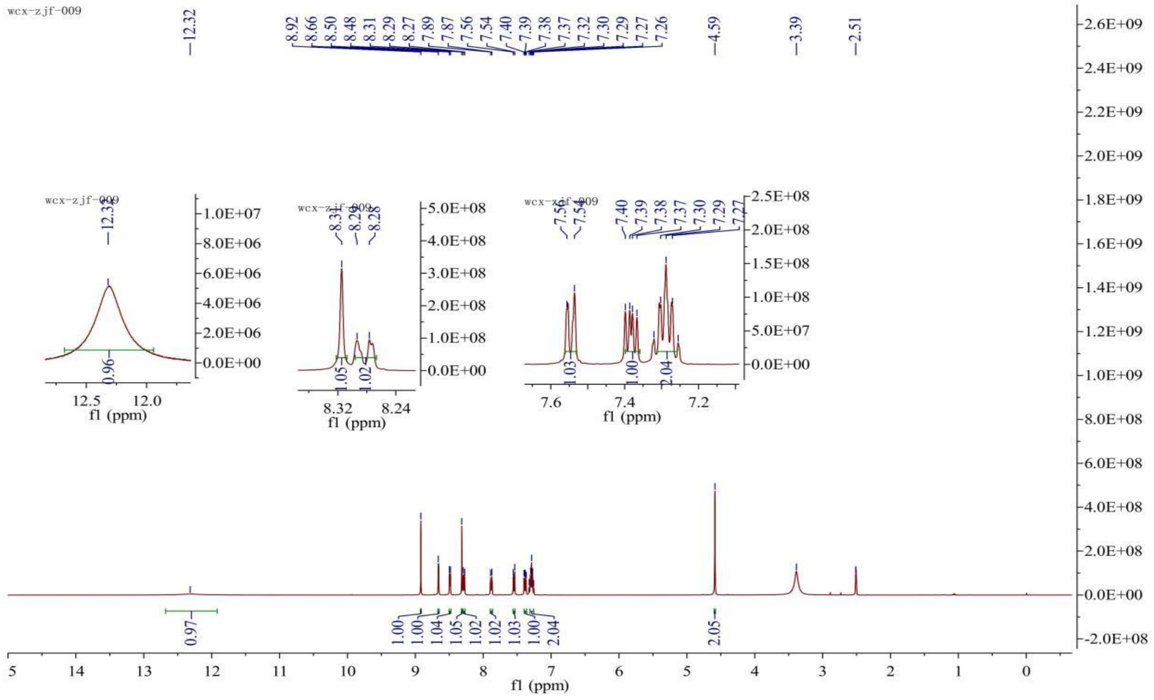

Figure S50. ^{1}H NMR spectra of the compound **2m** (DMSO)

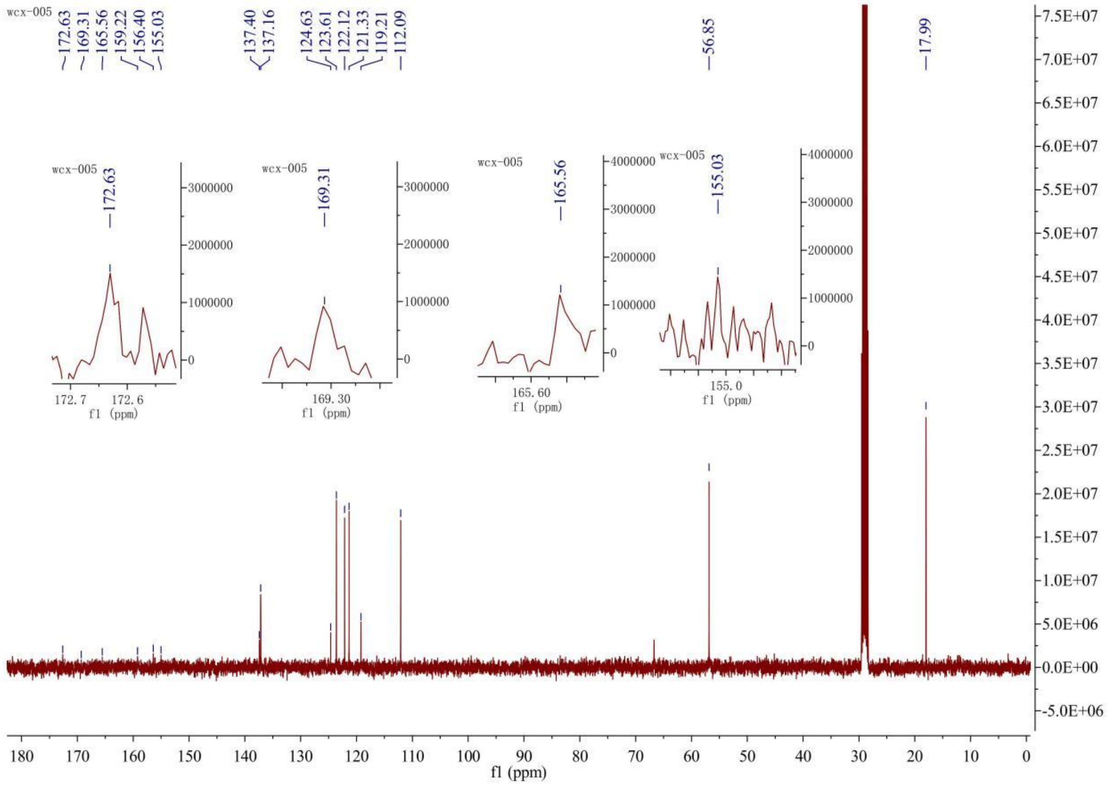

Figure S51. ^{13}C NMR spectra of the compound **2m** (DMSO)

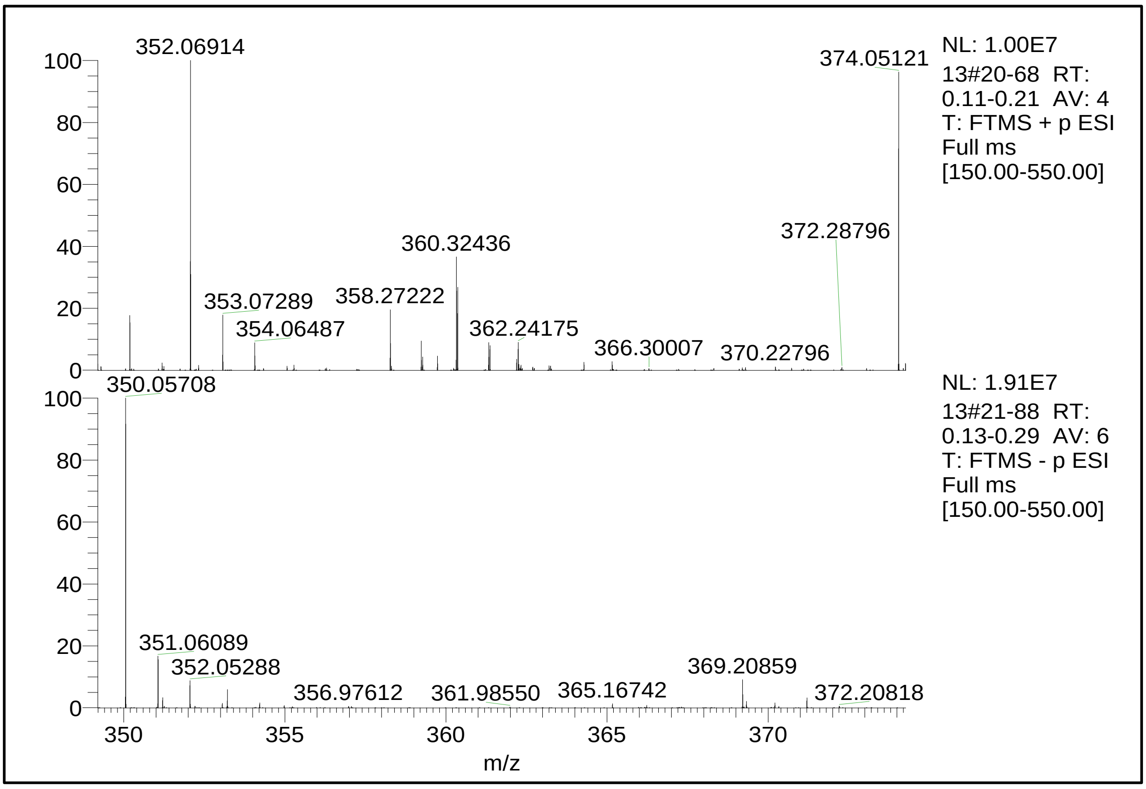

Figure S52. HR-MS of the compound **2m**

Calcd for $C_{17}H_{14}N_5S_2$ [M+H]$^+$: 352.0691; found 352.0691.

14.The IR, ^{1}H NMR, ^{13}C NMR and HRMS of the compound 2n

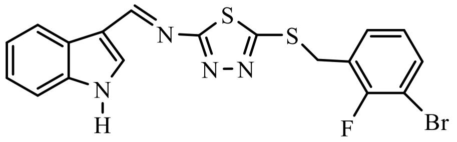

$C_{18}H_{12}BrFN_4S_2$, yellow solid powder; m. p. 198.0-199.3 ℃.

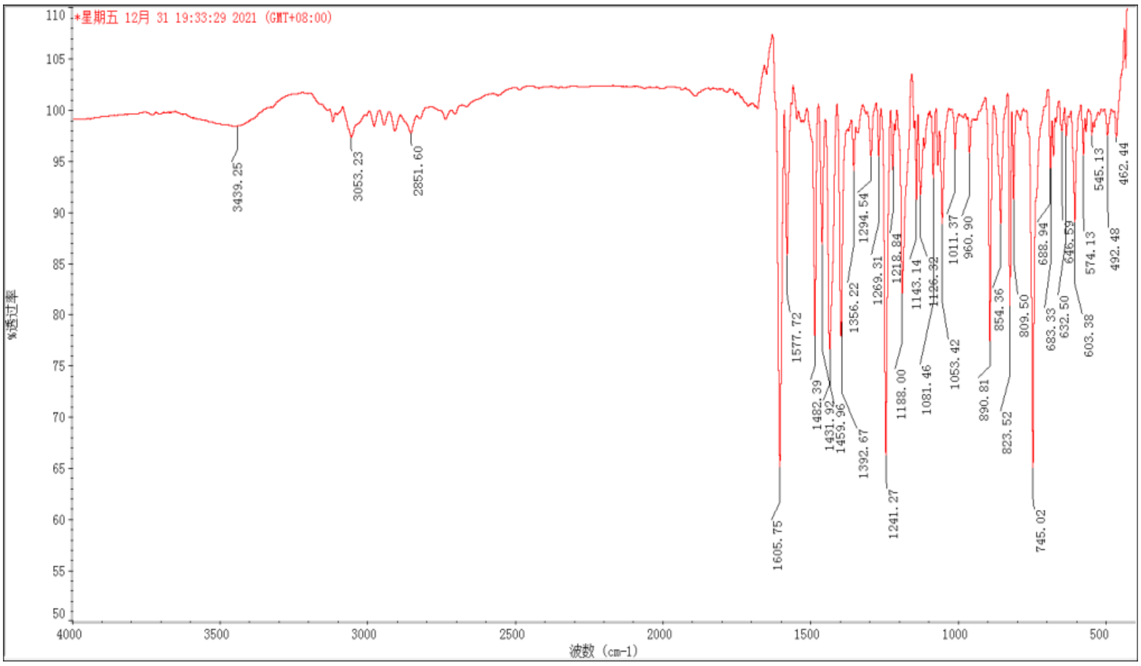

Figure S53. IR spectra of the compound **2n**

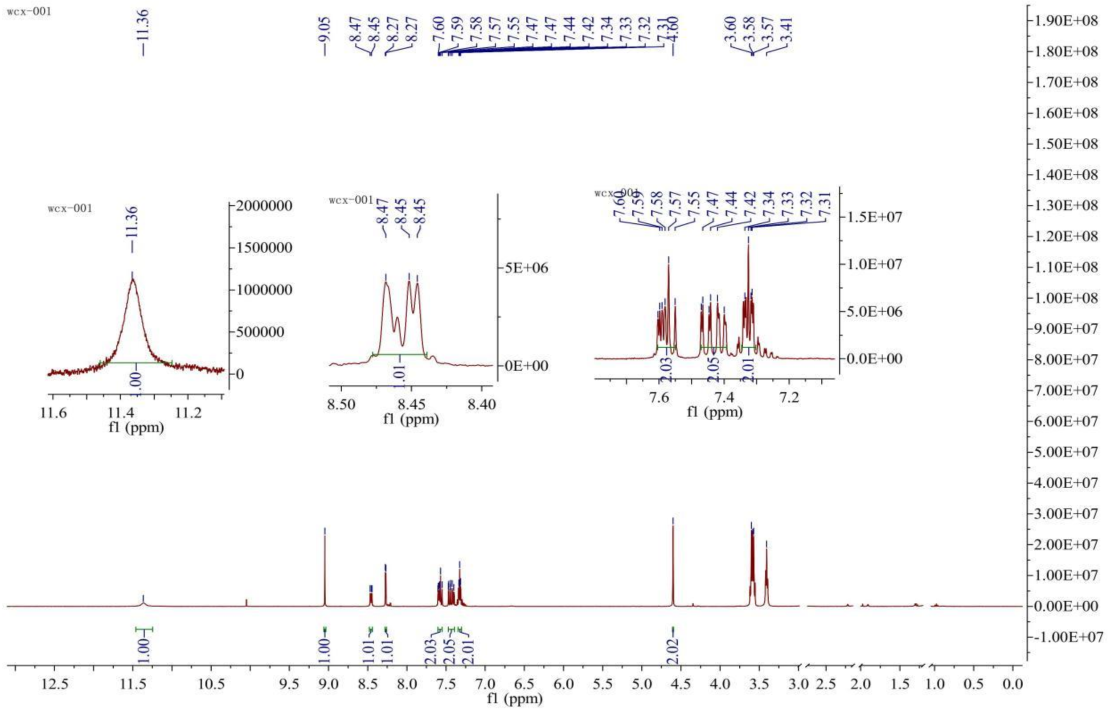

Figure S54. ^{1}H NMR spectra of the compound **2n** (DMSO)

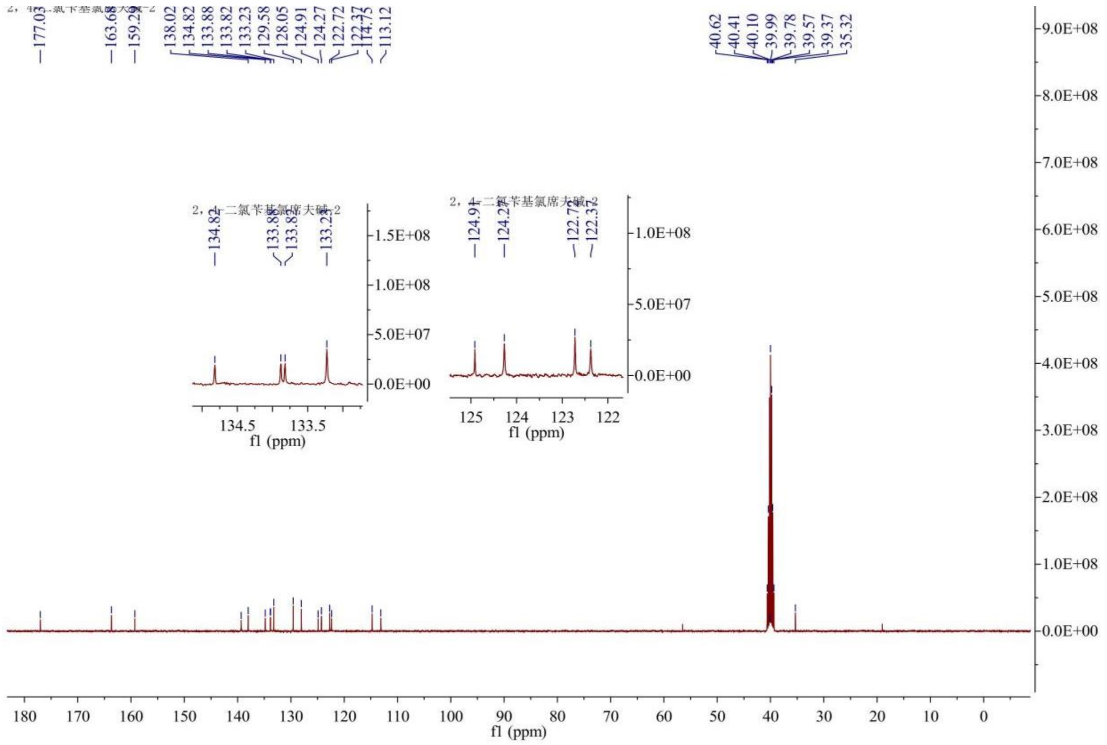

Figure S55. ^{13}C NMR spectra of the compound **2n** (DMSO)

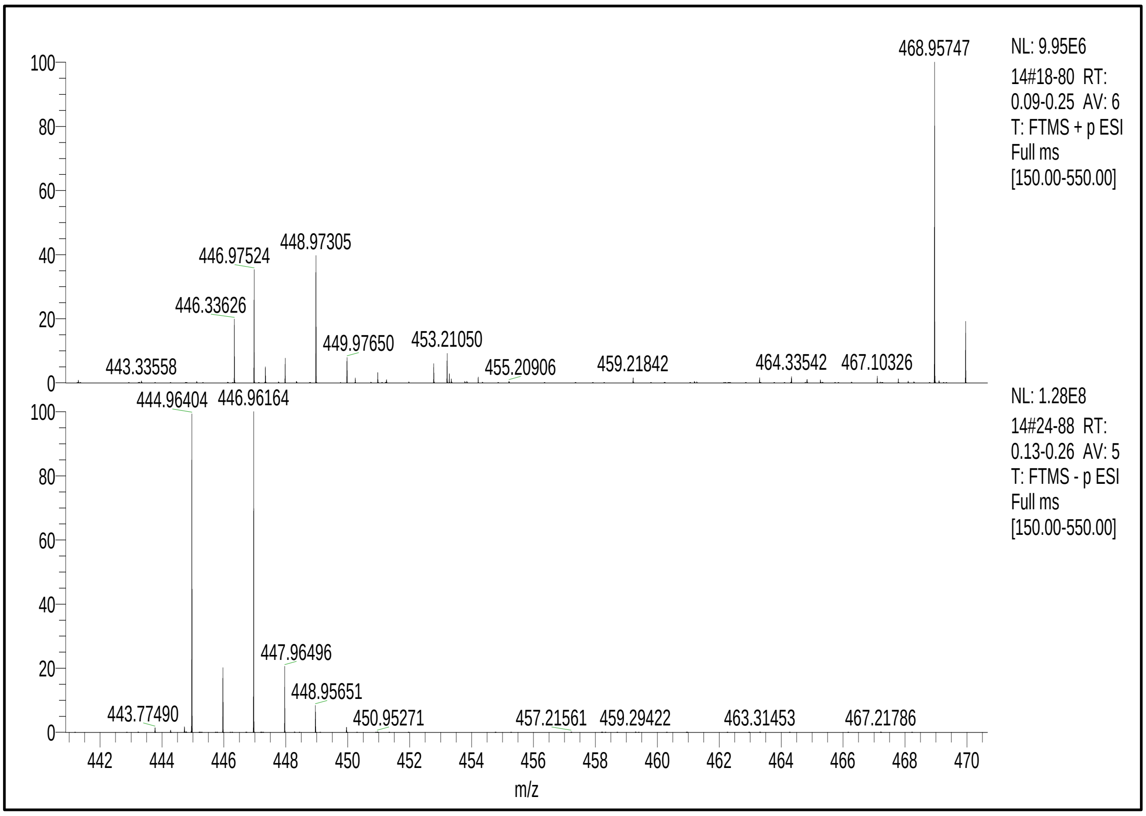

Figure S56. HR-MS of the compound **2n**

Calcd for $C_{18}H_{12}BrFN_4S_2$ $[M+Na]^+$: 468.9569; found 468.9575.

15.The IR, ^{1}H NMR, ^{13}C NMR and HRMS of the compound 2o

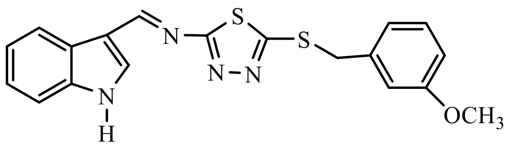

$C_{19}H_{16}N_4OS_2$, yellow solid powder; m. p. 194.2-195.4 °C.

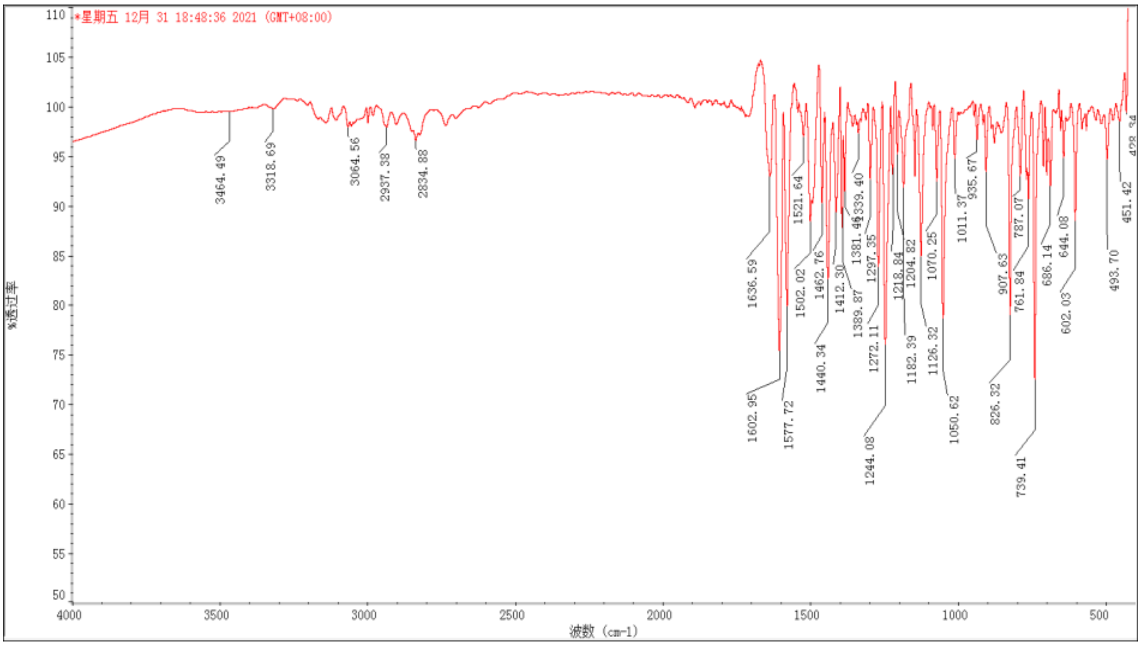

Figure S57. IR spectra of the compound **2o**

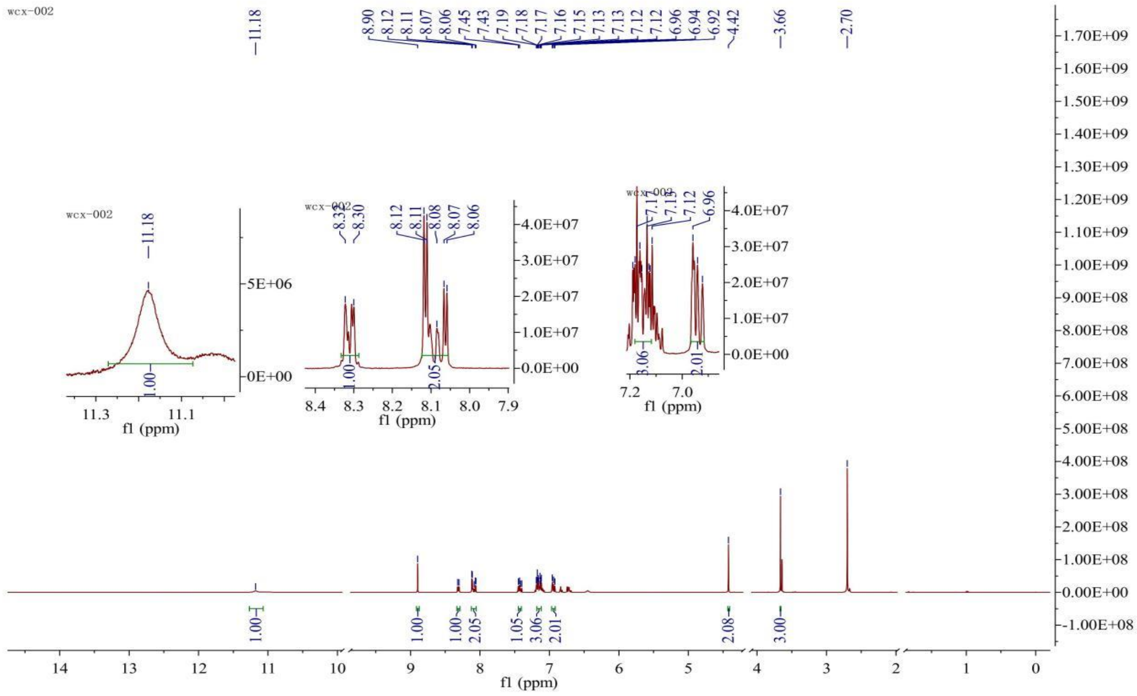

Figure S58. ^{1}H NMR spectra of the compound **2o** (DMSO)

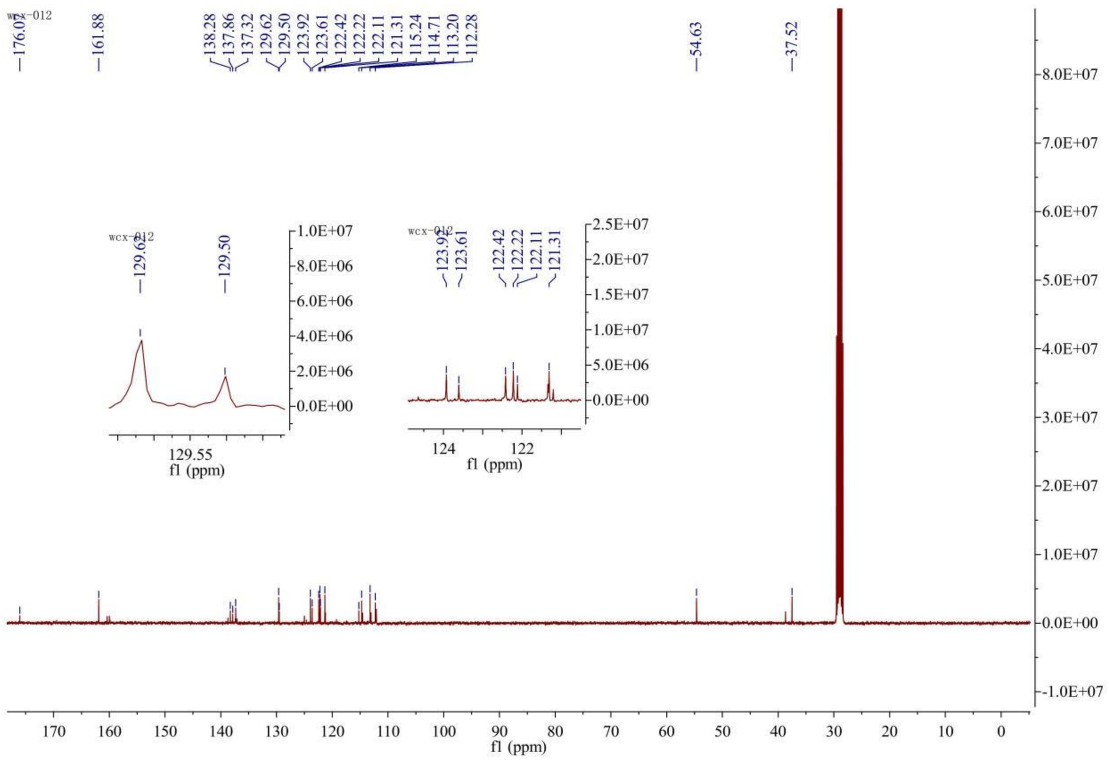

Figure S59. ^{13}C NMR spectra of the compound **2o** (DMSO)

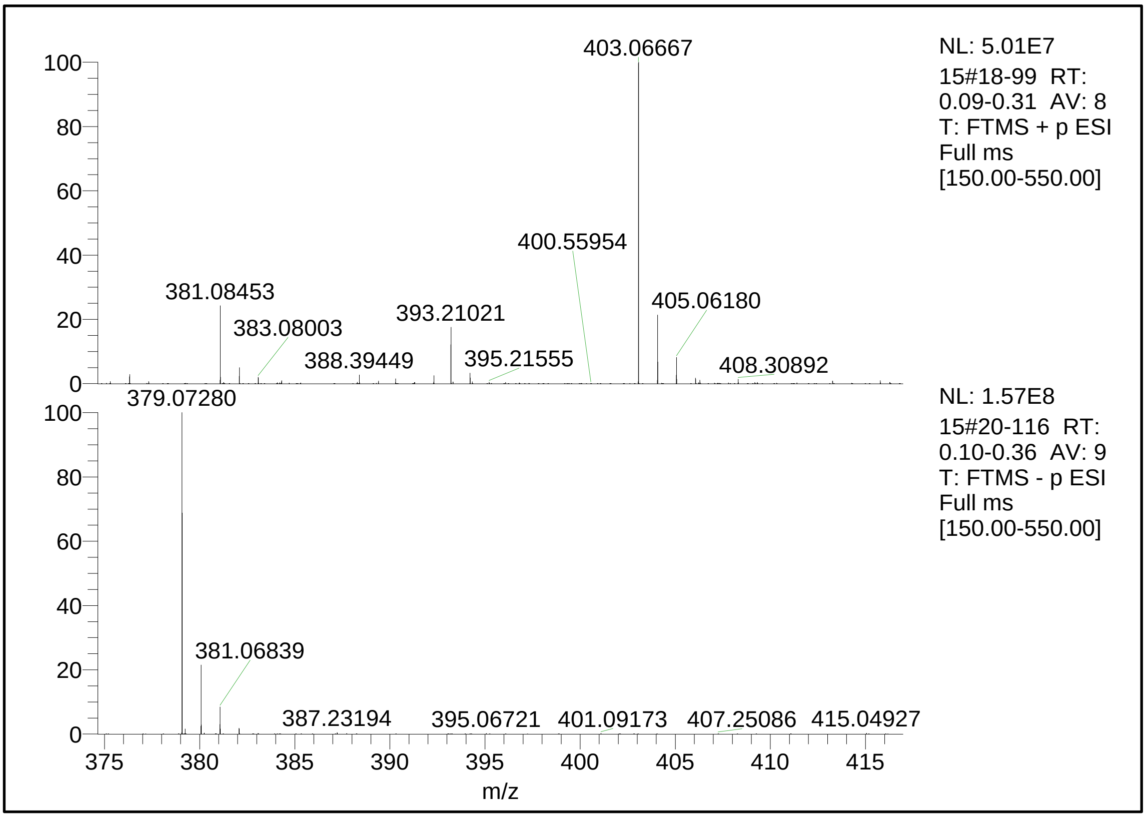

Figure S60. HR-MS of the compound **2o**

Calcd for $C_{19}H_{16}N_4OS_2$ $[M+Na]^+$: 403.0663; found 403.0667.

16.The IR, ¹H NMR, ¹³C NMR and HRMS of the compound 2p

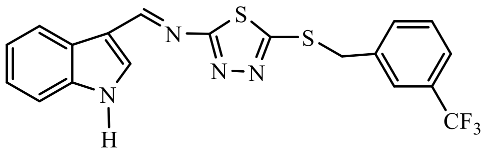

$C_{19}H_{13}F_3N_4S_2$, yellow solid powder；m. p. 201.3-202.2 °C.

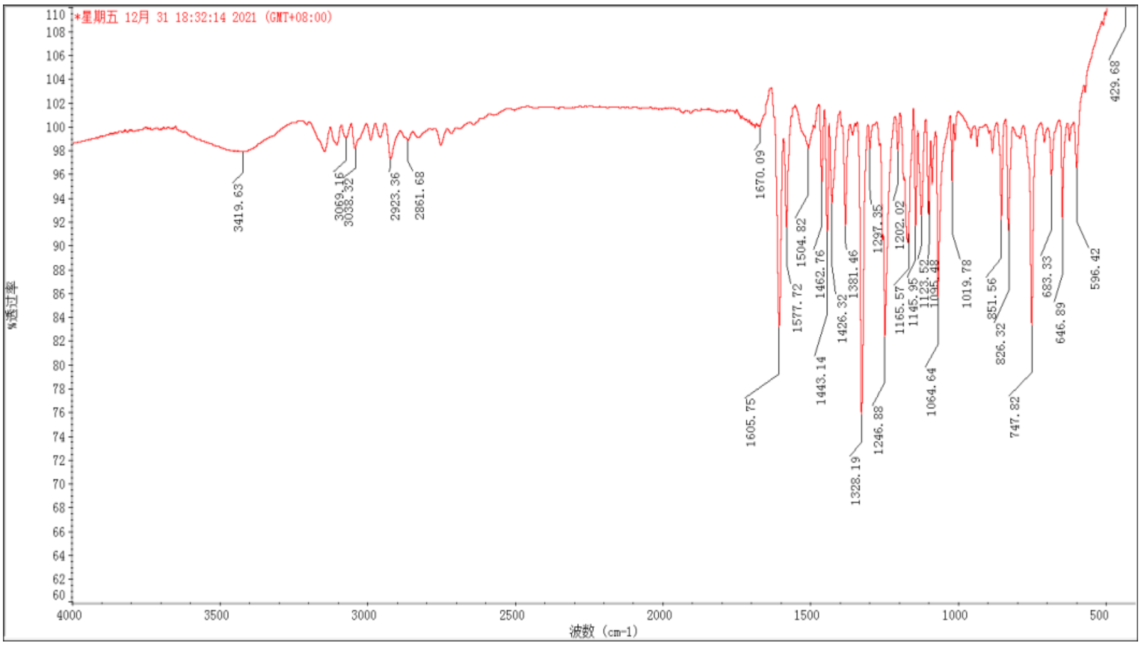

Figure S61. IR spectra of the compound **2p**

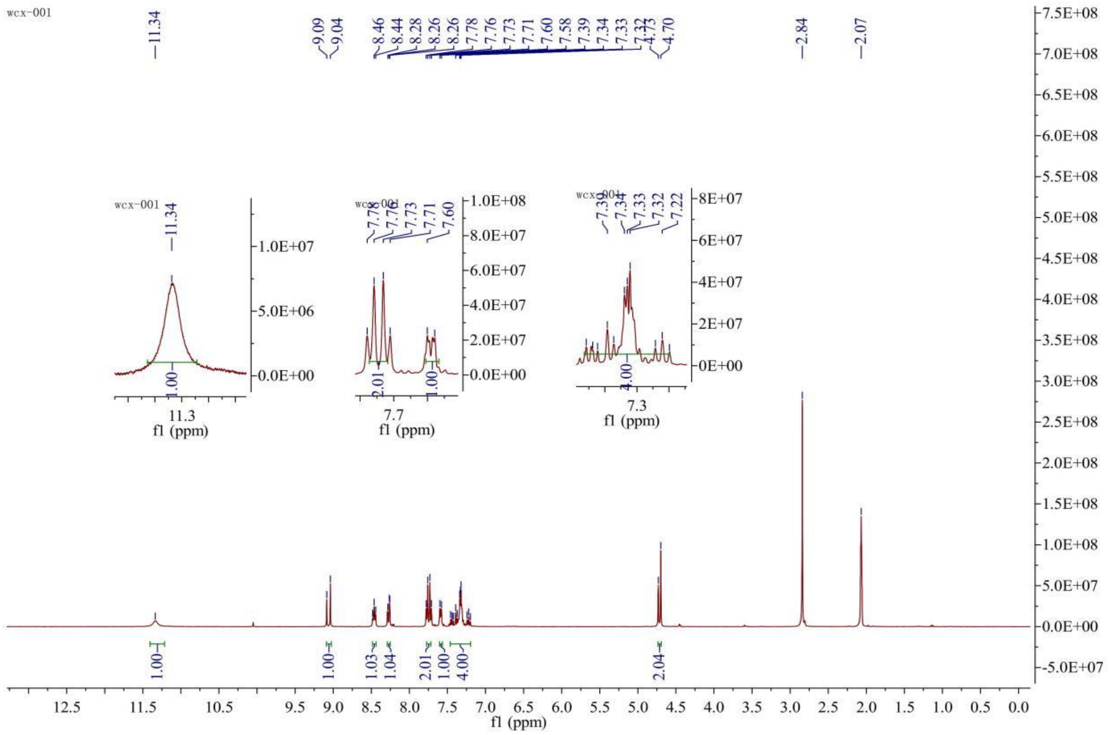

Figure S62. ¹H NMR spectra of the compound **2p** (Acetone)

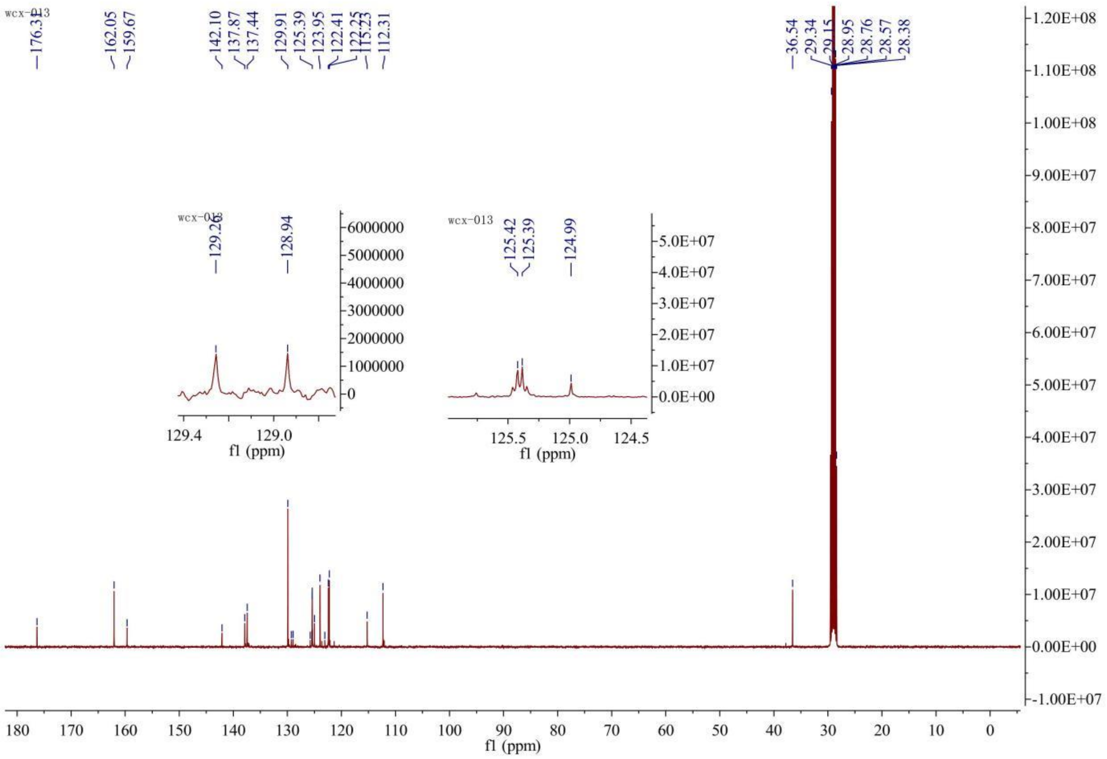

Figure S63. ^{13}C NMR spectra of the compound **2p** (Acetone)

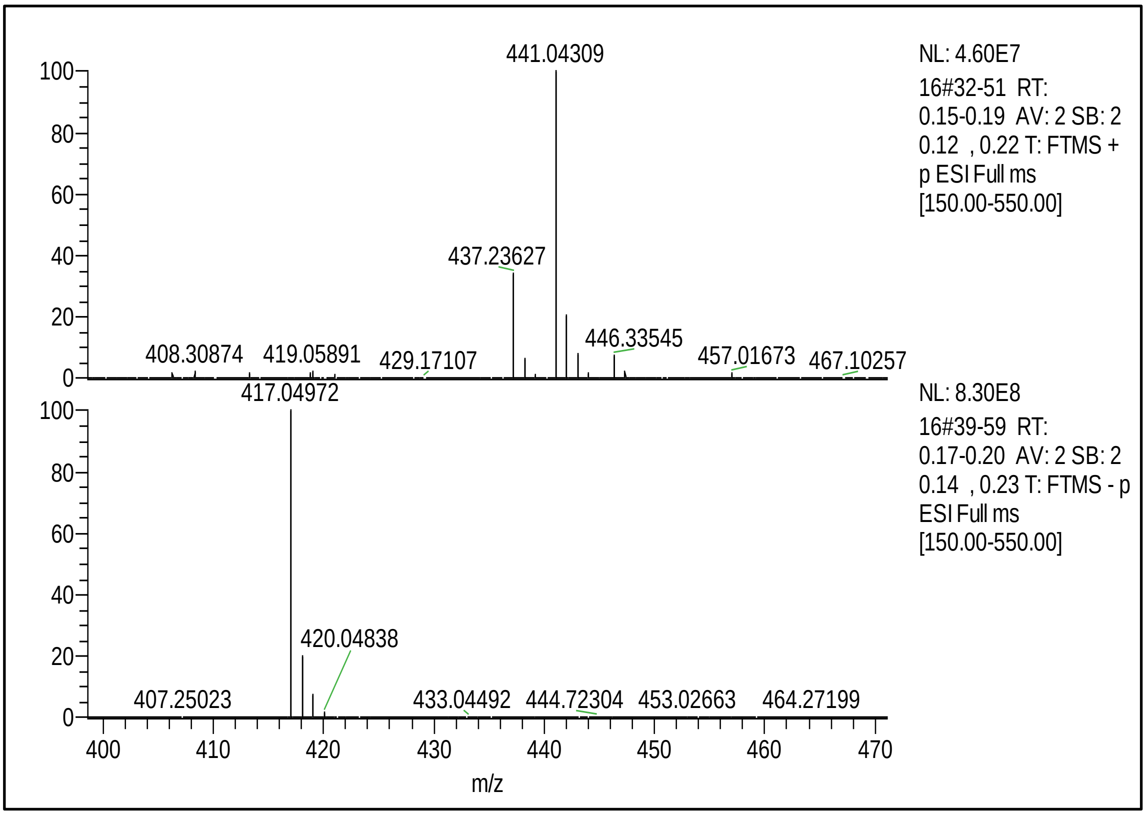

Figure S64. HRMS of the compound **2p**

Calcd for $C_{19}H_{13}F_3N_4S_2$ $[M+Na]^+$: 441.0431; found 441.0431.

17.The IR, ¹H NMR, ¹³C NMR and HRMS of the compound 2q

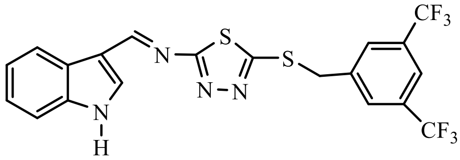

$C_{20}H_{12}F_6N_4S_2$, yellow solid powder； m. p. 201.3-202.2 ℃.

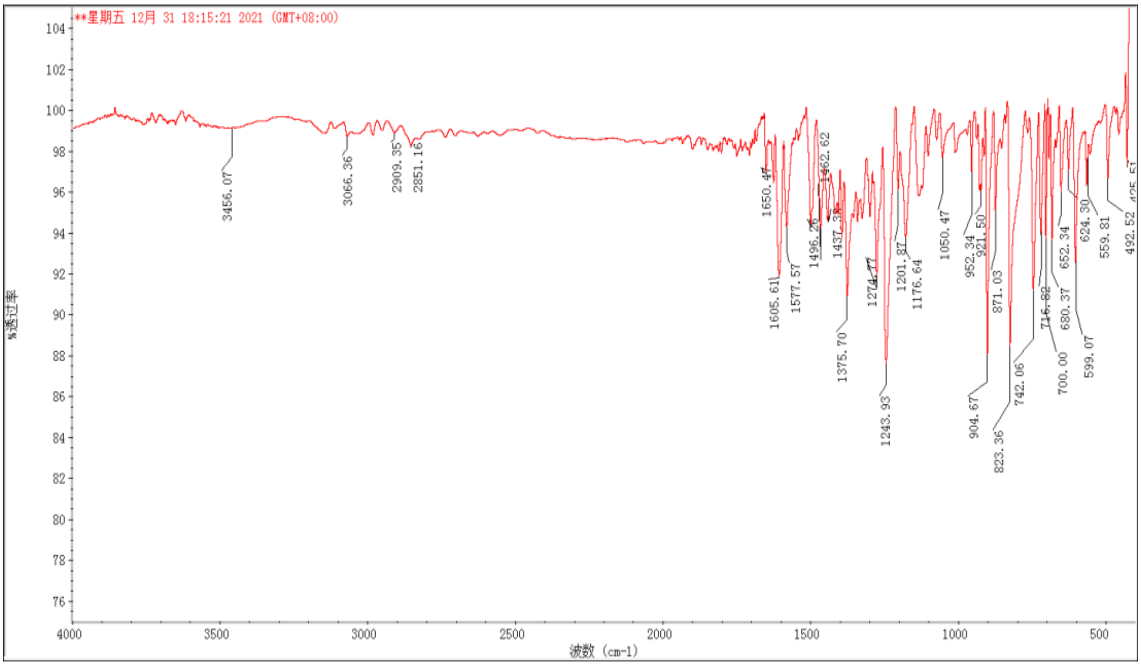

Figure S65. IR spectra of the compound **2q**

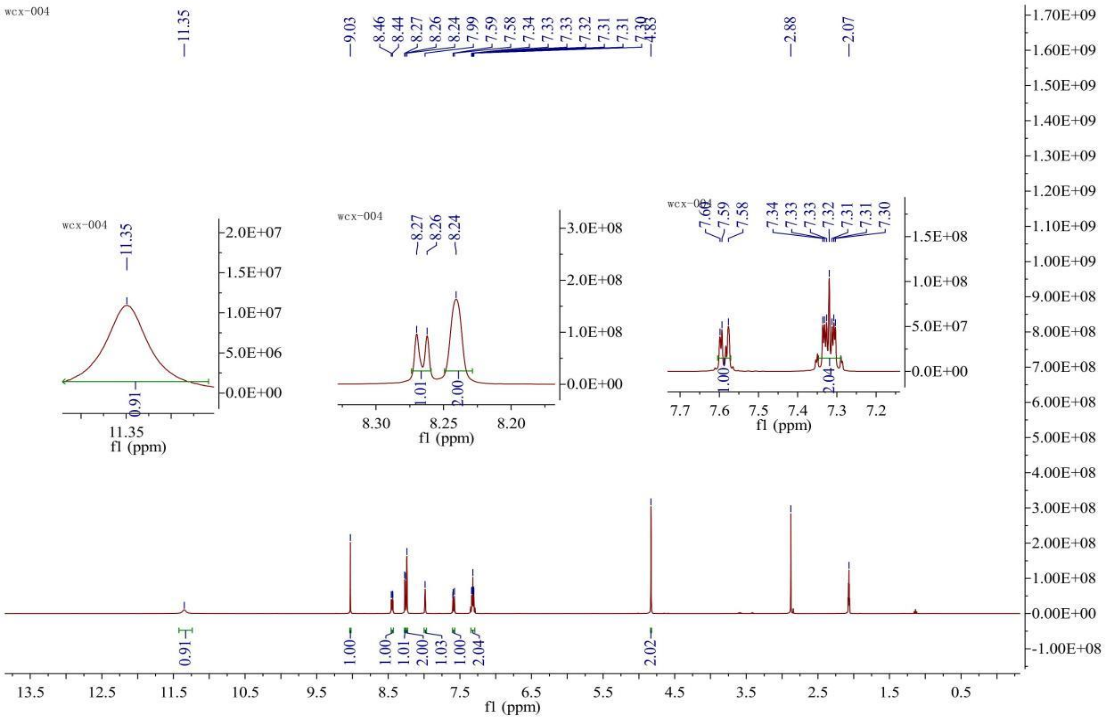

Figure S66. ¹H NMR spectra of the compound **2q** (Acetone)

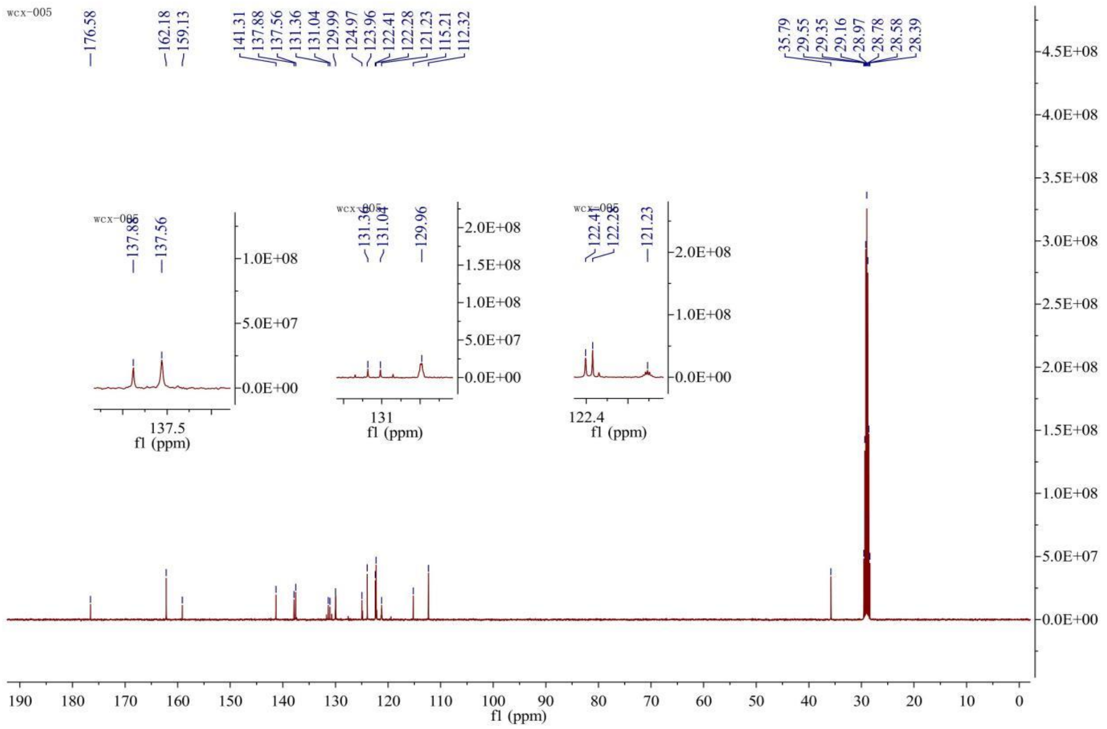

Figure S67. ^{13}C NMR spectra of the compound **2q** (Acetone)

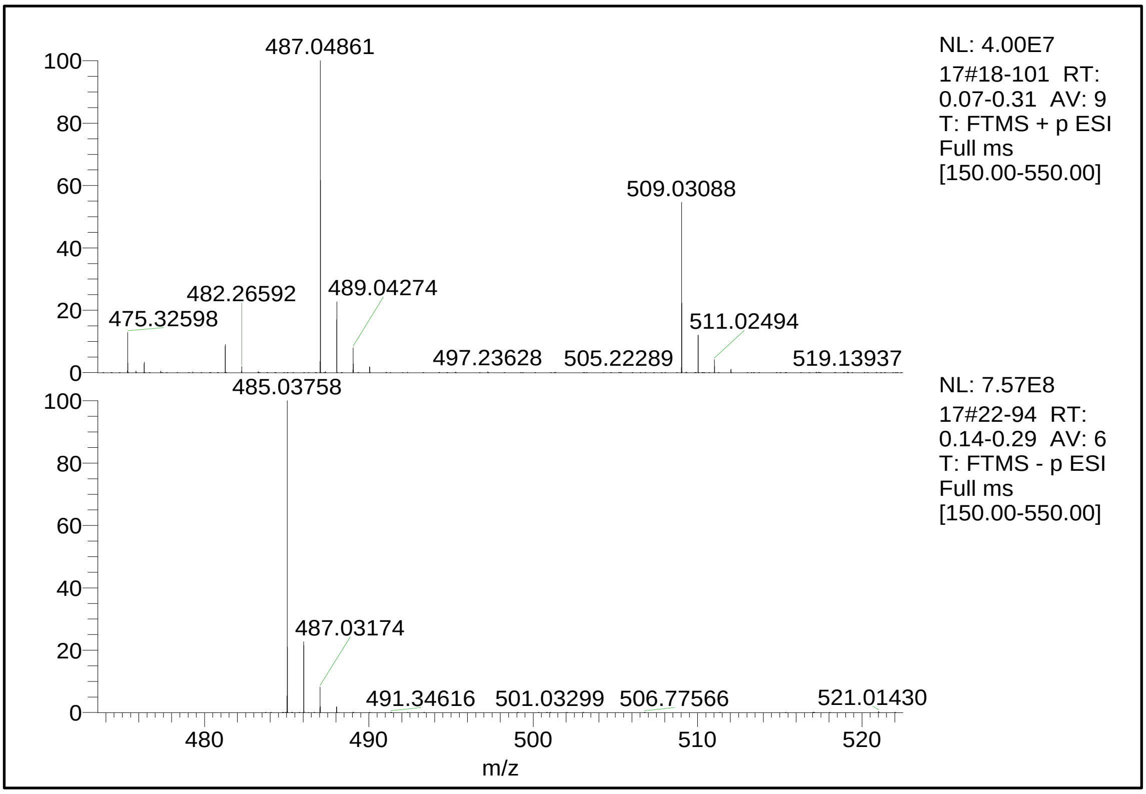

Figure S68. HR-MS of the compound **2q**

Calcd for $C_{20}H_{13}F_6N_4S_2$ $[M+H]^+$: 487.0486; found 487.0486.

18.The IR, ^{1}H NMR, ^{13}C NMR and HRMS of the compound 2r

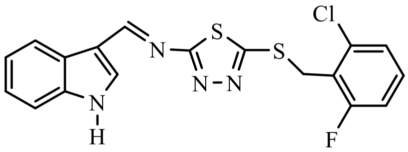

$C_{18}H_{12}ClFN_4S_2$, yellow solid powder; m. p. 194.6-195.5 ℃.

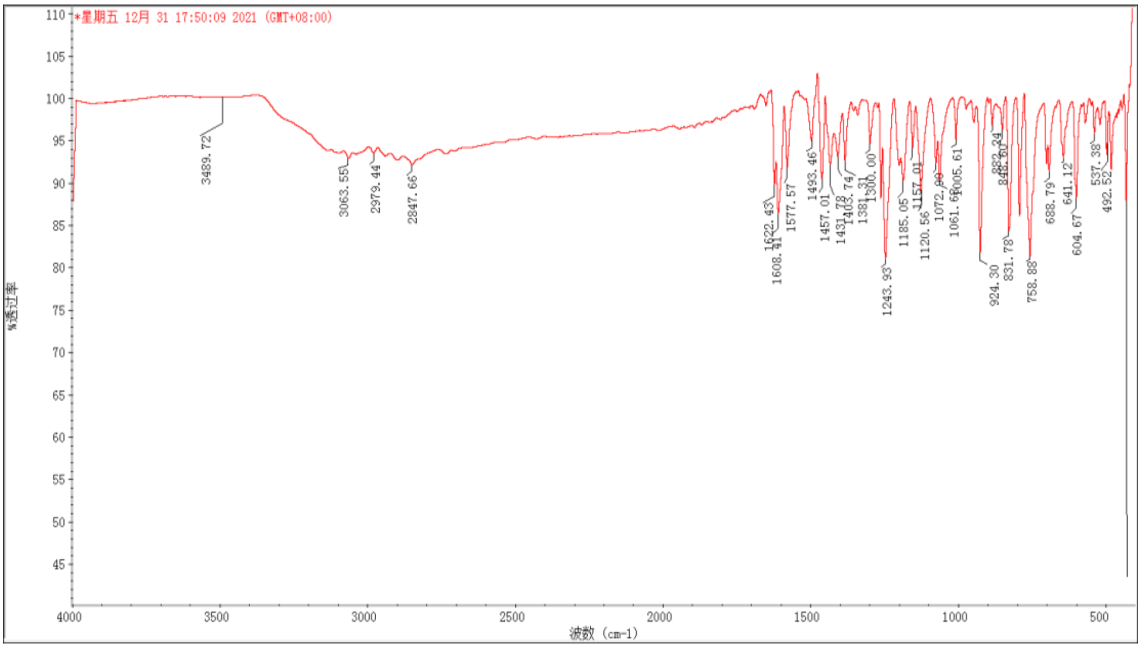

Figure S69. IR spectra of the compound **2r**

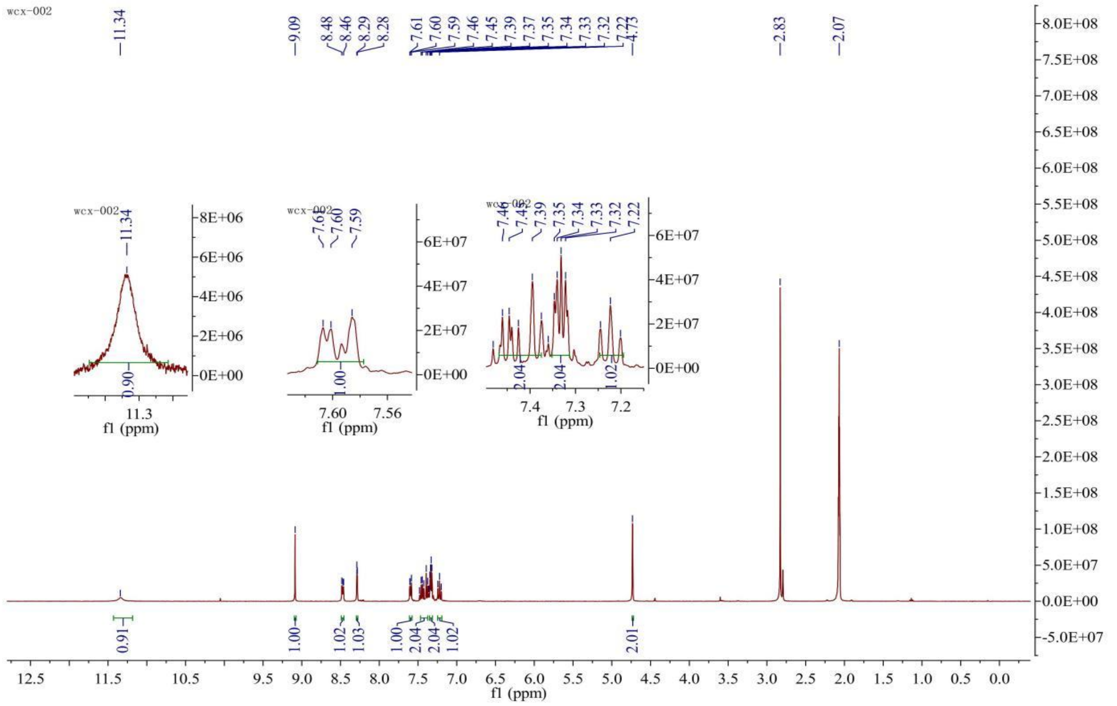

Figure S70. ^{1}H NMR spectra of the compound **2r** (Acetone)

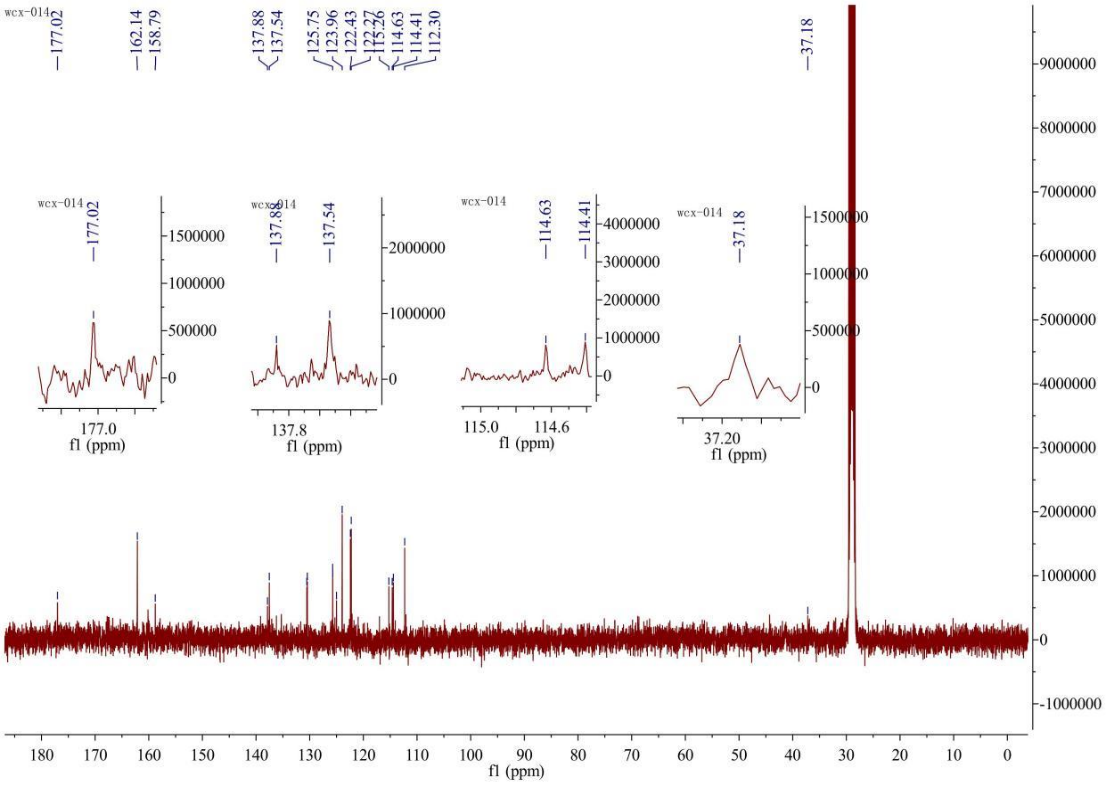

Figure S71. ^{13}C NMR spectra of the compound **2r** (Acetone)

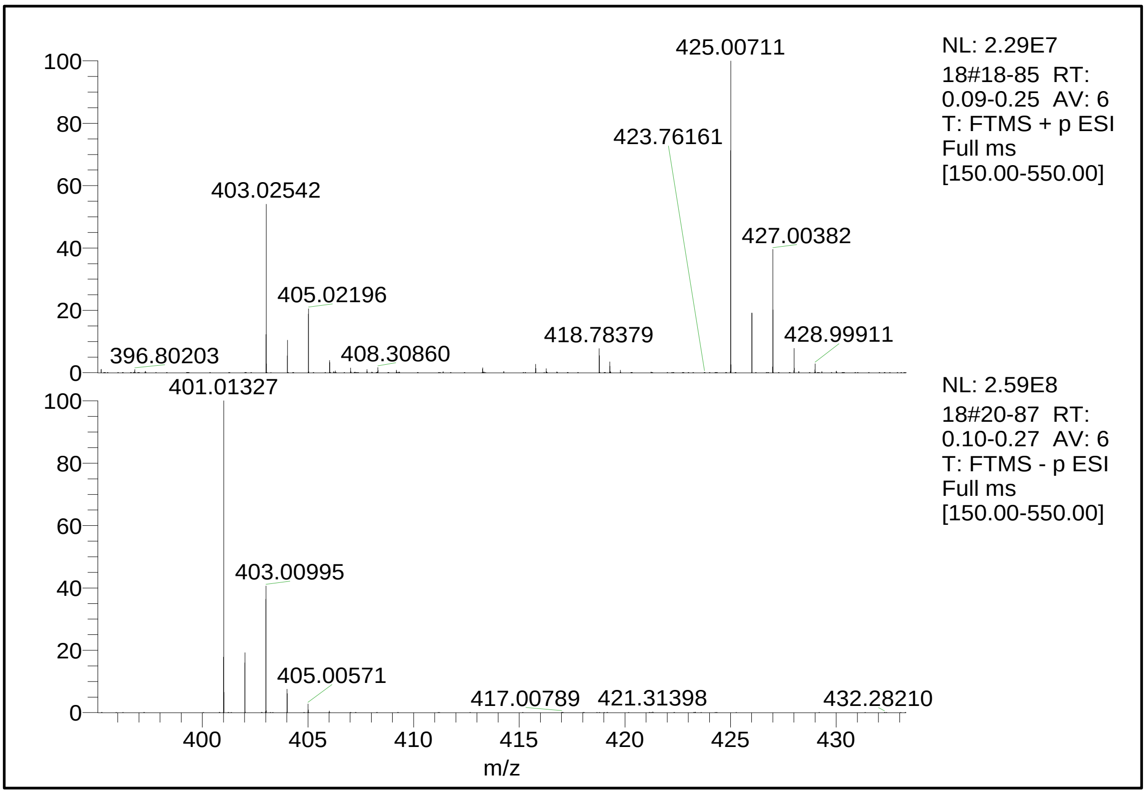

Figure S72. HRMS of the compound **2r**

Calcd for $C_{18}H_{12}ClFN_4S_2$ [M+ Na]$^+$: 425.0074; found 425.0071.

19.The IR, ¹H NMR, ¹³C NMR and HRMS of the compound 2s

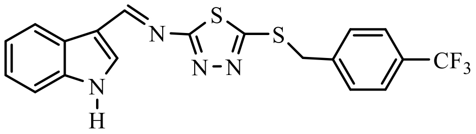

$C_{19}H_{13}F_3N_4S_2$, yellow solid powder; m. p. 198.1-199.0 ℃.

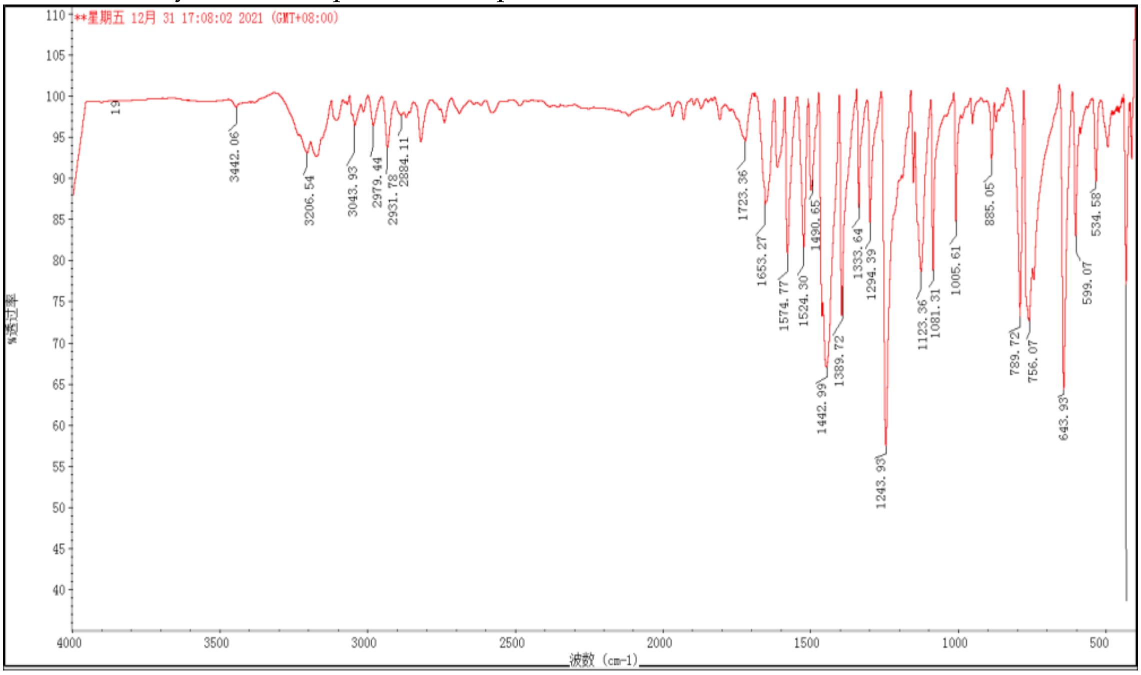

Figure S73. IR spectra of the compound **2s**

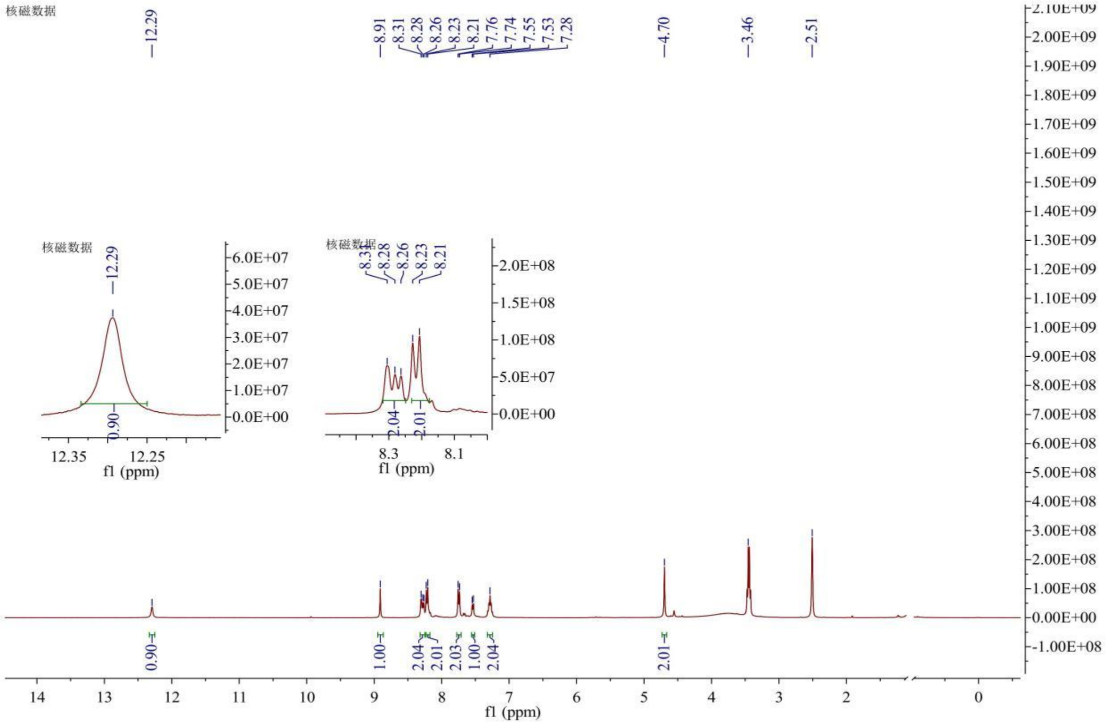

Figure S74. ¹H NMR spectra of the compound **2s** (Acetone)

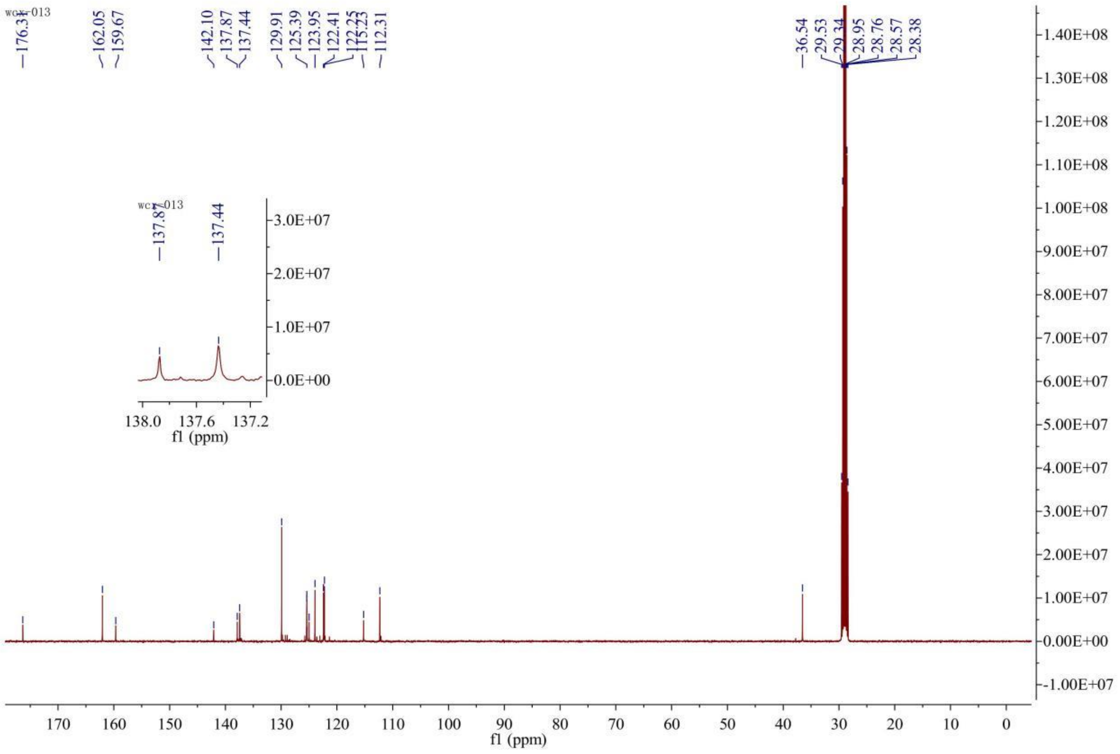

Figure S75. ^{13}C NMR spectra of the compound **2s** (Acetone)

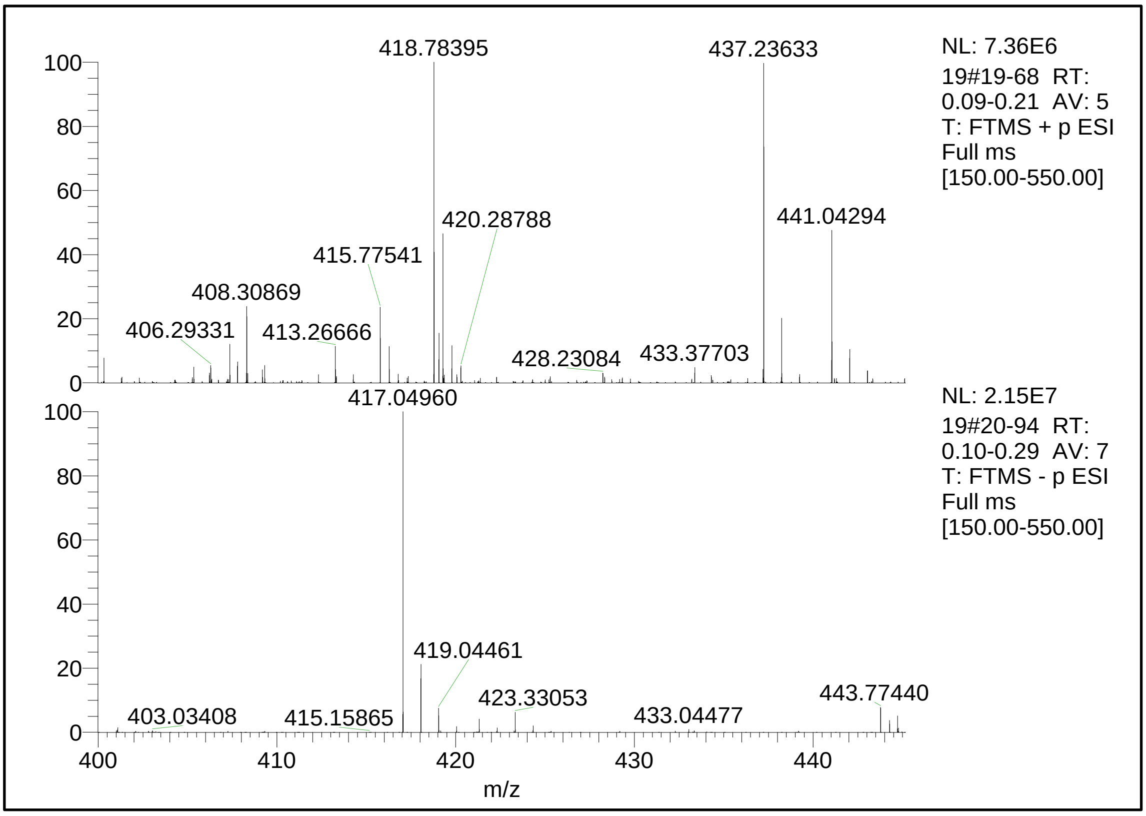

Figure S76. HRMS of the compound **2s**

Calcd for $C_{19}H_{12}F_3N_4S_2$ $[M-H]^+$: 417.0456; found 417.0496.

20.The IR, ^{1}H NMR, ^{13}C NMR and HRMS of the compound 2t

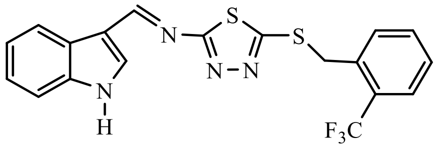

$C_{19}H_{13}F_3N_4S_2$, yellow solid powder； m. p. 194.7-195.6 ℃.

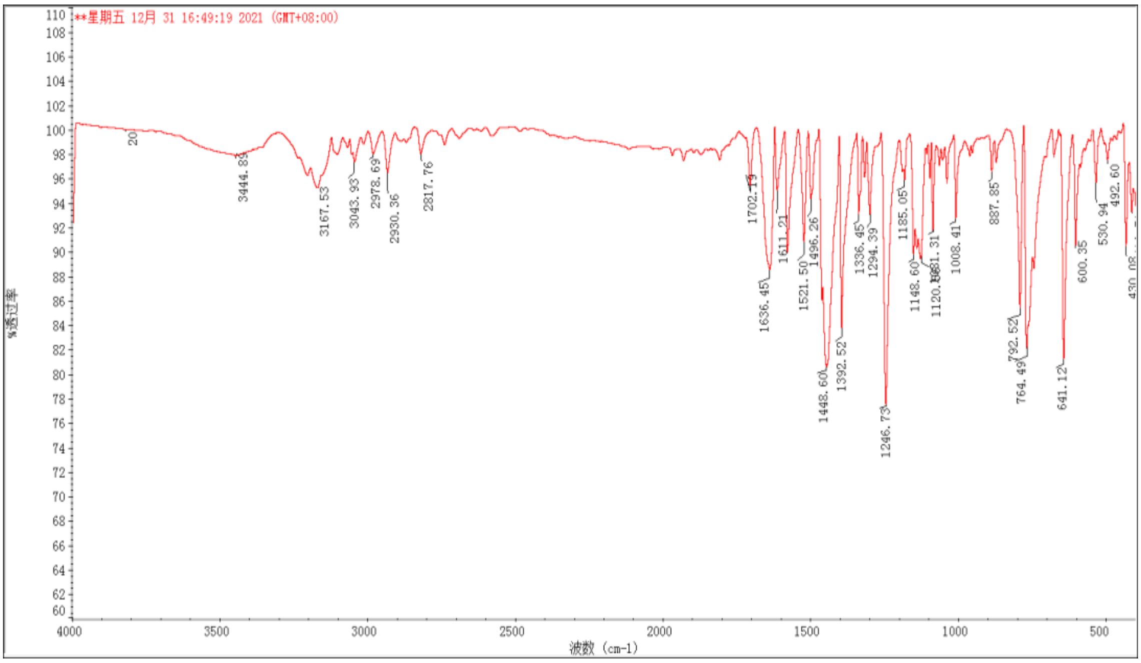

Figure S77. IR spectra of the compound **2t**

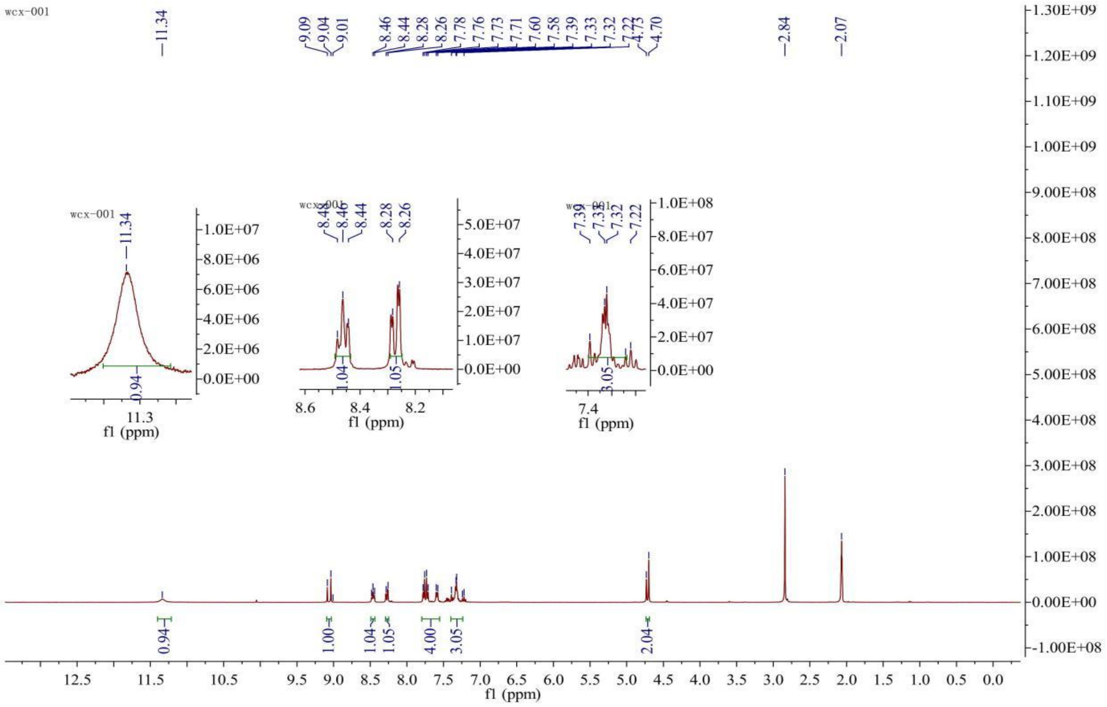

Figure S78. ^{1}H NMR spectra of the compound **2t** (Acetone)

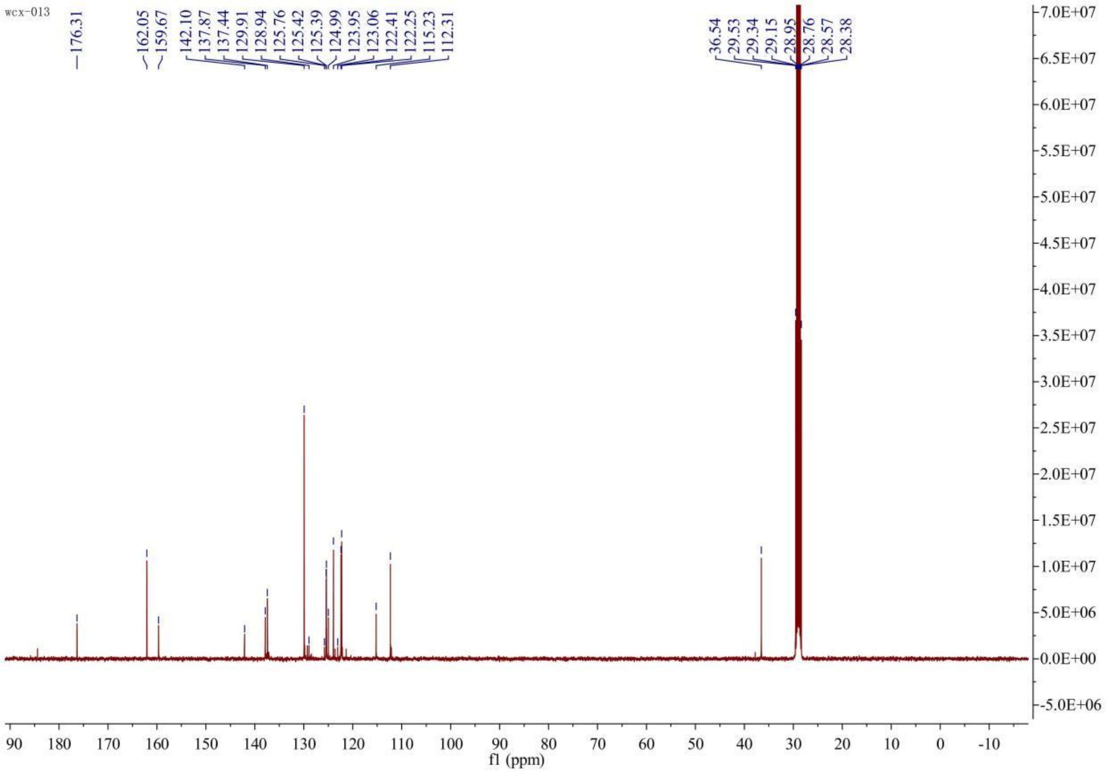

Figure S79. ^{13}C NMR spectra of the compound **2t** (Acetone)

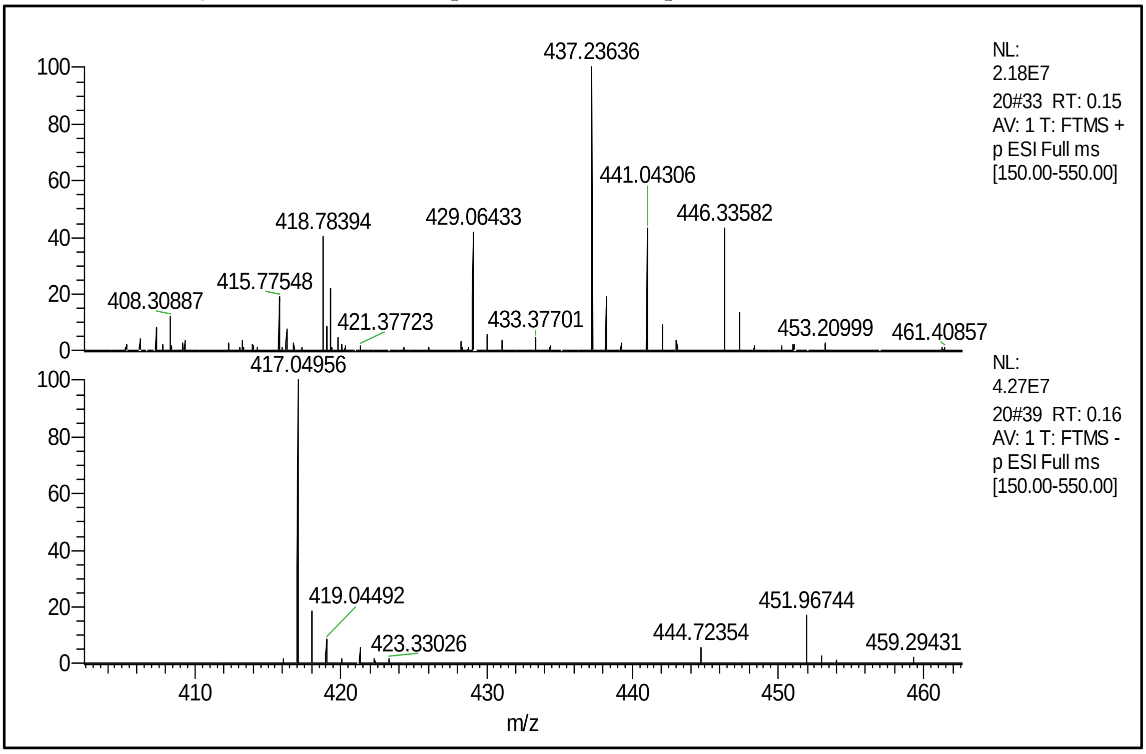

Figure S80 HRMS of the compound **2t**

Calcd for $C_{19}H_{12}F_3N_4S_2$ [M-H]$^+$: 417.0456; found 417.0496.

Ⅱ. The physical photos of the inhibitory activity of the target compounds against fungi, Figure S81-S85

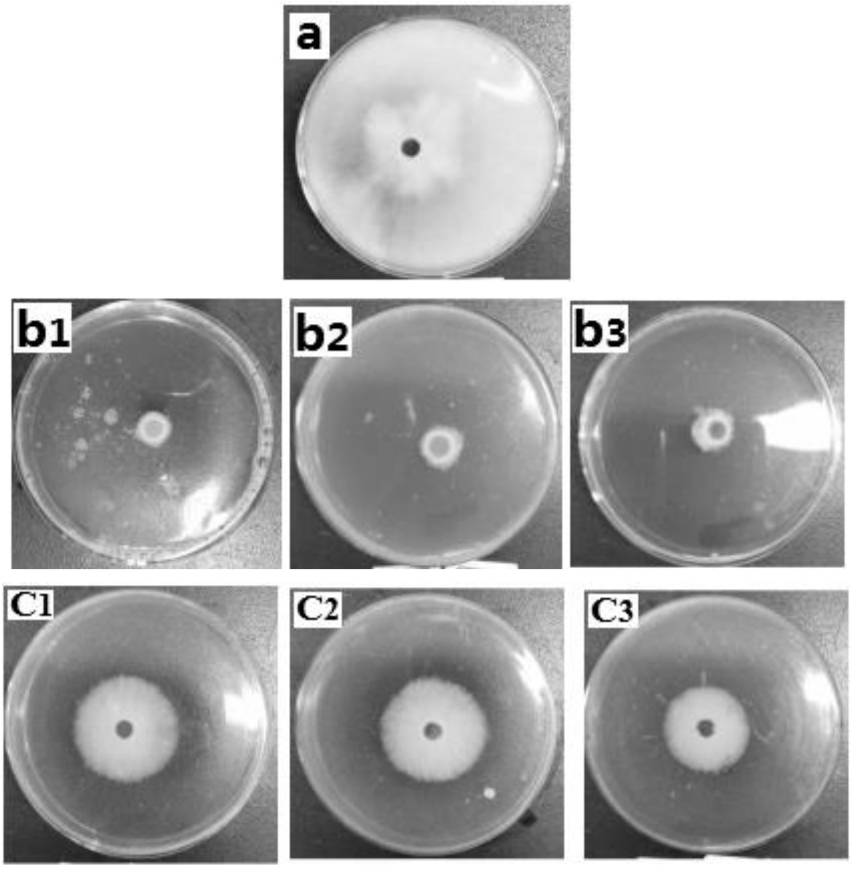

Figure S81. The physical photos of the inhibitory activity of compounds **2j** and **2q** against *F. graminearum*

Note: The photo **a** is the physical photo of no drug blank control; b_1, b_2, b_3 are the physical photos of the inhibitory activity of compound **2j** against *F. graminearum* under the same test conditions; c_1, c_2, c_3 are the physical photos of the inhibitory activity of compound **2q** against *F. graminearum* under the same test conditions.

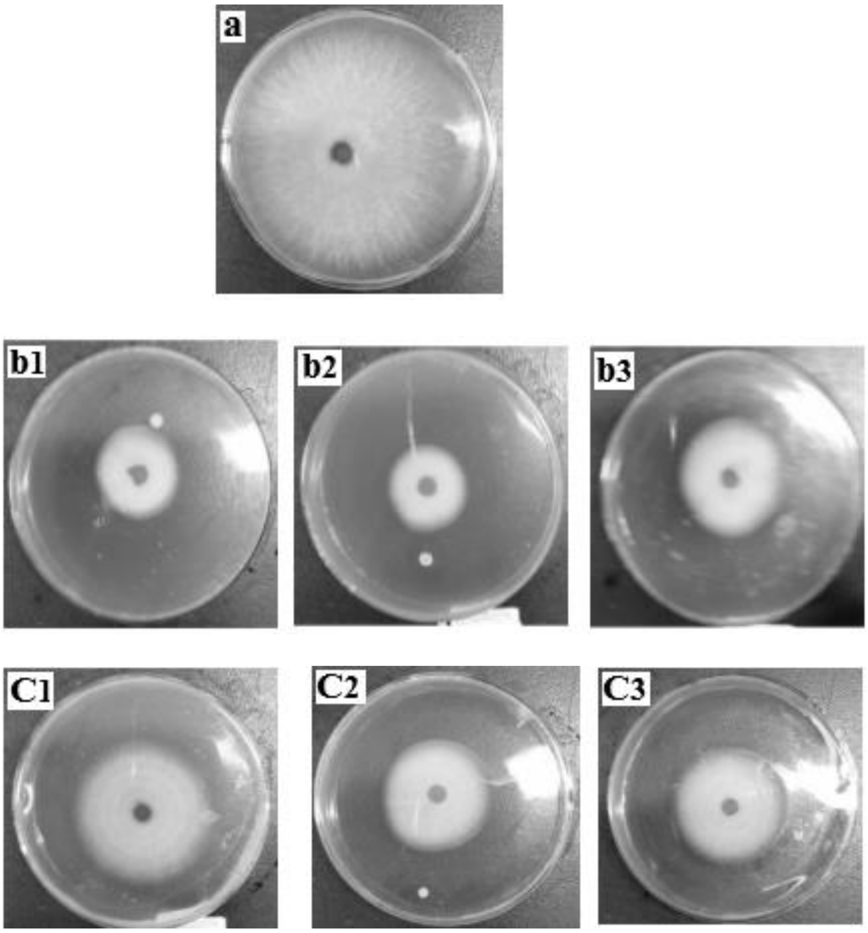

Figure S82. The physical photos of the inhibitory activity of compounds **2j** and **2o** against *F. oxysporum.*

Note: The photo **a** is the physical photo of no drug blank control; **b**$_1$**,** **b**$_2$**,** **b**$_3$ are the physical photos of the inhibitory activity of compound **2j** against *F. oxysporum* under the same test conditions; **c**$_1$**,** **c**$_2$**,** **c**$_3$ are the physical photos of the inhibitory activity of compound **2o** against *F. oxysporum* under the same test conditions.

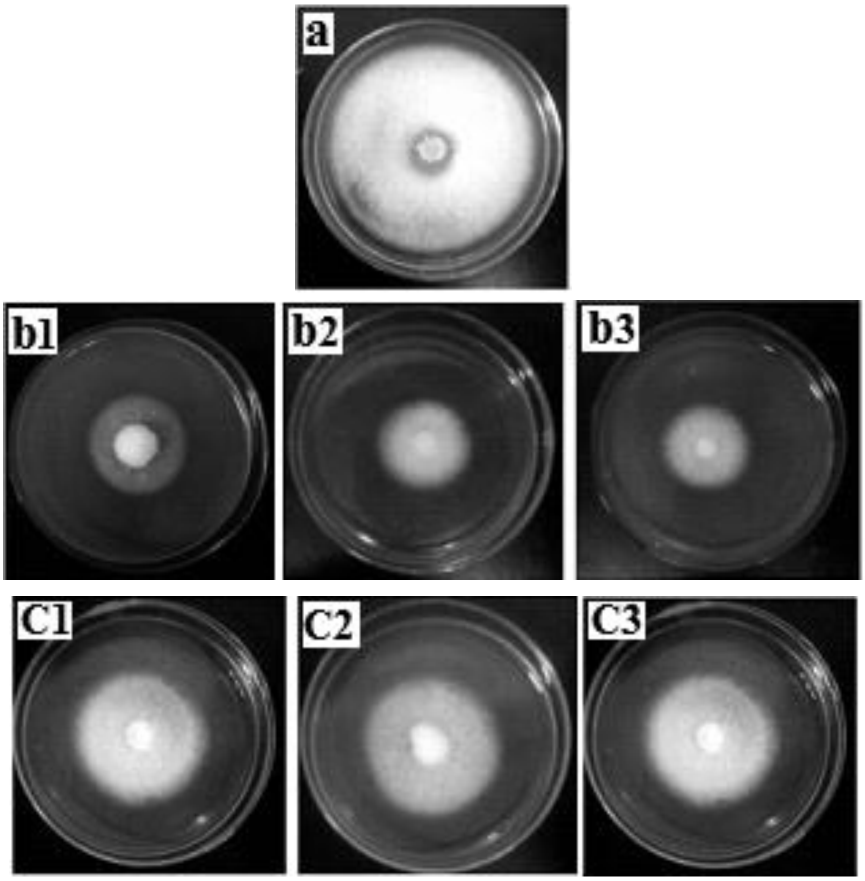

Figure S83. The physical photos of the inhibitory activity of compounds **2j** and **2o** against *F. moniliforme*

Note: The photo **a** is the physical photo of no drug blank control; **b_1, b_2, b_3** are the physical photos of the inhibitory activity of compound **2j** against *F. moniliforme* under the same test conditions; **c_1, c_2, c_3** are the physical photos of the inhibitory activity of compound **2o** against *F. moniliforme* under the same test conditions.

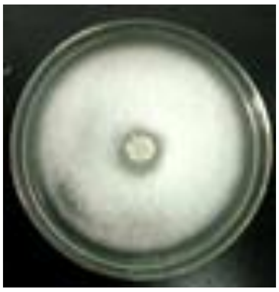

a

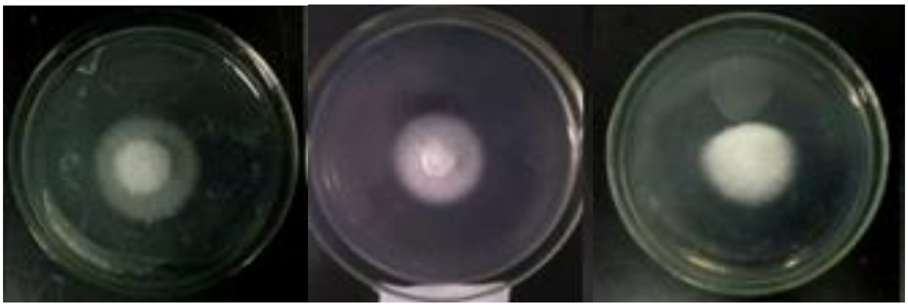

b1 b2 b3

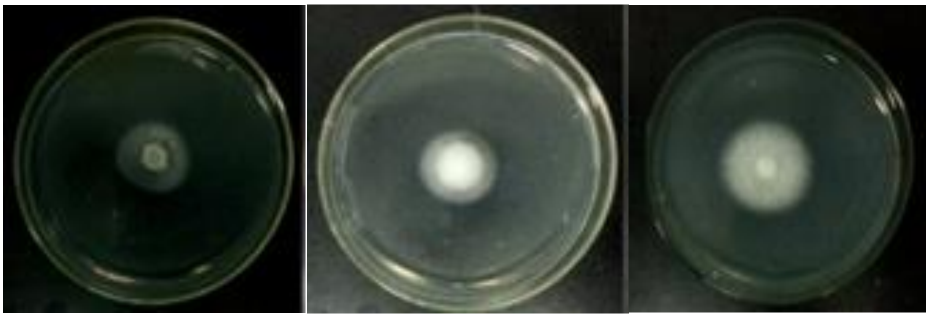

C1 C2 C3

Figure S84 The physical photos of the inhibitory activity of compounds **2j** and **2q** against *C. lunata*

Note: The photo **a** is the physical photo of no drug blank control; $\mathbf{b_1}$**,** $\mathbf{b_2}$**,** $\mathbf{b_3}$ are the physical photos of the inhibitory activity of compound **2j** against *C. lunata* under the same test conditions; $\mathbf{c_1}$**,** $\mathbf{c_2}$**,** $\mathbf{c_3}$ are the physical photos of the inhibitory activity of compound **2q** against *C. lunata* under the same test conditions.

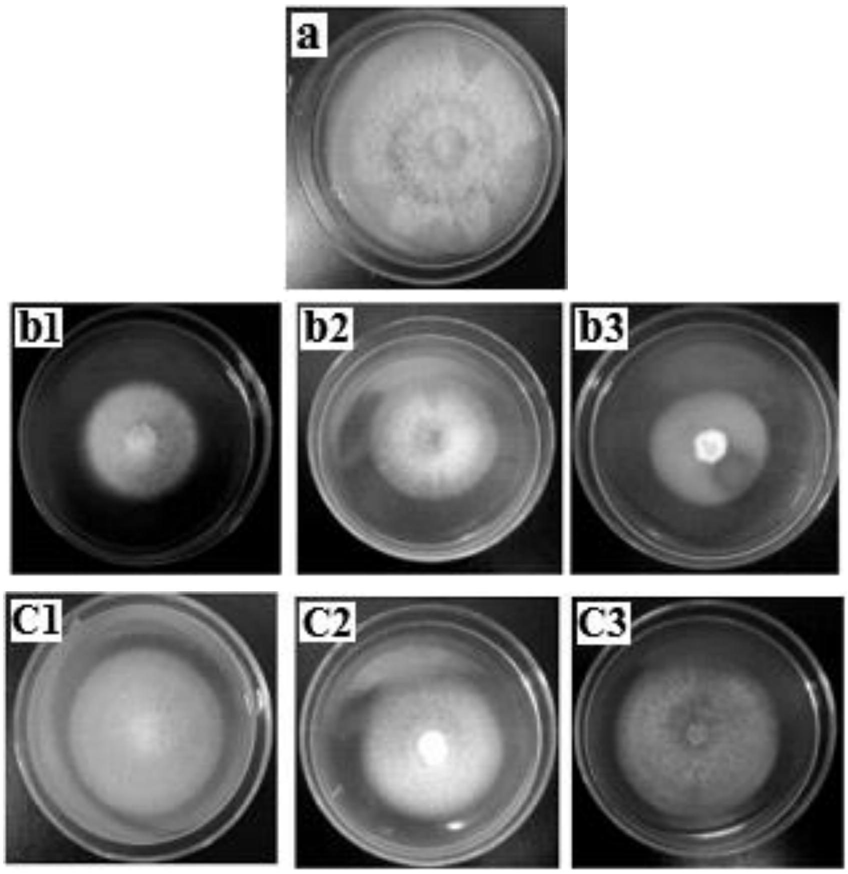

Figure S85. The physical photos of the inhibitory activity of compounds **2j** and **2n** against *P. p.* var. *nicotianae*

Note: The photo **a** is the physical photo of no drug blank control; **b**$_1$, **b**$_2$, **b**$_3$ are the physical photos of the inhibitory activity of compound **2j** against *P. p.* var. *nicotianae* under the same test conditions; **c**$_1$, **c**$_2$, **c**$_3$ are the physical photos of the inhibitory activity of compound **2n** against *P. p.* var. *nicotianae* under the same test conditions.
